# Organ-specific expression of genes associated with the UDP-glucose metabolism in sugarcane (*Saccharum* spp. hybrids)

**DOI:** 10.1186/s12864-023-09124-8

**Published:** 2023-01-13

**Authors:** Patrick J. Mason, Nam V. Hoang, Frederik C. Botha, Agnelo Furtado, Annelie Marquardt, Robert J. Henry

**Affiliations:** 1 Queensland Alliance for Agriculture and Food Innovation (QAAFI), Level 2, Queensland Biosciences Precinct [#80], The University of Queensland, St Lucia, QLD 4072 Australia; 2 Wageningen University and Research (WUR), PO Box 9101, Wageningen, 6700 HB The Netherlands; 3 Commonwealth Scientific and Industrial Research Organisation (CSIRO), Level 3, Queensland Biosciences Precinct [#80], The University of Queensland, St Lucia, QLD 4072 Australia

**Keywords:** UDP-glucose metabolism, Sugarcane, Sucrose synthase, UDP-glucose dehydrogenase, Invertase, Sucrose phosphate synthase, *Myo-*inositol oxygenase, Internodes, Roots, Leaves

## Abstract

**Background:**

The importance of uridine 5′-diphosphate glucose (UDP-G) synthesis and degradation on carbon (C) partitioning has been indicated in several studies of plant systems, whereby the kinetic properties and abundance of involved enzymes had a significant effect upon the volume of C moving into the hemicellulose, cellulose and sucrose pools. In this study, the expression of 136 genes belonging to 32 gene families related to UDP-G metabolism was studied in 3 major sugarcane organs (including leaf, internode and root) at 6 different developmental stages in 2 commercial genotypes.

**Results:**

Analysis of the genes associated with UDP-G metabolism in leaves indicated low expression of *sucrose synthase*, but relatively high expression of *invertase* genes, specifically *cell-wall invertase 4* and *neutral acid invertase 1–1* and *3* genes. Further, organs that are primarily responsible for sucrose synthesis or bioaccumulation, i.e., in source organs (mature leaves) and storage sink organs (mature internodes), had very low expression of sucrose, cellulose and hemicellulose synthesis genes, specifically *sucrose synthase 1* and *2*, *UDP-G dehydrogenase 5* and several *cellulose synthase* subunit genes. Gene expression was mostly very low in both leaf and mature internode samples; however, leaves did have a comparatively heightened *invertase* and *sucrose phosphate synthase* expression. Major differences were observed in the transcription of several genes between immature sink organs (roots and immature internodes). Gene transcription favoured utilisation of UDP-G toward insoluble and respiratory pools in roots. Whereas, there was comparatively higher expression of sucrose synthetic genes, *sucrose phosphate synthase 1* and *4*, and comparatively lower expression of many genes associated with C flow to insoluble and respiratory pools including *myo-Inositol oxygenase*, *UDP-G dehydrogenase 4*, *vacuolar invertase 1*, and several *cell-wall invertase*s in immature internodes.

**Conclusion:**

This study represents the first effort to quantify the expression of gene families associated with UDP-G metabolism in sugarcane. Transcriptional analysis displayed the likelihood that C partitioning in sugarcane is closely related to the transcription of genes associated with the UDP-G metabolism. The data presented may provide an accurate genetic reference for future efforts in altering UDP-G metabolism and in turn C partitioning in sugarcane.

**Supplementary Information:**

The online version contains supplementary material available at 10.1186/s12864-023-09124-8.

## Introduction

**Fig. 1:**
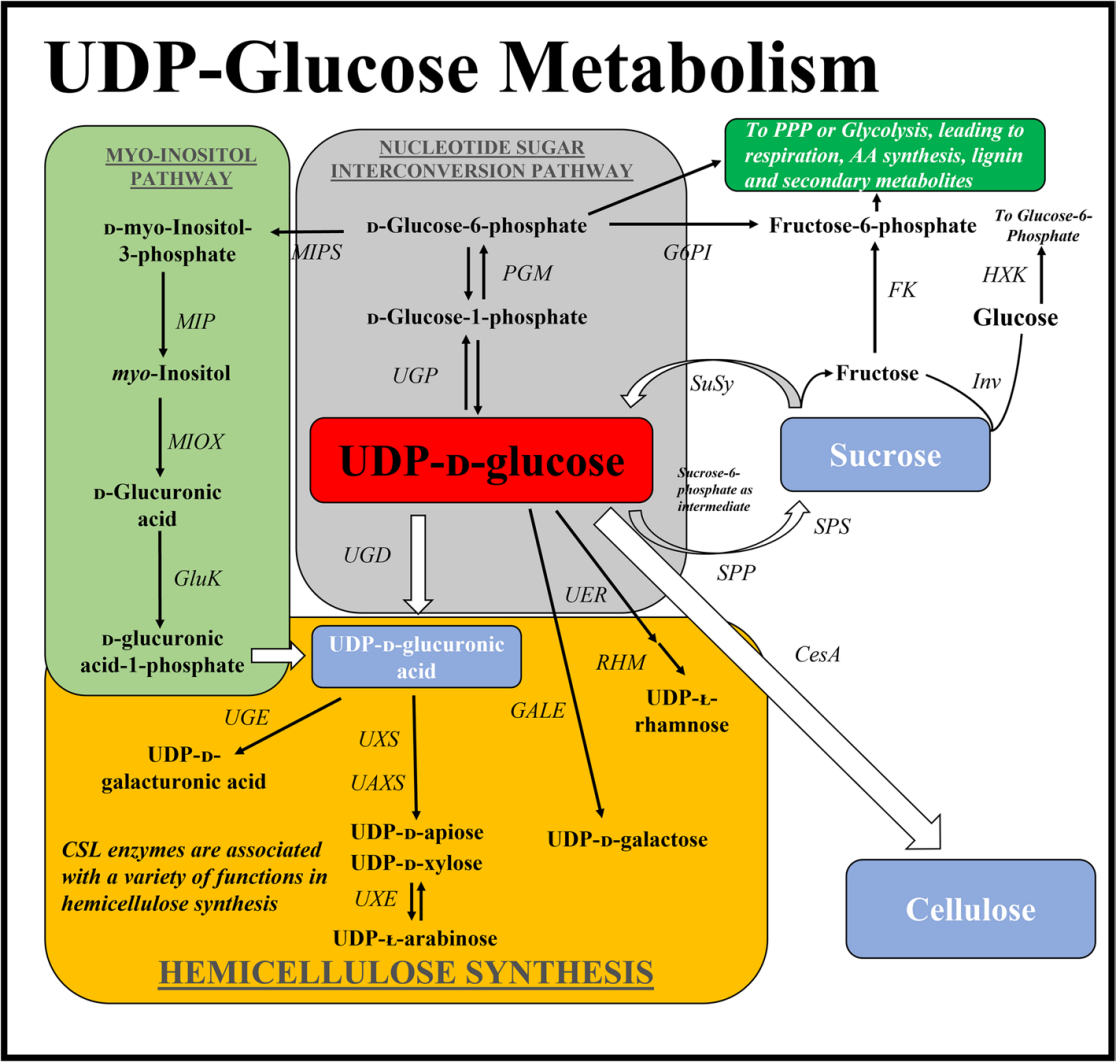
Metabolism associated with the synthesis/degradation of the UDP-glucose metabolite. Abbreviations, CWI: Cell Wall Invertase; VINV: Vacuolar Invertase; CINV: Cytosolic Invertase; ANINV: Alkaline/Neutral Invertase; FK: Fructokinase; MIPS: myo-Inositol phosphate synthase; MIP: myo-Inositol phosphatase; MIOX: myo-Inositol oxygenase; GluK: Glucuronokinase; PGM: Phosphoglucomutase; UGPase: Uridine Diphosphate Glucose pyrophosphorylase; UGD: UDP-Glucose Dehydrogenase; AGP: Adenosine Diphosphate Glucose pyrophosphorylase; CesA: Cellulose Synthase; CSL: Cellulose Synthase-Like; G6PI: Glucose-6-Phosphate Isomerase; SPP: Sucrose Phosphate Phosphatase; SPS: Sucrose Phosphate Synthase; SuSy: Sucrose Synthase; RHM: Uridine Diphosphate Glucose 4,6-dehydratase; UAXS: Uridine Diphosphate Glucose Apiose/Xylose Synthase; GALE: Uridine Diphosphate Glucose 4-Epimerase; UGE: Uridine Diphosphate Glucuronic Acid Epimerase; UXS: Uridine Diphosphate Xylose Synthase; UXE: Uridine Diphosphate Xylose Epimerase; HXK: Hexokinase

In plants, uridine 5′-diphosphate glucose (UDP-G) is a nucleotide sugar that is consumed irreversibly to produce the major components of the cell wall, cellulose and hemicellulose [1–4]. UDP-G can also be degraded into sucrose then resynthesized [5–7]. As UDP-G is directly synthesised into/or degraded from sucrose, it is likely that this nucleotide sugar’s consumption is also linked to lignin production, which occurs via the hydrolysis of sucrose leading into the glycolytic and oxidative pentose phosphate pathways [8]. Other roles for UDP-G in plants include its role as a glucose residue donor in callose formation, glycoproteins, glycolipids and sulpholipids, and its essential role in the glycosylation of steroids, flavonoids, betalains, glucosinolates and terpenoids [9]. Further, several studies have suggested a central role for UDP-G as a signal molecule regulating growth and development, biomass accumulation, and programmed cell death [8, 10–12]. The synthesis and degradation of the UDP-G metabolite is central to the amount of carbon (C) moving into the major pools within the sugarcane plant, specifically sucrose, cellulose and hemicellulose pools [7]. The importance of UDP-G control and its effect on C partitioning has been indicated in several studies of plant systems, whereby the kinetic properties and abundance of enzymes involved in UDP-G synthesis/utilisation had a significant effect upon the volume of C moving into the insoluble and soluble pools [8, 13–19], see Fig. 1 for a summary.

A gene family is a set of functionally similar genes formed via whole genome duplication and derived originally from a single ancestral gene [20]. The value for plant systems having developed multiple gene isoforms coding for the same enzyme is that often small alterations to enzyme structure can confer differing affinities to substrates and feedback inhibitory molecules, meaning the flux through this point in the pathway can continue in a variety of cellular conditions [4, 21, 22]. The value in understanding the full array of gene family isoforms was shown in an attempt to downregulate an important consumer of UDP-G into the hemicellulosic precursor UDP-glucuronic acid, via the activity of UDP-G dehydrogenase (UGD) in sugarcane. A sugarcane genetic knockout mutant of a single *UGD* gene was created in [23]. Cessation of UGD activity was expected to have a reductive effect on the hemicellulose fraction, however, no differences in composition were observed. Upregulation of the *myo*-inositol pathway, which provides an alternative pathway for C to enter hemicellulose synthesis, was detected in the mutant, which was used to explain the lack of compositional difference in comparison to the control [23]. Also, based on previous characterisations of the *UGD* gene family in other plants [4, 21], it is highly unlikely that there is a single *UGD* gene in the sugarcane genome, which may also have contributed to the reasons why no difference in cell wall composition was recorded in the *UGD* single mutant. Therefore, in sugarcane, successful alterations of UDP-glucuronic acid synthesis is likely contingent on changes to genes associated with the *myo*-Inositol pathway, but also the identification and alteration of additional *UGD* genes. In a related study within the *Arabidopsis* genome [14], of the 4 *UGD* genes, only double genetic knockout mutants of *UGD 2* and *UGD 3* genes combined led to reduced UDP-glucuronic acid synthesis. Suppression of UDP-glucuronic acid synthesis caused a significant decrease in the hemicelluloses arabinose, xylose, apiose and galacturonic acid, in turn causing dwarfism and other developmental defects in the double mutants [14]. Single knockout mutants of *UGD 2* or *UGD 3* did not affect the cell wall composition, which indicates the activity of only one of these genes is sufficient to make up for the lack of expression in the other, whilst the other isoforms likely had organ-specific functions. In a related study within Arabidopsis *UGD 2* and *3* had significantly higher expression than isoforms *1* and *4* in all tissues [21]. This result may suggest that gene family members with significantly higher expression have a larger effect on the pathway than isoforms with lower expression. This is also supported by the finding that each UDG isoform had differing affinity for the substrate. *UGD 3* had a far higher affinity and catalytic constant, suggesting it is responsible for the bulk of C assimilation into the hemicellulose pool [21], in turn explaining the adverse effects of knockouts. Studies of multi-gene families related to the UDP-G metabolism have reported similar results, whereby knockout mutants of 1, 2 or even 3 isoforms were found to have a disproportionate effect on the physiology of the mutant, relating to alterations in the cell wall [15, 24–30].

Modern high throughput technologies such as next-generation sequencing (NGS) allow a platform to look broadly at differences in gene expression in different tissues and organs [31]. In this study, to study the organ-specific expression of genes associated with the UDP-G metabolism in the sugarcane plant, we used mature sugarcane leaves (dedicated source organ), young internodes (sink organ where the bulk of UDP-G and hexose phosphates are utilised in cellulose, hemicellulose, protein synthesis and respiration), maturing and mature internodes (sink organ where respiration is limited and UDP-G is utilised in sucrose storage) and root (a meristematic sink that has similar C requirements to immature internodes) [32]. These differing metabolic requirements suggest the action of different mechanisms at the transcriptomic level in related metabolic pathways. As the metabolism of UDP-G is central to key sources of C deposition, it stands to reason that the expression of related genes would have differed expression as related to how C is utilised in that tissue. Additionally, most of these genes are in multi-gene families, and many multi-gene families likely contain isoforms that overall have a larger effect on metabolism than others. By determining which members are expressed and in which tissue, gene candidates for altering UDP-G metabolism and by proxy C metabolism could be identified.

## Materials and methods

### Plant material collection, data generation and transcript profiling

The plant material used in this experiment is derived from two commercial sugarcane genotypes, KQ228 and Q208 provided by Sugar Research Australia (SRA). Two sugarcane genotypes were analysed as an additional validatory mechanism. KQ228 and Q208 share common ancestry and agronomic traits, so they were expected to have comparable transcriptomic profiles [33]. Agronomic details of both genotypes can be found in Table S1, within Supplementary Data File 1. Leaf and internode samples of both genotypes were taken from 9-month old commercial stands. Root samples were obtained from 3-month old research plants in ‘soft’ above-ground pots. For sampling, schematic see [32]. Briefly, a total of 6 samples were collected at different developmental stages from three major sugarcane organs, including internodes, leaves and root. Hereafter, these samples are referred to as TI (Top Internode), MI (Middle Internode), BI (Bottom Internode), L1 (1st Visible Dewlap Leaf), L5 (5th Visible Dewlap Leaf) and R (Root). All samples were collected in triplicate and ‘snap frozen’ in liquid nitrogen within a minute of excision, put on dry ice in transit and kept in a − 80 °C freezer before preparation. Before RNA extraction samples were homogenized in cryogenic conditions. RNA extractions were conducted using the combined Trizol kit and RNeasy Plant minikit, as described in [34]. Samples were prepared with Illumina TruSeq™ stranded total RNA library prep kit, using 1000 ng of the submitted RNA, by QBI (Queensland Brain Institute, University of Queensland, St Lucia QLD, 4072). Each sample was sequenced in 3 lanes using an Illumina HiSeq2000 instrument to obtain 125 bp paired end reads. Read data was assessed using FastQC [35], to determine the quality and adapter sequences. A full-length sugarcane Iso-seq transcriptome database (SUGIT) developed by Hoang et al. 2017, was utilised as the reference sequence [36], in the assessment of transcript specific gene expression. The pipeline used to profile the transcriptome of the multiple samples in this experiment is based on the protocol ‘Expression Analysis using RNA-seq’ by QIAGEN bioinformatics [37] in the CLC Genomics Workbench version 12 (CLC-GWB, CLC Bio-QIAGEN, Aarhus, Denmark). The CLC-GWB analyses were conducted on a CLC Genomics Server, the CLC server, nodes and CLC-clients which are part of the Robert Henry Bioinformatics infrastructure at QAAFI, The University of Queensland, Australia. All CLC infrastructure was hosted by the Research Computing Centre (RCC), based at the University of Queensland, Australia [38]. For further details of plant collection and data generation, details see [39].

### Extraction of transcripts of interest and subsequent matching to gene family isoforms

A search for all transcripts associated with gene families related to the consumption/synthesis of the UDP-G metabolite was conducted in the sugarcane SUGIT transcriptome. Using CLC-GWB, relevant search terms, i.e. “sucrose synthase”, were queried against the SUGIT transcript annotations, to derive lists of relevant transcripts, which were extracted as FASTA files. Additional mapping was undertaken using the large-gap mapping tool in CLC-GWB (length fraction of 0.8 and similarity fraction of 0.5), whereby genes of interest from the *Sorghum bicolor* and *Zea mays* genomes were mapped against the SUGIT transcriptome, to ensure any mislabeled transcripts were included in downstream analysis. All extracted transcripts were blasted against the *S. bicolor* genome (accession number NC_012875.2) using NCBI’s nucleotide Basic Local Alignment Search Tool (BLAST) [40], with default settings in blastn, to group transcripts into their respective gene family homologues. The top BLAST hit based on query coverage and per cent identity was used to define the transcripts gene identity.

### Genomic location and gene length determination using the sugarcane STP monoploid genome

The sugarcane monoploid genome, a 382-Mb high-quality single-tiling-path (STP) sequence [41], enabled the genomic location and gene length to be determined in most of the genes of interest. The sequences of the genes of interest were also extracted and mapped to the extracted SUGIT transcripts (in CLC-GWB) to ensure paralogous genes (derived from recent genome duplications) were assigned the correct transcripts. To find the genes of interest within the sugarcane monoploid genome, the coding region of equivalent sorghum genes were blasted against the STP sequence. Sequences with high degrees of similarity were extracted then blasted against the sorghum genome to ensure the manual annotation was correct. Enzyme concession numbers for each gene family was defined by blasting the corresponding sorghum sequence in the UniProt database [42].

### Collapsing transcripts into a single expression value

The large degree of alternative splices and redundancy within the SUGIT reference transcriptome could reduce the accuracy of expression comparisons between the sugarcane organ types included in this study. Collapsing the normalised read counts (normalised using transcript per million (TPM) calculation) of all transcripts related to a gene, into a single value was deemed to be a far better option for determining the importance of specific gene family homologues, as the more isoforms a specific gene has in a reference transcriptome the less accurate differential gene expression comparisons will be [43]. The RNA-seq application within CLC-GWB assigns reads exclusively to the reference, which means in the case of a read sharing the same identity with two reference sequences it will be assigned to one of the references randomly. Due to this, quantifying the expression of a gene that has multiple isoforms, was simply done by adding the TPM values together to form a single TPM value (Fig. S1).

### Data processing of gene family expression values

The statistical significance of the mean of the biological replicates was calculated utilising the one-way ANOVA function, with the additional Tukey’s post-hoc *t*-test (honestly significant difference, HSD test) within IBM SPSS Statistics v27 (SPSS Inc., Chicago, IL, USA). The null hypothesis was accepted at a *p*-value of > 0.05. Minimum and maximum outliers (two-sided) were removed from some datasets using the modified Thompson Tau test [44]. For one-way ANOVA and Tukey *t*-test values, see Table S1 and Figs. S2, S3, S4, S5, S6, S7, S8, S9 and S10.

## Results

### Identification of gene families associated with UDP-glucose metabolism in sugarcane

A total of 136 genes from 32 gene families associated with the UDP-G metabolism, were identified within the STP sugarcane monoploid genome (see Fig. 1 for a depiction of the associated pathway). Most of these genes were also represented by 560 transcripts in the long-read SUGIT transcriptome. It must be noted that some of the transcripts represented in the SUGIT transcriptome were not represented in the STP genome, which accounted for 27 genes (Table 1). Comparisons to equivalent genes in the *S. bicolor* genome identified several likely gene duplications in *ADP-glucose pyrophosphorylase (AGP)*, *cellulose synthase-like (CSLE)*, *cytosolic invertase (CINV)*, *sucrose synthase (SuSy)*, and *UDP-G-4-epimerase (GALE)* gene families in the sugarcane genome, otherwise known as paralogues [45, 46]. Among the 136 genes, 10 gene homologues/isoforms belonged to the *AGP* gene family. Within the *cellulose synthase (CESA)* and *cellulose synthase-like (CSL)* subfamilies, 10 genes belonged to the *CESA* subgroup, and 5, 4, 4, 7, 2, 1 and 2 genes in *CSL* sub groups A, C, D, E, F and H, respectively. Of the four *invertase* sub-families, we identified 3 *cytosolic invertase* (*CINV)* genes, 5 *alkaline/neutral invertase (ANINV)*, 1 *vacuolar invertase (VINV)* and 8 *cell wall invertase* (*CWI) I*. Note that CINV and ANINV are both known as “neutral invertases”, however, they have been annotated as different genes in the *S. bicolor* genome. Gene families associated with the *myo-*inositol pathway including *glucuronokinase (GluK)*, *myo-inositol phosphatase (MIP)* and *myo-inositol oxygenase (MIOX)* each only had a single gene within the monoploid genome, whilst the *MIPS* had two gene family members. Gene families associated with hemicellulose synthesis had varying numbers of associated gene family members. A single gene was identified for *uridine diphosphate glucose apiose/xylose synthase (UAXS)*, whilst multiple genes were identified in the *UGD, GALE, UDP-xylose synthase (UXS)*, *UDP-xylose epimerase (UXE), UDP-G 4,6-dehydratase (RHM)* and *uridine diphosphate glucuronic acid epimerase (UGE)* gene families, with 3, 5, 6, 3, 3, 4 members, respectively. Single chloroplastic, cytoplasmic and 2 bi-functional *phosphoglucomutase (PGM)* genes were identified, while 5 *UDP-G pyrophosphorylase (UGPase)* genes, 3 *glucose-6-phosphate isomerase (G6PI)*, 8 *hexokinase (HXK)* and 3 *fructokinase (FK)* genes were also identified. Some genes had large numbers of transcripts represented in the SUGIT transcriptome with *MIPS 2*, *SuSy 1*, *SuSy 2*, *SuSy 4, UGD 4, UGD 5, UGPase 2, CesA 5, CSLE 6–2, FK 2,* and *GALE 1* all had over 15 representative transcripts each.

**Table 1:** Ordered list of gene families and homologues found within the sugarcane monoploid genome associated with UDP-glucose metabolism

Gene family	***Gene name***	Gene identifier in sugarcane genome (Garmeur et al. 2018) [41]	Position in the sugarcane genome	Length	Sorghum NCBI transcript sequence accession No. & link	No. of transcripts in SUGIT transcriptome
**ADP-Glucose pyrophosphorylase** (EC No. 2.7.7.27)	*AGP 1–1*	Sh03_t015880	Sh03:28291165..28294172 (+ strand)	3008	XM_002455967.2	2*
	*AGP 1–2*	Sh03_t015870	Sh03:28260610..28265076 (+ strand)	4467	XM_002455967.2	2*
	*AGP 2–1*	Sh09_t019370	Sh09:34396677..34402313 (+ strand)	5637	XM_021448378.1	12*
	*AGP 2–2*	Sh01_t024650	Sh01 Sh01:42078537..42080613 (− strand)	2077	XM_021448378.1	12*
	*AGP 2–3*	Sh01_t024670	Sh01 Sh01:42115922..42117998 (− strand)	2077	XM_021448378.1	12*
	*AGP 3*	Sh02_t005750 & Sh02_t005760	Sh02 Sh02:9491664..9495697 (− strand)	3784	XM_021452431.1	0
	*AGP 4*	Not found in monoploid genome	n/a	n/a	XM_021465103.1	6
	*AGP 5*	Not found in monoploid genome	n/a	n/a	XM_002462095.2	6
	*AGP 6*	Not found in monoploid genome	n/a	n/a	XM_002463876.2	4
	*AGP 7*	Not found in monoploid genome	n/a	n/a	XM_021465103.1	2
**Cellulose Synthase** (EC No. 2.4.1.12)	*CesA 1–1*	Sh09_t005560 & Sh09_t005570	Sh09:9351086..9355687 (− strand)	6842	XM_002440649.2	11
	*CesA 1–2*	Sh03_t003840	Sh03:6782646..6790013 (+ strand)	7368	XM_002455055.2	7
	*CesA 2*	Not found in monoploid genome	n/a	n/a	XM_021446681.1	3
	*CesA 3*	Not found in monoploid genome	n/a	n/a	XM_021453750.1	14
	*CesA 4*	Sh03_t021020 & Sh03_t021030	Sh03:36782684..36788213 (+ strand)	5529	XM_002456316.2	6
	*CesA 5*	Sh10_t013650	Sh10:25483024..25488997 (+ strand)	5974	XM_021450574.1	19
	*CesA 6*	Sh02_t006050	Sh02:10077014..10082433 (+ strand)	5420	XM_002459590.2	8
	*CesA 7*	Sh01_t017850	Sh01:28983708..28987579 (− strand)	3872	XM_002467064.2	6
	*CesA 8*	Sh_229N08_t000100	Not in STP only found in BAC sequences	5414	XM_021452948.1	4
	*CesA 9*	Sh02_t013200	Sh02:25312245..25316453 (+ strand)	4209	XM_002460184.2	8
**Cellulose Synthase-Like A** (EC No. 2.4.1.32)	*CSLA 1*	Sh04_t005720	Sh04:8606968..8613408 (− strand)	6441	XM_002453415.2	2
	*CSLA 2*	Sh01_t020080	Sh01:32953742..32958781 (+ strand)	5040	XM_021451851.1	0
	*CSLA 3*	Sh02_t009410	Sh02:16752941..16756026 (+ strand)	3086	XM_002459829.2	0
	*CSLA 6*	Sh04_t016980	Sh04:29898814..29901757 (+ strand)	2944	XM_021458643.1	0
	*CSLA 7*	Sh02_t028710	Sh02:48277289..48281992 (+ strand)	4704	XM_002461027.2	1
**Cellulose Synthase-Like C** (EC No. 2.4.1)	*CSLC 1*	Sh03_t022070	Sh03:38066914..38070773 (− strand)	3860	XM_021457183.1	0
	*CSLC 2*	Sh_029O19	Not in STP only found in BAC sequences	2475	XM_021454662.1	1
	*CSLC 7*	Sh09_t015100	Sh09:28563626..28566886 (+ strand)	3261	XM_021447983.1	1
	*CSLC 9*	Sh01_t005210	Sh01:7634941..7643711 (+ strand)	8771	XM_021448173.1	3
**Cellulose Synthase-Like E** (EC No. 2.4.1)	*CSLE 2*	Sh04_t018310	Sh04:31810139..31814041 (− strand)	3903	XM_021460485.1	0
	*CSLE 6–1*	Sh02_t015890	Sh02:30256283..30259442 (− strand)	3160	XM_002462490.2	7
	*CSLE 6–2a*	Sh02_t015810	Sh02:30148511..30154022 (− strand)	5512	XM_002462489.2	15*
	*CSLE 6–2b*	Sh02_t015820	Sh02:30158577..30163879 (− strand)	5303	XM_002462489.2	15*
**Cellulose Synthase-Like F** (EC No. 2.4.1.34)	*CSLF 1*	Sh02_t024280 & Sh02_t024290	Sh02:42020371..42021308 (− strand)	1226	XM_002462951.2	0
	*CSLF 3–1*	Sh02_t024300, Sh02_t024310 & Sh02_t024320	Sh02:42028378..42030117 (− strand)	2191	XM_021452870.1	2*
	*CSLF 3–2*	Sh02_t024330	Sh02:42047848..42050979 (− strand)	3132	XM_021452421.1	2*
	*CSLF 3–3*	Sh02_t024380, Sh02_t024390 & Sh02_t024400	Sh02:42134676..42135020 (− strand)	2754	XM_021454665.1	2*
	*CSLF 6*	Not found in monoploid genome	n/a	n/a	XM_002445057.2	5
	*CSLF 8*	Not found in monoploid genome	n/a	n/a	XM_021454445.1	2
	*CSLF 9*	Sh02_t024360 & Sh02_t024370	Sh02:42109930..42111354 (+ strand)	3286	XM_021452834.1	0
**Cellulose Synthase-Like D** (EC No. 2.4.1)	*CSLFD 1*	Sh01_t021550	Sh01:36986329..36990074 (− strand)	3746	XM_002467380.2	2
	*CSLFD 2*	Not found in monoploid genome	n/a	n/a	XM_002436311.2	2
**Cellulose Synthase-Like G** (EC No. 2.4.1)	*CSLG 2*	Sh03_t033750, Sh03_t033760, Sh03_t033770 & Sh03_t033780	Sh03:54594123..54601044 (− strand)	6921	XM_021455434.1	1
**Cellulose Synthase-Like H** (EC No. 2.4.1)	*CSLH 1–1*	Not found in monoploid genome	n/a	n/a	XM_021463400.1	1
	*CSLH 1–2*	Not found in monoploid genome	n/a	n/a	XM_021463399.1	1
**Cytosolic Invertase** (EC No. 3.2.1.26)	*CINV 1–1*	Sh04_t018360	Sh04:31829368..31832935 (+ strand)	3568	XM_002452587.2	5*
	*CINV 2*	Sh04_t002850	Sh04:3999533..4003239 (− strand)	3707	XM_002453920.2	1
	*CINV 1–2*	Sh02_t022210	Sh02:39433217..39439275 (− strand)	6059	XM_002452587.2	5*
**Alkaline/Neutral Invertase** (EC No. 3.2.1.26)	*ANINV F1*	Not found in monoploid genome	n/a	n/a	XM_002450357.2	0
	*ANINV F2*	Sh04_t003010	Sh04:4348910..4351981 (+ strand)	3072	XM_002451407.2	3
	*ANINV 1–1*	Sh01_t031210	Sh01:52365269..52368792 (+ strand)	5203	XM_002465314.2	1
	*ANINV 1–2*	Sh03_t010910	Sh03:18378830..18382742 (+ strand)	3913	XM_002455539.2	7
	*ANINV 3*	Sh04_t011120	Sh04:20023276..20026209 (+ strand)	2934	XM_002452150.2	3
**Cell Wall Invertase** (EC No. 3.2.1.26)	*CWI 1*	Sh_226D11	In peripheral contigs (Sh_226D11:88740..93318)	4579	XM_021459751.1	6*
	*CWI 2–1*	Sh06_t003700	Sh06:8281155..8284931 (+ strand)	3777	XM_002489067.2	1*
	*CWI 2–2*	Sh06_t003690	Sh06:8271558..8274605 (− strand)	3048	XM_002489061.2	1*
	*CWI 2–3*	Not found in monoploid genome	n/a	n/a	XM_002489065.2	1*
	*CWI 3*	Sh01_t007690 & Sh01_t007700	Sh01:10946034..10947984 (− strand)	1895	XM_021451676.1	0
	*CWI 4*	Not found in monoploid genome	n/a	n/a	XM_021455138.1	3
	*CWI 5*	Not found in monoploid genome	n/a	n/a	XM_002448667.2	1
	*CWI 7*	Not found in monoploid genome	n/a	n/a	XM_002448668.2	1
**Vacuolar Invertase** (EC No. 3.2.1.26)	*VINV*	Sh06_t010830	Sh06:19959815..19964282 (+ strand)	4468	XM_002446812.2	**1**
**Fructokinase** (EC No. 2.7.1.4)	*FK 1*	Sh03_t028370	Sh03:47224065..47227404 (− strand)	3340	XM_002458864.2	2
	*FK 2*	Sh_206L06_t000080	Not in STP only found in BAC sequences	3072	XM_021464799.1	16
	*FK 6*	Not found in monoploid genome	n/a	n/a	XM_002436715.2	1
**Glucuronokinase** (EC No. 2.7.1.43)	*GlcK*	Sh08_t000060 & Sh08_t000070	Sh08:47728..50793 (+ strand)	2983	XM_002441606.2	11
**Glucose-6-Phosphate Isomerase** (EC No. 5.3.1.9)	*G6PI (Chloroplastic) 1*	Not found in monoploid genome	n/a	n/a	XM_002462464.2	2
	*G6PI (Chloroplastic) 2*	Sh02_t015090, Sh02_t015100, Sh02_t015110, Sh02_t015120 & Sh02_t015120	Sh02:28752694..28762708 (+ strand)	10,014	XM_002462464.2	5
	*G6P1 (Cytoplasmic) 1*	Not found in monoploid genome	n/a	n/a	XM_021448861.1	9
**Myo-inositol oxygenase **(EC No. 1.13.99.1)	*MIOX*	Not Found in monoploid genome	n/a	n/a	XM_021449433.1	12
**Myo-inositol phosphatase** (EC No. 3.1.3.25)	*MIP 3*	Sh01_t014550	Sh01:22505310..22508087 (− strand)	2778	XM_002466826.2	8
**Myo-inositol phosphate synthase** (EC No. 5.5.1.4)	*MIPS 1*	Sh01_t020350 & Sh01_t020360	Sh01:33923258..33924805 (− strand)	3661	XM_021462678.1	0
	*MIPS 2*	Not Found in monoploid genome	n/a	n/a	XM_021453593.1	27
**Phosphoglucomutase** (EC No. 5.4.2.2)	*PGM (chloroplastic) 1*	Sh03_t015170	Sh03:27010632..27018435 (− strand)	7804	XM_002466531.2	4
	*PGM (cytoplasmic) 2*	Sh01_t009050	Sh01:13231372..13239619 (+ strand)	6964	XM_002458121.2	12
	*PGM Bi-Functional 1*	Not found in monoploid genome	n/a	n/a	XM_021452820.1	6
	*PGM Bi-Functional 2*	Not found in monoploid genome	n/a	n/a	XM_002459927.2	2
**Sucrose Phosphate Phosphatase** (EC No. 3.1.3.24)	*SPP 1*	Not found in monoploid genome	n/a	n/a	XM_021459652.1	12
	*SPP 2*	Sh09_t003980	Sh09:6497383..6499722 (+ strand)	2340	XM_021447001.1	5
	*SPP 3*	Sh09_t003990	Sh09:6500269..6502971 (+ strand)	2703	XM_021447003.1	0
**Sucrose Phosphate Synthase** (EC No. 2.4.1.14)	*SPS 1*	Sh03_t030240	Sh03:49867902..49873435 (− strand)	5534	XM_002458946.2	12
	*SPS 2*	Sh04_t004940	Sh04:7514628..7521887 (+ strand)	7260	XM_021459119.1	2
	*SPS 3*	Sh10_t014400	Sh10:26819177..26833006 (− strand)	13,830	XM_021449299.1	2
	*SPS 4*	Sh_230M24_p000030	Not in STP only found in BAC sequences	30,575	XM_002441477.2	9
	*SPS 5*	Sh_254P01_p000060	Not in STP only found in BAC sequences	6027	XM_002449248.2	1
**Sucrose Synthase** (EC No. 2.4.1.13)	*SuSy 1–1*	Sh10_t006690	Sh10:10530379..10537554 (+ strand	7176	XM_021449494.1	34*
	*SuSy 1–2*	Sh10_t006710	Sh10:10605935..10613992 (− strand)	8058	XM_021449494.1	34*
	*SuSy 2*	Sh01_t026370 & Sh01_t026380	Sh01:44996485..45001411 (+ strand)	4816	XM_021456935.1	22
	*SuSy 4*	Sh01_t029560, Sh01_t029570 & Sh01_t029580	Sh01:49748737..49751357 (+ strand)	2600	XM_002465258.2	15
	*SuSy 6*	Sh04_t027720	Sh04:45363593..45367165 (+ strand)	3573	XM_021459722.1	0
	*SuSy 7*	Sh10_t019090 & Sh10_t019080	Sh10:34312395..34316604 (− strand)	4189	XM_021449504.1	3
**UDP Apiose/Xylose Synthase** (EC No. 4.1.1.35)	*UAXS*	Sh03_t033730	Sh03:54573981..54577001 (− strand)	3021	XM_002459126.2	3
**UDP-glucose 4,6-dehydratase (trifunctional)** (EC No. 4.2.1.76)	*RHM 1–1*	Sh01_t033530	Sh01:55740390..55744433 (− strand)	4044	XM_021450789.1	10
	*RHM 1–2*	Sh01_t033540	Sh01:55748951..55751423 (− strand)	2473	XM_021449119.1	12
	*RHM 3*	Sh09_t007030	Sh09:13224450..13225765 (− strand)	1316	XM_021447354.1	0
**UDP-glucose Dehydrogenase** (EC No. 1.1.1.22)	*UGD 2*	Sh01_t013840	Sh01:21392670..21394244 (+ strand)	1575	XM_021463640.1	0
	*UGD 4*	Sh01_t037600	Sh01:62131181..62134997 (− strand)	3817	XM_002468250.2	18
	*UGD 5*	Sh01_t005970	Sh01:8657598..8660482 (− strand)	2885	XM_021464912.1	23
**UDP-glucose Pyrophosphorylase** (EC No. 2.7.7.9)	*UGPase 1*	Sh10_t017220	Sh10:31655313..31671316 (− strand)	16,004	XM_021449324.1	1
	*UGPase 2*	Sh09_t013490	Sh09:26041479..26044800 (+ strand)	8212	XM_021447834.1	15
	*UGPase 3*	Not found in monoploid genome	n/a	n/a	XM_021447834.1	3
	*UGPase 4*	Not found in monoploid genome	n/a	n/a	XM_002453140.2	1
**UDP-glucose-4-Epimerase** (EC No. 5.1.3.2)	*GALE 1–1*	Not Annotated	Sh09:36286695..36291305 (+ strand)	4526	XM_002467816.2	19*
	*GALE 1–2*	Not Annotated	Sh01:49061275..49066223 (− strand)	4949	XM_002467816.2	19*
	*GALE 2*	Sh07_t008170	Sh07:13109984..13111148 (+ strand)	1165	XM_002445384.2	8
	*GALE 3*	Not Annotated	Sh02:34046443..34049056 (− strand)	2613	XM_021452554.1	0
	*GALE 4*	Not Annotated	Sh02:19730286..19736641 (+ strand)	6356	XM_002462165.2	1
**UDP-Glucuronic Acid Epimerase** (EC No. 5.1.3.12)	*UGE 1–1*	Not Annotated	Sh04:41812833..41814159 (− strand)	1327	XM_002452919.2	3
	*UGE 1–2*	Not Annotated	Sh10:9476275..9477597 (− strand)	1323	XM_002437940.2	0
	*UGE 6–1*	Not Annotated	Sh07:26068992..26070452 (+ strand)	1461	XM_002444670.2	0
	*UGE 6–2*	Not Annotated	Sh02:31857034..31858595 (− strand)	1562	XM_002462575.2	2
**UDP-Xylose Epimerase** (EC No. 5.1.3.5)	*UXE 1*	Not Annotated	Sh02:2941527..2944505 (+ strand)	2979	XM_002459288.2	3
	*UXE 2*	Not Annotated	Sh06:27777954..27782014 (− strand)	4061	XM_021463492.1	11
	*UXE 3*	Not Annotated	Sh01:53599712..53602144 (− strand)	5097	XM_002467958.2	2
**UDP-xylose synthase (UDP-glucuronic acid decarboxylase)** (EC No. 4.1.1.35)	*UXS 1–1*	Sh03_t025580	Sh03:43004627..43007178 (+ strand)	2552	XM_002456558.2	6
	*UXS 1–2*	Sh01_t033240	Sh01:55515466..55517229 (+ strand)	1764	XM_021464234.1	1
	*UXS 2–1*	Not Annotated	Sh01:49677978..49684559 (+ strand)	6682	XM_002465248.2	1
	*UXS 2–2*	Not found in monoploid genome	n/a	n/a	XM_002457713.2	1
	*UXS 4*	Sh09_t008330	Sh09:16796741..16798837 (− strand)	2097	XM_002440927.2	11
	*UXS 6*	Sh01_t033550	Sh01:55755363..55756710 (− strand)	7487	XM_021461997.1	7
**Hexokinase** (EC No. 2.7.1.11)	*HXK 2*	Sh03_t019610 & Sh03_t019620	Sh03:34702506..34705040 (+ strand)	2534	XM_021456830.1	0
	*HXK 3*	Not found in monoploid genome	n/a	n/a	XM_002459027.2	2
	*HXK 5*	Sh09_t015900	Sh09:29734562..29738896 (+ strand)	4335	XM_002440059.2	7
	*HXK 6*	Sh03_t020670	Sh03:36265525..36272095 (− strand)	6571	XM_002458422.2	1
	*HXK 7*	Sh09_t006090	Sh09:10277092..10280058 (+ strand)	2967	XM_002440690.2	2
	*HXK 8*	Sh03_t002520	Sh03:4682232..4685187 (+ strand)	2956	XM_021457110.1	3
	*HXK 10–1*	Sh09_t009120	Sh09:18357298..18360337 (− strand)	3040	XM_002440956.2	0
	*HXK 10–2*	Sh06_t013660	Sh06:24339191..24342711 (+ strand)	3521	XM_002440956.2	0

The No. of transcripts in SUGIT transcriptome column numbers with an asterisk "*" indicates shared transcripts as the sequences were too similar to tell apart. “Not in STP only found in BAC sequences” indicates the presence of a said gene in BAC sequences although it was not included in the STP sequence. EC no. refers to the Enzyme commission number obtained from the UniProt database

### Gene-specific and gene family specific expression profile throughout the sugarcane plant

#### Sucrose to UDP-glucose and UDP-glucose/hexose phosphate to sucrose associated gene families

**Fig. 2:**
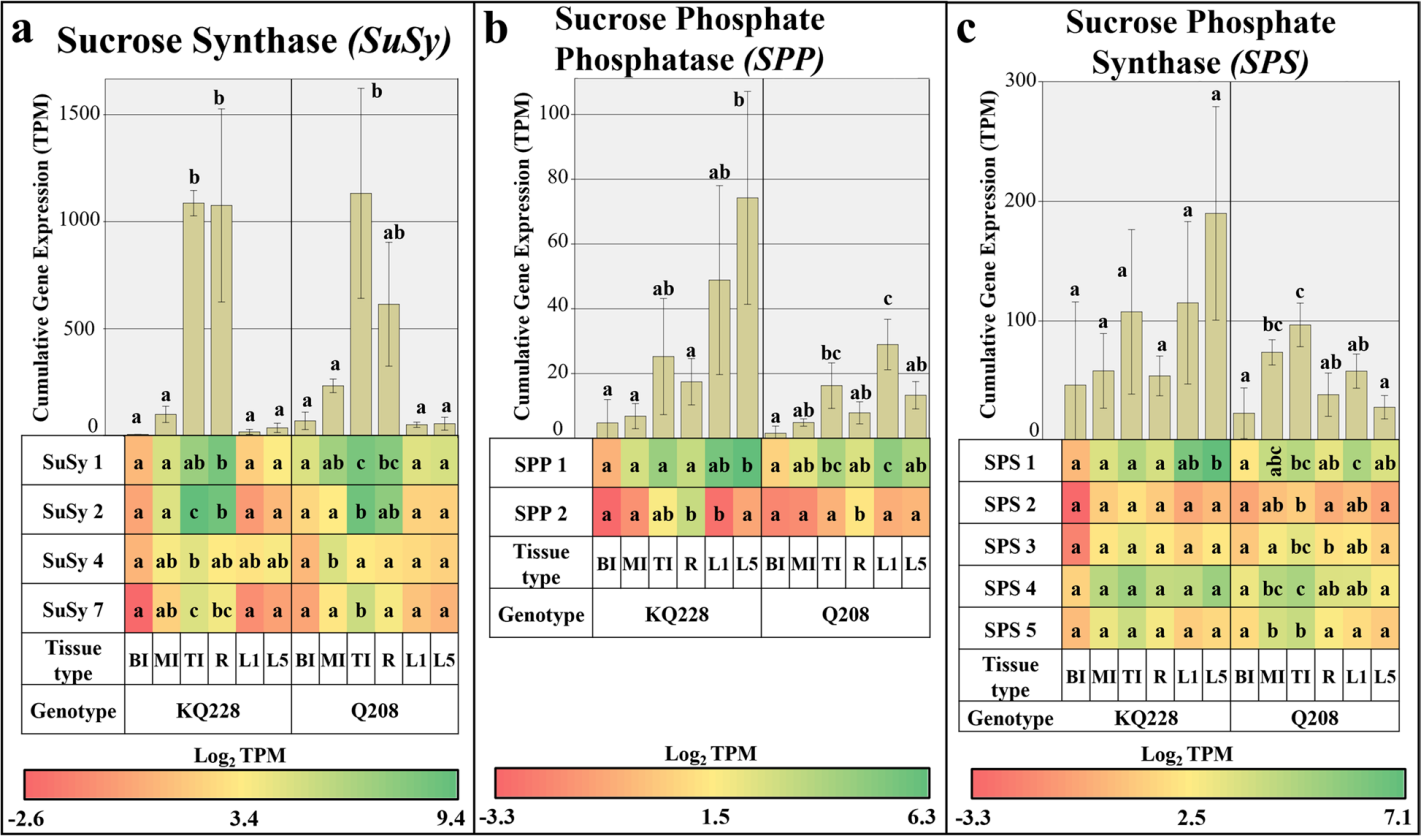
Cumulative and Isoform specific expression of gene families associated with the sucrose to UDP-Glucose, and UDP-Glucose to sucrose. Gene families: Sucrose Synthase (SuSy)in Figure **a**, Sucrose Phosphate Phosphatase (SPP) in Figure **b**, and Sucrose Phosphate Synthase (SPS) in Figure **c**. Abbreviations, TI: Top Internode; MI: Middle Internode; BI: Bottom Internode; 1st Visible Dewlap Leaf: L1; 5th Visible Dewlap Leaf: L5; R: Root. The bar chart displays the cumulative expression of all genes within a gene family. Letters above each bar indicate the presence of a significant difference between values within the same genotype. Error bars +/− 1 S.D. from biological triplicates. The heat map plots the Log_2_ TPM values for each individual gene family member. Green displays higher expression values, yellow for mid-range values and red for lower range expression values. Letters within the heatmap indicate the presence of a significant difference between values within an individual gene, within the same genotype. Significance was calculated via one way ANOVA, with the post-hoc Tukey’s T-test to separate statistically dissimilar groups. Statistical analysis was measured separately within each genotype

In the *SuSy* gene family, cumulative expression was significantly higher (*p ≤* 0.05) in R and TI compared to other organ samples of both Q208 and KQ228 genotypes (Fig. 2a). The 4 gene isoforms of *SuSy* displayed differing degrees of expression throughout the sugarcane plant. *SuSy 1, 2* and *7* had significantly higher expression in TI organs in comparison to leaf and mature internodal organs. No significant difference was observed between R and TI samples, however, differences between R and all other organs were not significant due to the high variance of triplicate values. *SuSy 1* and *2* were the most prominent gene isoforms in terms of expression.

In the sucrose synthetic *sucrose phosphate phosphatase (SPP)* gene family, cumulative expression was higher in KQ228 L5 and Q208 L1 leaf samples **(**Fig. 2b**)**. in comparison to the mature internodal samples, in both genotypes. The *SPP 2* isoform had significantly higher expression values in roots in comparison to leaf and mature internodal samples. The *SPP 1* isoform had heightened expression in L5 and L1 leaf samples in genotypes KQ228 and Q208, respectively. Among the two SPP isoforms, *SPP 1* had the highest volume of expression.

Cumulative *sucrose phosphate synthase (SPS)* gene expression was not significantly different throughout the KQ228 genotype, whereas a significantly higher degree of expression was observed in the Q208 genotype (Fig. 2c). In the KQ228 genotype, *SPS* gene isoform-specific expression did not differ between *SPS 2, 3, 4* and *5* genes, only *SPS 1* displayed significantly higher expression in L5 in comparison to root and internodal organs. Distinctly different isoform expression patterns were observed in the Q208 genotype with *SPS 2, 4* and *5* having significantly higher expression in TI sample in comparison to R, all leaf and BI samples. *SPS 1* expression was significantly higher in L1 sample in comparison to R, BI and L5 samples. The expression of *SPS 1* and *4* was the highest of all isoforms.

#### Cellulose synthase and cellulose synthase-like gene families

**Fig. 3:**
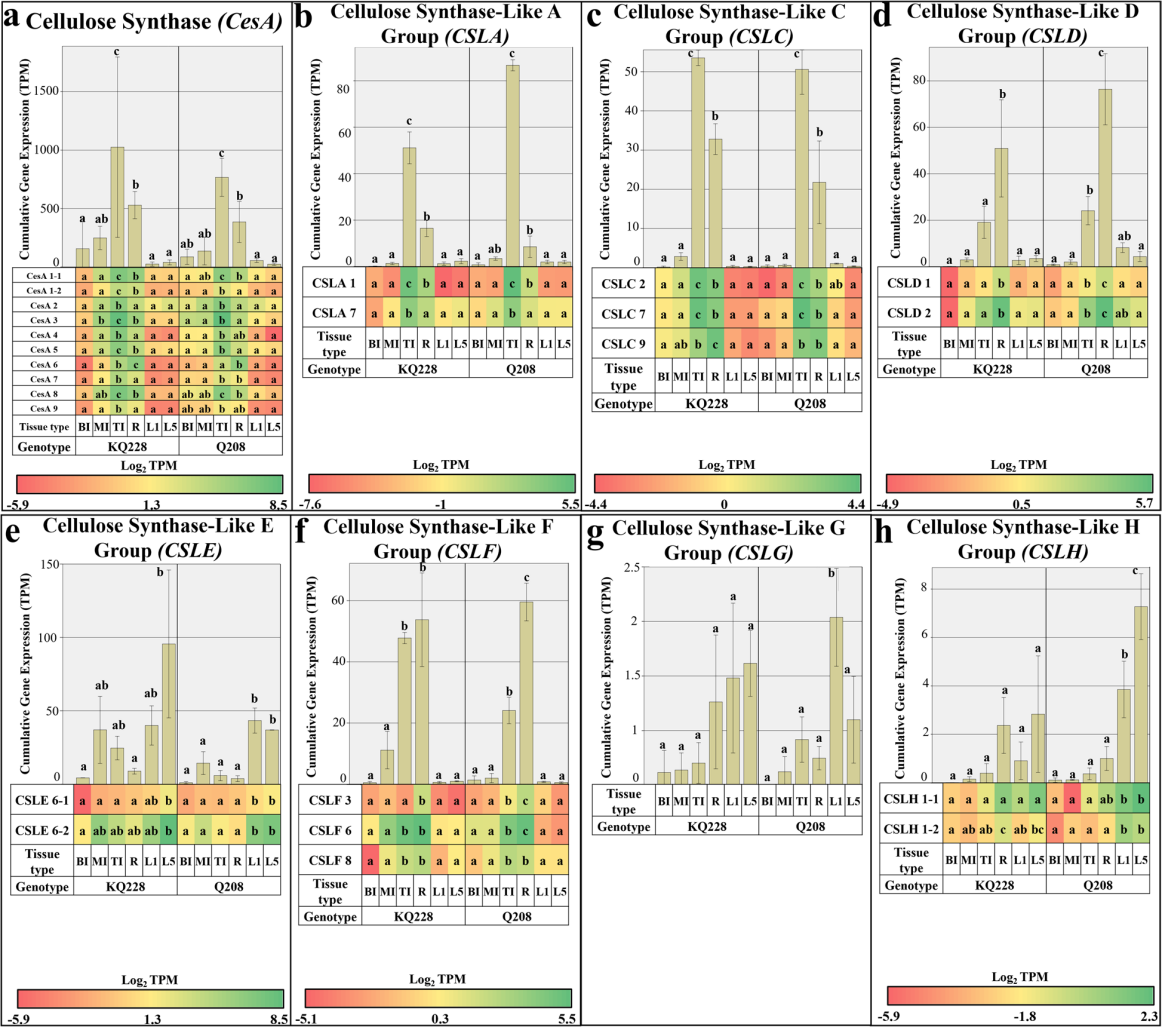
Cumulative and Isoform specific expression of gene families associated with the cellulose synthase (CesA) and cellulose synthase-like (CSL) gene families. Gene families: Cellulose Synthase (CesA) in Figure **a**, and Cellulose Synthase-Like (CSL) in Figures **b** to **h**. Abbreviations, TI: Top Internode; MI: Middle Internode; BI: Bottom Internode; 1st Visible Dewlap Leaf: L1; 5th Visible Dewlap Leaf: L5; R: Root. The bar chart displays the cumulative expression of all genes within a gene family. Letters above each bar indicate the presence of a significant difference between values within the same genotype. Error bars +/− 1 S.D. from biological triplicates. The heat map plots the Log_2_ TPM values for each individual gene family member. Green displays higher expression values, yellow for mid-range values and red for lower range expression values. Letters within the heatmap indicate the presence of a significant difference between values within an individual gene, within the same genotype. Significance was calculated via one way ANOVA, with the post-hoc Tukey’s T-test to separate statistically dissimilar groups. Statistical analysis was measured separately within each genotype

In general, comparing between two genotypes, cumulative expression of gene families related to cellulose biosynthesis including *CesA*, *CSLA*, *CSLC* and *CSLD* showed a significantly higher expression in TI and R samples compared to other samples used in this study (Fig. 3a, b, c and d, respectively). Cumulative expression of the *CesA* gene family was significantly higher in TI samples compared to other samples, while roots had significantly higher *CesA* expression than leaves. Isoform-specific expression for *CesA 1–1, 1–2, 2, 3, 5, 7* and *8* was also significantly higher in TI sample, whereas, *CesA 6* expression was significantly higher in root sample compared to other samples. The volume of *CesA 1–1, 3, 6* and *8* expression was the highest of all isoforms.

Cumulative expression of the *CSLA* gene family was significantly higher in TI samples, followed by R sample which had significantly higher expression than mature internode and leaf samples. Among the isoforms, *CSLA 1* and *7* expression was significantly higher in TI sample, and *CSLA 1* expression was significantly higher in R sample, in comparison to leaf and mature internodal samples. *CSLC* cumulative expression was significantly higher in TI sample than all other samples in both genotypes, followed by R sample which was significantly higher than mature internodal and leaf samples. Isoform-specific expression displayed enhanced expression of *CSLC 2* and *7* in TI samples, followed by R sample. In the KQ228 genotype, *CSLC 9* expression was significantly higher in R sample, whereas in Q208 there was no significant difference between TI and R sample, both of which had significantly higher expression values than the other samples.

Cumulative expression in the *CSLD* gene family was significantly higher in R samples. Isoform-specific expression of *CSLD 1* and *2* was significantly higher in root sample. *CSLD 1* expression was significantly higher than *CSLD 2* expression. The sum of *CSLE* expression was significantly higher in leaf samples (Fig. 3e). Isoform-specific expression of *CSLE 6–1* was significantly higher in leaf samples, whilst *CSLE 6–2* was significantly higher in leaf sample only in the Q208 genotype. *CSLE 6–2* had the most pronounced expression of the *CSLE* isoforms. Cumulative expression of the *CSLF* gene family was significantly higher in R and TI samples (Fig. 3f). Expression of the *CSLF 3* isoform was significantly higher in R samples, whilst expression for both *CSLF 6* and *8* was significantly higher in R and TI samples. *CSLF 6* had the most pronounced expression of the *CSLF* isoforms. Expression in the single *CSLG* gene was significantly higher in L1 sample in the Q208 genotype (Fig. 3g).

*CSLG* expression values tended to be higher in leaf samples, however due to the large variance in expression between replicates the differences were not significant, except L1 sample of Q208 genotype. Cumulative expression in the *CSLH* gene family did not differ between samples of the KQ228 genotype **(**Fig. 3h**)**, however, in the Q208 genotype expression was significantly higher in leaf samples. Isoform-specific expression of *CSLH 1–1* and *1–2* was significantly higher in leaf sample within the Q208 genotype. *CSLH 1–2* expression was significantly higher in root sample in comparison to internodal samples. *CSLH 1–1* had the most pronounced expression of the *CSLH* isoforms.

#### Hemicellulose synthesis associated gene families

**Fig. 4:**
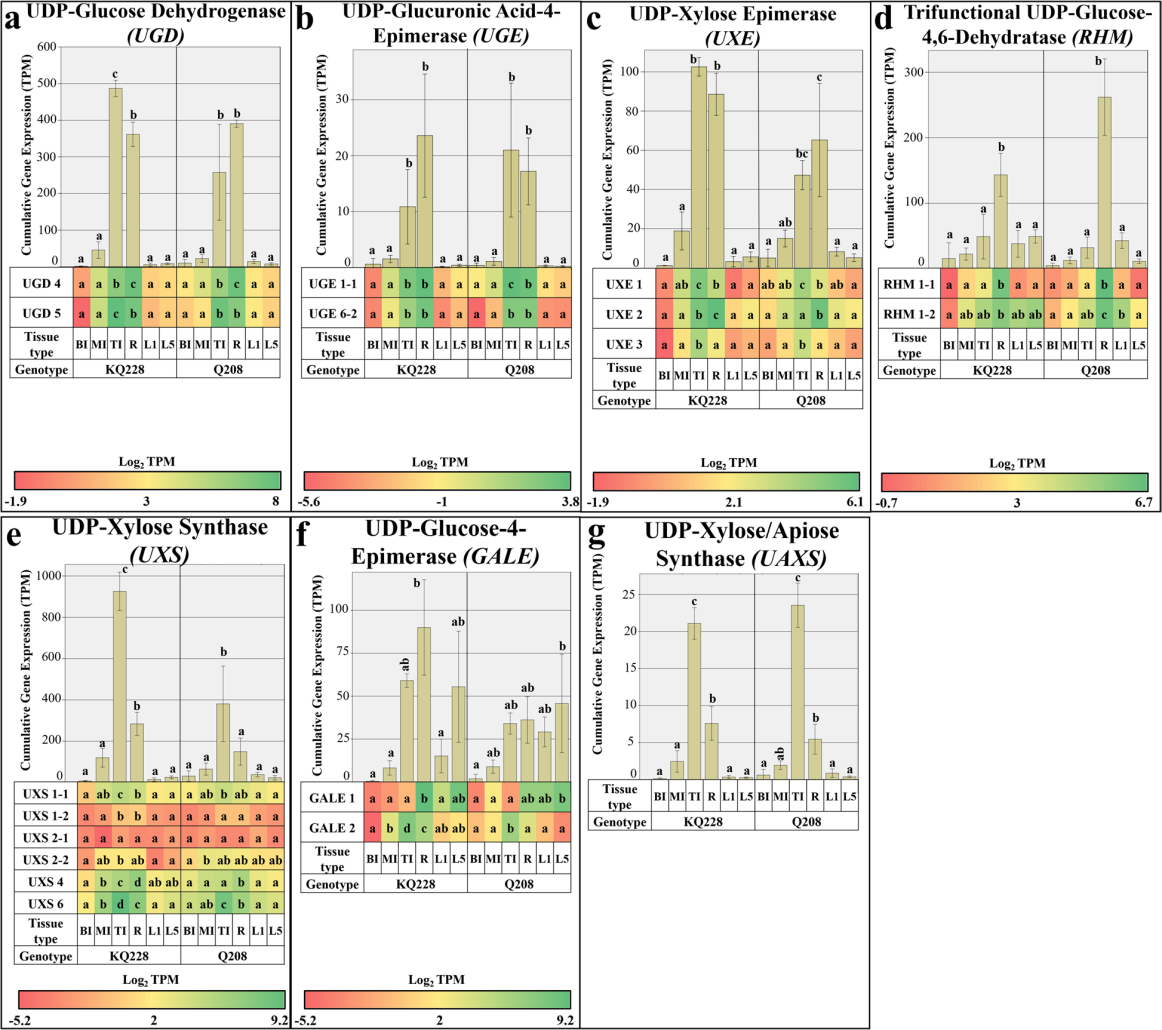
Cumulative and Isoform specific expression of gene families associated with hemicellulose synthesis. Gene families: Uridine Diphosphate Glucose Dehydrogenase (UGD) in Figure **a**, Uridine Diphosphate Glucuronic Acid Epimerase (UGE) in Figure **b**, Uridine Diphosphate Xylose Epimerase (UXE) in Figure **c**, Uridine Diphosphate Glucose 4,6-dehydratase (RHM) in Figure **d**, Uridine Diphosphate Xylose Synthase (UXS) in Figure **e**, Uridine Diphosphate Glucose 4-Epimerase (GALE) in Figure **f**, and Uridine Diphosphate Glucose Apiose/Xylose Synthase (UAXS) in Figure **g**. Abbreviations, TI: Top Internode; MI: Middle Internode; BI: Bottom Internode; 1st Visible Dewlap Leaf: L1; 5th Visible Dewlap Leaf: L5; R: Root. The bar chart displays the cumulative expression of all genes within a gene family. Letters above each bar indicate the presence of a significant difference between values within the same genotype. Error bars +/− 1 S.D. from biological triplicates. The heat map plots the Log_2_ TPM values for each individual gene family member. Green displays higher expression values, yellow for mid-range values and red for lower range expression values. Letters within the heatmap indicate the presence of a significant difference between values within an individual gene, within the same genotype. Significance was calculated via one way ANOVA, with the post-hoc Tukey’s T-test to separate statistically dissimilar groups. Statistical analysis was measured separately within each genotype

The UDP-G consuming UGD gene family displayed significantly higher cumulative expression rates in R and TI samples (Fig. 4a). Within the two *UGD* isoforms, *UGD 5* displayed significantly higher rates of expression in TI sample in comparison to all other samples. Root organ also had significant rates of *UGD 5* expression, which were significantly higher than leaf and mature internodal organs. *UGD 4* expression rates were significantly higher in R samples, closely followed by TI samples. Cumulative expression rates of the *UGE* gene family were significantly higher in R and TI samples across both genotypes. Isoform-specific expression of *UGE 1–1* and *UGE 6–2* was significantly higher in both R and TI samples **(**Fig. 4b**)**. *UGE 1–1* had the most pronounced expression of the *UGE* isoforms. Within the *UXE* gene family, cumulative expression was significantly higher in R and TI samples. Expression of the *UXE 1* and 3 isoforms was significantly higher in TI samples (Fig. 4c), whereas *UXE 2* had significantly higher expression in R samples, followed by TI sample. *UXE 2* had the most pronounced expression of the *UXE* isoforms.

Cumulative expression of the *RHM* gene family was significantly higher in root samples, in comparison to all other samples (Fig. 4d). Isoform-specific expression of *RHM 1–1* was significantly higher in root samples. Expression of *RHM 1–2* was not significantly different between all samples in the KQ228 genotype, whereas in the Q208 genotype expression was significantly higher in R samples. The *UXS* gene family had significantly higher expression in TI samples, closely followed by R samples which had significantly higher expression than mature internode and leaf samples **(**Fig. 4e**)**. Isoform-specific expression of the *UXS* gene family was as follows: *UXS 4* had significantly higher expression in the R samples, whereas *UXS 6* expression was significantly higher in TI samples. Expression rates of the other *UXS* isoforms tended to be higher in TI samples, although in some cases the differences were not significant between all samples. *UXS 6* had the most pronounced expression of the *UXS* isoforms. Cumulative expression of the *GALE* gene family did not show a clear expression pattern between samples (Fig. 4f), with all leaf, R and TI samples having pronounced expression, although expressional differences were not significant between all samples. Isoform-specific expression of *GALE 2* was significantly higher in TI sample, whereas *GALE 1* expression tended to be higher in R and both leaf samples, although the differences between these samples and the internodal samples were not consistent across both genotypes. The single gene with *UAXS* functionality had significantly higher expression in TI samples (Fig. 4g), followed by R sample which had significantly higher expression than leaf and mature internodal samples.

#### Myo-inositol pathway associated genes

**Fig. 5:**
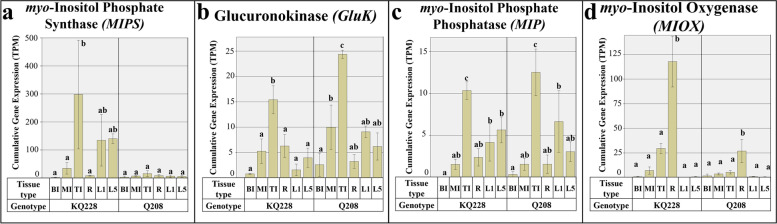
Expression of genes associated with the myo-Inositol pathway. Gene families: *myo-*Inositol Phosphate Synthase (MIPS) in Figure **a**, Glucuronokinase (GluK) in Figure **b**, *myo-*Inositol Phosphatase (MIP) in Figure **c**, and *myo-*Inositol Oxygenase (MIOX) in Figure **d**. Abbreviations, TI: Top Internode; MI: Middle Internode; BI: Bottom Internode; 1st Visible Dewlap Leaf: L1; 5th Visible Dewlap Leaf: L5; R: Root. The bar chart displays the cumulative expression of all genes within a gene family. Letters above each bar indicate the presence of a significant difference between values within the same genotype. Error bars +/− 1 S.D. from biological triplicates. The heat map plots the Log_2_ TPM values for each individual gene family member. Green displays higher expression values, yellow for mid-range values and red for lower range expression values. Letters within the heatmap indicate the presence of a significant difference between values within an individual gene, within the same genotype. Significance was calculated via one way ANOVA, with the post-hoc Tukey’s T-test to separate statistically dissimilar groups. Statistical analysis was measured separately within each genotype

Figure 5 displays the cumulative and gene isoform-specific expression values of genes associated with the *myo-*Inositol pathway. Only single genes were identified at each point in the *myo-*Inositol pathway. *MIPS* gene expression displayed distinctly different expression patterns between the two genotypes with larger rates of expression being observed in the KQ228 genotype (Fig. 5a). Whilst no significant differences were observed between samples in the Q208 genotype, within the KQ228 genotype, TI sample had significantly higher expression than R and mature internodal samples. *GluK* and *MIP* gene expression were significantly higher in TI samples in both genotypes (Fig. 5b and c). *MIOX* gene expression was significantly higher in root samples (Fig. 5d).

#### Invertase gene families

**Fig. 6:**
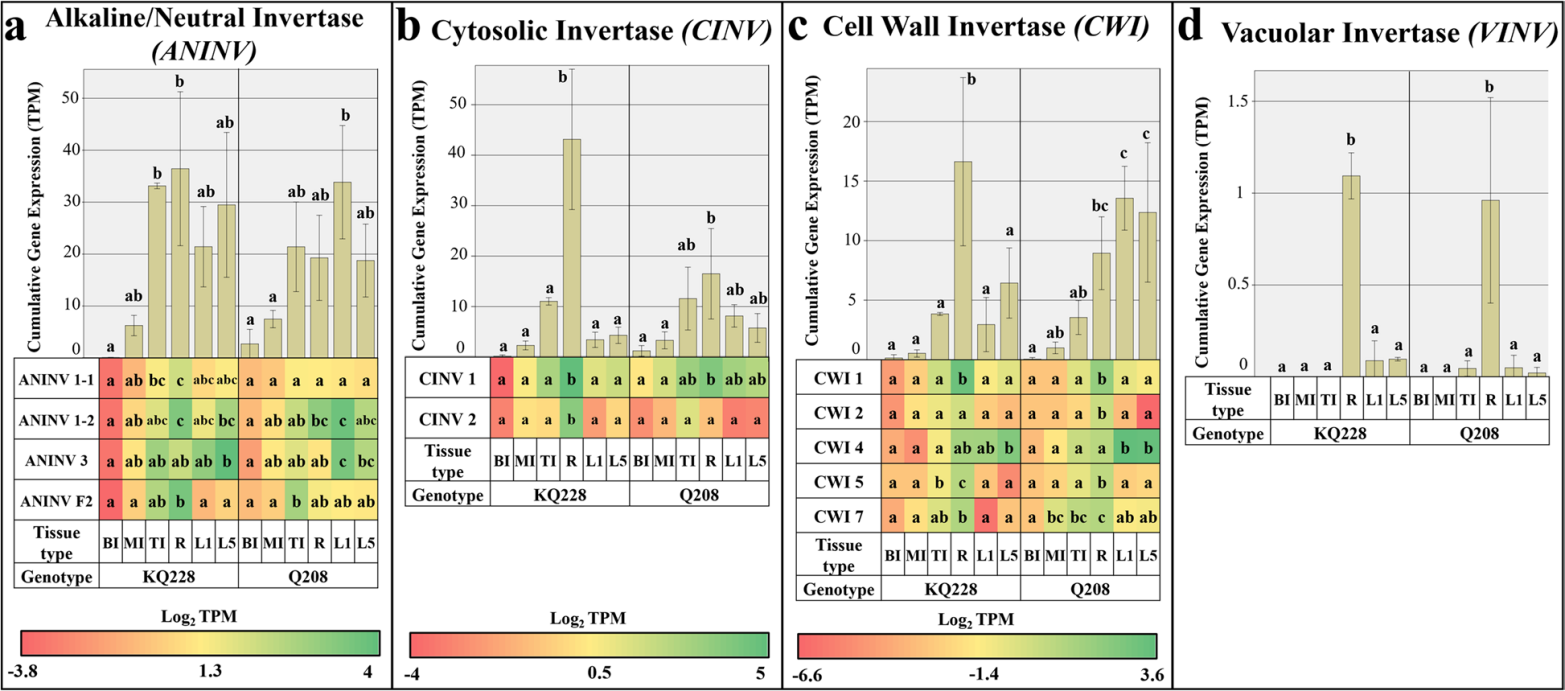
Cumulative and Isoform specific expression of gene families associated with Invertase activity. Gene families: Alkaline/Neutral Invertase (ANINV) in Figure **a**, Cytosolic Invertase (CINV) in Figure **b**, Cell Wall Invertase (CWI) in Figure **c**, and Vacuolar Invertase (VINV) in Figure **d**. Abbreviations, TI: Top Internode; MI: Middle Internode; BI: Bottom Internode; 1st Visible Dewlap Leaf: L1; 5th Visible Dewlap Leaf: L5; R: Root. The bar chart displays the cumulative expression of all genes within a gene family. Letters above each bar indicate the presence of a significant difference between values within the same genotype. Error bars +/− 1 S.D. from biological triplicates. The heat map plots the Log_2_ TPM values for each individual gene family member. Green displays higher expression values, yellow for mid-range values and red for lower range expression values. Letters within the heatmap indicate the presence of a significant difference between values within an individual gene, within the same genotype. Significance was calculated via one way ANOVA, with the post-hoc Tukey’s T-test to separate statistically dissimilar groups. Statistical analysis was measured separately within each genotype

Figure 6 displays the cumulative and gene isoform-specific expression values of invertase associated gene families. Within the *ANINV* gene family, cumulative and isoform-specific expression did not display clear expression patterns between the sample types (Fig. 6a). Cumulative expression patterns of the *CINV* gene family were higher in R samples in both genotypes, although differences were only significant in the KQ228 genotype (Fig. 6b). Isoform-specific expression of *CINV 1* and *2* was significantly higher in root sample in the KQ228 genotype, however, in the Q208 genotype these differences were not significant. Expression of the *CINV 1* isoform was higher than that of *CINV 2*. Cumulative expression of the CWI gene family was significantly higher in R samples in the KQ228 genotype (Fig. 6c), whereas R and both leaf samples had tended to have higher expression in the Q208 genotype, although only L5 sample had significantly higher expression than internodal samples. *CWI* isoforms *1, 2, 5* and *7* had significantly higher expression in R across both genotypes. *CWI 4* expression tended to be higher in leaf sample, with significantly higher expression being observed in the Q208 genotype. In the KQ228 genotype, only L5 sample had significantly higher expression than that of internodal samples. *CWI* isoforms *1* and *4* had the most pronounced expression of all *CWI* isoforms. *VINV* expression was significantly higher in roots in both genotypes (Fig. 6d).

#### Phosphorylated sugar synthesis /degradation associated gene families

**Fig. 7:**
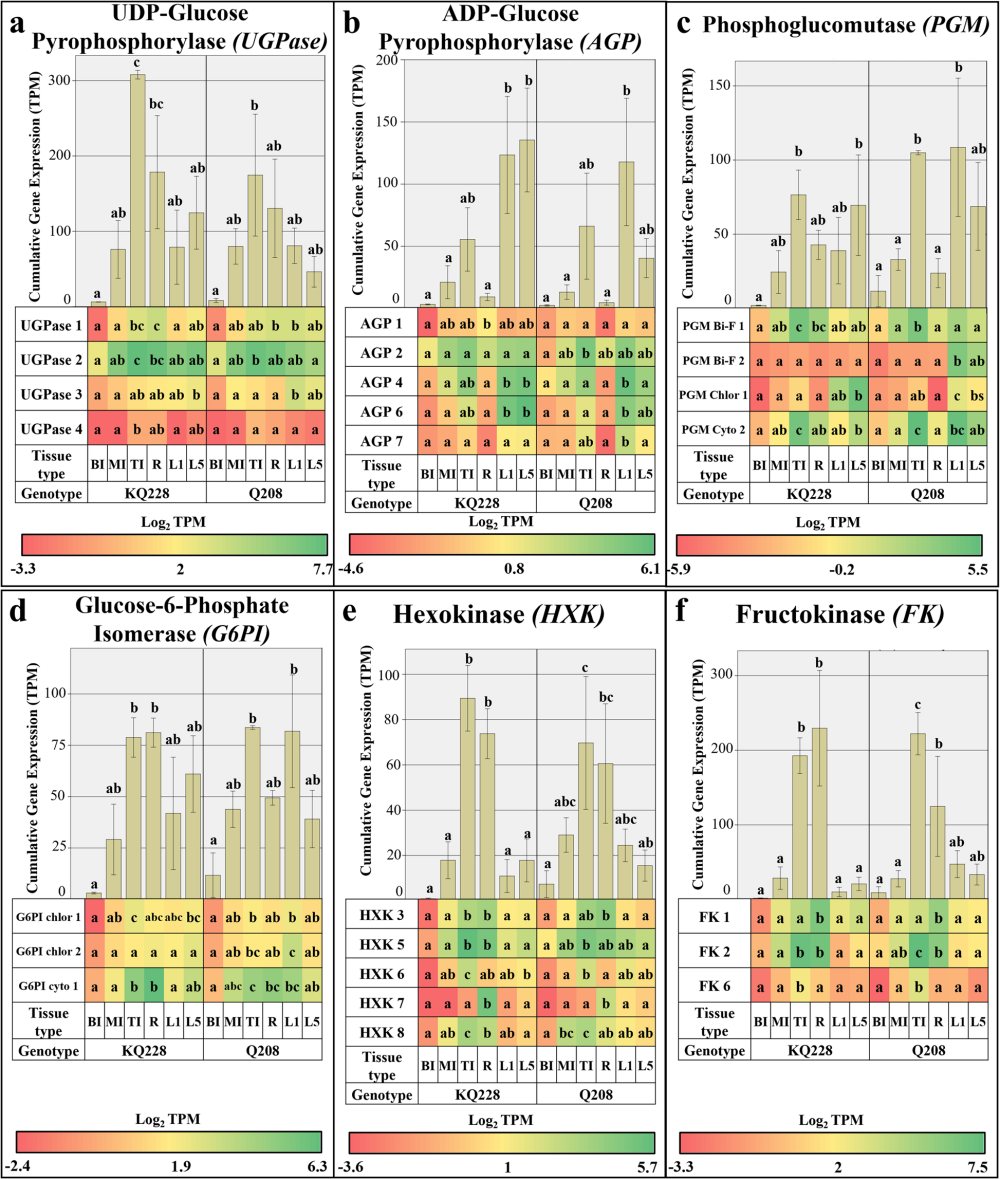
Cumulative and Isoform specific expression of gene families associated with Phosphorylated sugar synthesis and degradation. Gene families: Uridine Diphosphate Glucose Pyrophosphorylase (UGPase) in Figure **a**, Adenosine Diphosphate Glucose Pyrophosphorylase (AGP) in Figure **b**, Phosphoglucomutase (PGM) in Figure **c**, Glucose-6-Phosphate Isomerase (G6PI) in Figure **d**, Hexokinase (HXK) in Figure **e** and Fructokinase (FK) in Figure **f**. Abbreviations, TI: Top Internode; MI: Middle Internode; BI: Bottom Internode; 1st Visible Dewlap Leaf: L1; 5th Visible Dewlap Leaf: L5; R: Root. The bar chart displays the cumulative expression of all genes within a gene family. Letters above each bar indicate the presence of a significant difference between values within the same genotype. Error bars +/− 1 S.D. from biological triplicates. The heat map plots the Log_2_ TPM values for each individual gene family member. Green displays higher expression values, yellow for mid-range values and red for lower range expression values. Letters within the heatmap indicate the presence of a significant difference between values within an individual gene, within the same genotype. Significance was calculated via one way ANOVA, with the post-hoc Tukey’s T-test to separate statistically dissimilar groups. Statistical analysis was easured separately within each genotype

Figure 7 displays the cumulative and gene isoform-specific expression values of genes associated with the synthesis and degradation of phosphorylated sugars. Cumulative *UGPase* expression was significantly higher in TI samples in comparison to leaf and mature internodal samples in the KQ228 genotype (Fig. 7a). In the Q208 genotype whilst expression was observed to be higher in TI samples, this result was not significant. Isoform-specific expression of the *UGPase* gene family did not display any conclusive evidence of significant differences between samples in both genotypes. The *UGPase 2* isoform had pronounced expression in comparison to the 3 other *UGPase* isoforms. Cumulative *AGP* expression tended to be higher in leaf samples, closely followed by TI sample, however, this trend was only significant in the KQ228 genotype (Fig. 7b). Isoform-specific expression of *AGP 4* and *6* was significantly higher in leaf samples. Cumulative expression of the *PGM* gene family did not display any consistent expressional trends between samples in both genotypes (Fig. 7c). Isoforms *AGP 1, 2* and 6 had far more expression than other *AGP* isoforms. The chloroplastic *PGM 1* isoform had significantly higher expression in leaf sample, whilst bi-functional *PGM 1* and cytoplasmic *PGM 2* had significantly higher expression in TI sample. A significant amount of bi-functional *PGM 2* expression was observed in both L1 and L5 samples of the Q208 genotype, however, this trend was not observed in the KQ228 genotype.

*G6PI* cumulative and isoform-specific expression did not display any consistently significant expressional trends between samples across both genotypes (Fig. 7d). Cumulative expression of the *FK* gene family was significantly higher in TI and R samples across both genotypes (Fig. 7f). Expression of the *FK 1* isoform was significantly higher in R sample, whereas expression for *FK 2* and *6* was significantly higher in TI sample. *FK 1* and *2* had pronounced expressional values in comparison to the *FK 3* isoform. Cumulative expression in the *HXK* gene family was significantly higher in TI and R samples (Fig. 7e). Expression of *HXK 6* and 8 isoforms was significantly higher in TI samples across both genotypes. There was a significantly higher expression of *HXK 7* isoform in R samples. *HXK 3* expression was significantly higher in both R and TI samples.

## Discussion

The importance of UDP-G control and its effect on C partitioning has been indicated in several studies of plant systems, whereby the kinetic properties and abundance of enzymes involved in UDP-G synthesis/utilisation had a significant effect upon the volume of C moving into the hemicellulose, cellulose and sucrose pools [13–19]. UDP-G metabolism is central to key sources of C deposition in the sugarcane plant, therefore is likely that the expression of genes involved in UDP-G metabolism would have differed expression in relation to how C is utilised in a given tissue/organ. Additionally, most of these genes are known to be in multi-gene families, and many of these multi-gene families likely contain isoforms that overall have a larger effect on metabolism than others. By determining tissue-specific isoforms, gene candidates for altering UDP-G metabolism and by proxy C metabolism could be identified, in order to define the strategies to modify plant biomass. Here, to provide a broad overview of differences in organ-specific expression of genes associated with the UDP-G metabolism, we analysed their expression in 3 major organs of the sugarcane plant including leaves (mature source organ), internodes (young and mature sink organ) and root (meristematic sink organ).

### Expression of genes associated with the UDP-G metabolism in internodes

Sucrose synthesis and degradation in internodal samples by the SPP, SPS and SuSy enzymes (Fig. 2), is closely linked to sugarcane internodal maturity, whereby sucrose cleavage and hydrolysis prevails in immature internodes and sucrose synthesis and lack of sucrose utilization into respiration and insoluble components prevails in mature internodes [47]. As indicated in this study and previous studies of *SuSy* genes, expression and enzymatic activity are significantly higher in immature internodes [47, 48], likely providing C for hemicellulose and cellulose synthesis from UDP-G. Interestingly, within the *SuSy* gene family, the highest expression was limited to 2 of the 4 *SuSy* genes (*SuSy 1* and *2*) indicating that they may code for enzymes that have heightened importance in the cleavage of sucrose into UDP-G (Fig. 2a), as has been reported in Arabidopsis and Sugarcane [30, 48, 49]. As expected, enhanced expression of *SuSy* in immature internodes, also coincided with higher expression in cell wall synthetic related gene families, including *CesA*, *CSL* (A, C, D and F families, see Fig. 3), *GALE*, *UAXS*, *UGE*, *UXE* and *UGD* (Fig. 4). Some of the aforementioned genes code for enzymes that directly consume UDP-G, including *UGD*, *CesA* and some *CSL* gene families [50–52], in essence having direct competition with sucrose synthetic enzymes. The key genes coding for enzymes that are responsible for the bulk of UDP-G into cellulose and hemicellulose pools are likely coded for by *UGD 5* (Fig. 4a), and *CesA* subunit genes 1–1, 1–2, 3, 5, 7 and 8 (Fig. 2a). In a related study in the UGD gene family in *Arabidopsis,* an enzyme isoform with high affinity for UDP-Glucose, also had high affinity for the downstream product UDP-xylose, which acts as a feedback inhibitor [21], The associated genes also had higher expression, suggesting they code for enzymes that hold a primary role in the assimilation of C into the hemicellulose pool in immature tissues. Other UGD gene isoforms with lower volumes of expression likely code for enzymes with low substrate affinity and operate in the background throughout the sugarcane plants lifecycle. This concept could credibly be applied to most gene families in this study.

In conjunction with the heightened sucrose cleavage to UDP-G, cumulative *invertase* expression in *CINV* and *CWI* gene families was significantly higher in immature internodes in comparison to mature internode samples (Fig. 4b and c, respectively). This was expected as sucrose hydrolysis into reducing sugars characterizes the first step of C movement into several pathways, leading into cell wall, protein, respiratory and other secondary metabolite pools [53]. The heightened levels of reducing sugars in immature internodes [32], indicates the activity of invertases hydrolysing imported sucrose. The heightened expression of the *CWI 1* and *7* genes in the immature internodes (Fig. 4c), suggests prominent activity of C fixation into parenchyma tissue from conducting tissues, and the apoplastic/symplastic transfer of sucrose [54, 55]. Lower expression of *CWI* in mature internodes may suggest that C importation facilitated by the activity of invertases is less pronounced. The heightened expression of genes encoding phosphorylating enzymes, *FK* and *HXK* in immature internodes (Fig. 7e and f), particularly genes *HX 3* and *5* and *FK 2*, further supports the notion of high C movement likely toward pentose phosphate pathway and glycolysis, as reviewed by [56]. The indication of enhanced C flux into these pathways that may be mediated by gene expression is important, as results from [39] suggested very little difference in expression of transcripts associated with these pathways, which may suggest associated enzymes operate during favourable metabolic conditions, i.e. when metabolites are available. This indicates the potential importance of invertases and phosphorylating enzymes in releasing C to the pentose phosphate and glycolytic pathways.

Unexpectedly, in the sucrose synthetic gene families, *SPS* and *SPP*, between the mature and immature internodes, expression was higher in immature internodes (Fig. 2b and c), which is in contradiction to several studies that have found an opposite trend in enzymatic activity of SPS [47, 57]. It must be noted that enzymatic activity and gene expression are not necessarily correlated [58]. Further, another study has found similar results, whereby enzymatic activity was higher in immature internodes. The results from this study may indicate that the absence of competition for C in mature internodes by sucrose degradative and downstream enzymes, is the key to sucrose accumulation in mature internodes, as has been indicated in several studies [47, 59]. In a previous study by Botha and Black (2000), it was intimated that there could be an additional kinetic form of SPS that allows for heightened enzymatic activity, which explains the heightened activity of SPS in mature internodes [47], however, there is no evidence of increased transcription in any of the 5 *SPS* isoforms in mature internodes in our study. These results suggest enzymatic activity of SPS is determined beyond transcription.

### Expression of genes associated with the UDP-G metabolism in roots

Root and immature internodes are both meristematic sinks, moving a large proportion of fixed C into the cell wall, protein and respiratory pools. Despite this, there were large expressional differences between root and immature internode samples likely indicating spatial regulation of specific gene family isoforms. Some of the transcriptional differences between the two meristematic sinks is likely a result of the difference in age the samples were taken from the sugarcane plant (roots from a 3-month-old plant and immature internodes from a 9-month-old plant). However, previous compositional analyses of sugarcane roots display a differing requirement for fixed C than immature internodes [32, 60]. Compositional analyses of 3-month old sugarcane roots indicated differences in simple sugar and hemicellulosic monomer content in comparison to immature internodes [32]. Also, in immature internodes, there is an underlying trend for the accumulation of sucrose that does not exist in roots [60]. Concerning the simple sugar content, i.e. fructose, glucose and sucrose, it was postulated that the reduced levels in the root sample indicate the efficient breakdown and movement of fixed C into the cell wall, protein, organic acid and respiratory pools in roots. This is made clear by the high expression of *VINV* in roots (Fig. 4d), which has been implicated as a key enzyme negatively affecting the accumulation of sucrose in several plant systems, as reviewed by [61]; and the heightened expression of *SPS 1* and *4* genes (only significant in Q208 genotype, see Fig. 2c). In this study, low *VINV* expression in all internodes and leaf samples, indicating low VINV activity (Fig. 4d), likely allows the accumulation of sucrose for storage or transportation. Higher observed expression of *VINV* in roots suggests a lower requirement for sucrose bioaccumulation. Additionally, the high expression of two other invertase families in roots in comparison to immature internodes, including, *CINV* and *CWI* may also likely indicate a low inclination for sucrose bioaccumulation in roots (Fig. 4b and c). The higher expression of *CWI i*soforms *1, 2, 5* and *7* in root samples in comparison to immature internodes suggest a heightened role for CWI in roots, may enable an increase in hydrolysis of apoplastic sucrose, which in turn ensures a steep concentration gradient enhancing sucrose delivery to roots from mature leaves [62]. Interestingly, in sugarcane, the activity of *CWI* in internodes is correlated with higher sucrose levels [63, 64]. Enhanced invertase activity is counterintuitive to enhanced sucrose levels, however, the sucrose cleavage and resynthesis model as proposed by Glasziou and Gayler [65], may explain this. Higher expression of *CWI* genes in roots suggests the intercellular sucrose cleavage and intracellular resynthesis model to not be relevant in the root sample, as there is no evidence for large degrees of sucrose resynthesis to be occurring [32]. This notion is further supported by the lower expression of *SPS* observed in roots (only significantly different in Q208 genotype, Fig. 2c) in comparison to immature internodes and leaf samples.

Corresponding expression of *SuSy* genes between immature internodes and root samples likely indicates a high degree of UDP-G formulation from sucrose (Fig. 2a), followed by C utilisation into cellulose and hemicellulose pools. The expression of genes associated with UDP-G into cell wall polysaccharides differed greatly between immature internodes and root samples. Heightened expression of an additional *UGD* gene family isoform (*UGD 4*), and the *MIOX* gene suggests there may be enhanced enzymatic activity indicating the strong demand of C to be moved into the hemicellulose fraction in roots or an organ specific function (Figs. 4a and 5d, respectively). Evidence of heightened expression in a specific UGD isoform has been reported in *Arabidopsis* seedlings [21]. Additionally, downstream steps of hemicellulose synthesis displayed a significant difference between these two samples, which could be responsible for the heightened amount of arabinose and galactose mixed linkages in roots [32], particularly *GALE 1* and *UXE 2* (Fig. 4f and c). Although, as shown in related analyses, there was also higher expression in other hemicellulose related transcripts in roots, that do not result in differences in composition [32, 39]. Interestingly, *CesA* expression was significantly higher in immature internodes (Fig. 3a). The significant differences in gene expression related to hemicellulose and cellulose synthesis could be related to the presence of specialised cells in both roots and internodes [66–68], having different requirements for C, or differing metabolic conditions, i.e. access to substrates or the presence of feedback inhibitory molecules [21]. Notably, compositional analysis of roots and internodes, as presented in [32], displayed no difference in the ratio of hemicellulose and cellulose, indicating differences in expression of related genes may not affect the fixed nature of the cell wall component ratios.

### Expression of genes associated with the UDP-G metabolism in leaves

The expression of genes related to the UDP-G metabolism in sugarcane leaves is connected to the status of this organ as a net exporter of C in the form of sucrose. As expected, cell wall-related genes that directly synthesize or consume UDP-G, including *SuSy*, *CesA*, *CSL* and *UGD* gene families, had insignificant amounts of expression in the leaf samples, indicating a transcriptional regulation as a means of ceasing C flow into the cell wall pool in this organ. Interestingly, some *CSL* gene family groups (Fig. 3) had significant expression in leaves including *CSLE*, *CSLG* and *CSLH*, specifically *CSL6–2*, *CSLG2* and *CSLH1–1* genes (Fig. 3e, g and h, respectively). CSL enzymes are responsible for the transfer of UDP-G to 1–3 and 1–4 β-glucan, or the transfer of other nucleotide sugars to form other β-linked backbones, within the hemicellulose fraction. Most *CSL* genes had significantly higher expression in both meristematic/immature sink samples, which was expected due to the requirement for hemicellulose synthesis. It is unclear why there was significant expression of some *CSL* gene family groups in leaf samples. Of the *CSL* gene groups with significant expression in leaves, *CSLE* and *CSLG* have an unknown function (Fig. 3e and g), as reviewed by [69], although it is likely still associated with hemicellulose synthesis, whereas the *CSLH* group (Fig. 3h) encodes mixed linkage glucan synthases [70]. These CSL groups may have a heightened requirement in leaf sample during development constructing leaf specific structures, with different arrangements of cell wall compounds. The data would also suggest that expression of these genes are retained throughout maturity exclusively in leaf samples, and may also have a role in maintenance.

Unlike most cell wall-related gene expression, high expression of sucrose synthetic genes *SPS* and *SPP* (Fig. 2b and c), equivalent to both meristematic samples were observed in leaf sample. Expression of *SPP 1* and *SPS 1* was most prominent in the leaf (although not significantly different from other samples), which may suggest these specific genes have an enhanced role in leaf sample. This trend has been hypothesised to be due to pronounced role of SPS and SPP in the synthesis of sucrose in source tissues [71, 72]. Relevant to sucrose biosynthesis in leaves is the production of UDP-G which is likely primarily derived from the activity of UGPase which transfers glucose-1-phosphate to UDP-G, whereas in sink samples UDP-G synthesis is derived primarily from sucrose cleavage by SuSy [73]. In support of this notion, transcription of SuSy related genes (Fig. 2a) was significantly higher in meristematic sinks. However, despite the primary the role of UGPase in sucrose biosynthesis in leaves, there was not higher expression in related gene family isoforms (Fig. 7a). This suggests the pronounced role of UGPase in source samples is not determined at the transcriptional level, but at the metabolic level, likely via the availability of hexose phosphates [74]. As indicated by the low expression of *SuSy* and high expression of *SPS* and *SPP* in leaves (Fig. 2c and b), this suggests a bias toward sucrose synthesis in this source organ, which will then be transported to various sink organs. However, the high expression of some *invertase* genes in the *CWI (CWI 4)*, and *ANINV (ANINV 1–1* and *3)* gene families suggest sucrose hydrolysis to be a major competing sink for C (Fig. 6c and a, respectively). In a previous experiment of photosynthetic regulation by sugars in sugarcane leaves, fed radiolabelled sucrose was rapidly converted into hexoses, which was stipulated to be due to the activity of SuSy and invertase enzymes [75]. However, based on the low expression of *SuSy* genes in source organs in this study, the rapid conversion of sucrose may be mostly derived from invertase activity in leaves. The heightened activity of some invertases in sugarcane leaves suggests a major role in the regulation of sugar levels, especially due to the inhibitory nature of high sucrose concentrations on photosynthetic activity [76, 77]. In the case of the *CWI* gene family, most expression in leaves was contributed by the *CWI 4* gene (Fig. 6c), which was also significantly higher than other organ types, which suggests an organ-specific function for this gene, potentially as a regulator of sucrose levels. It must be noted that as leaf organ is highly metabolically active and is a protein-rich organ [32], there is likely still an underlying requirement for C to be moved into respiratory and protein fractions. It is possible that invertase activity is contributing C to these pools.

## Conclusions

This study represents the first effort to quantify the expression of gene families associated with UDP-G metabolism in a sugarcane plants. The data presented provides a quality reference for future efforts in altering UDP-G metabolism and in turn carbon partitioning in sugarcane. Transcriptional analysis displayed the likelihood that carbon partitioning in sugarcane is closely related to the transcription of genes associated with the UDP-G metabolism in the four major modes of carbon partitioning as defined in leaves (source), roots (non-storage immature/meristematic sink), mature internodes (storage sink) and immature internodes (immature sink that will become a storage sink). The data presented may provide an accurate genetic reference for future efforts in altering UDP-G metabolism and in turn C partitioning in sugarcane.

## Supplementary Information

**Additional file 1: Table S1.** Variety factsheet for Q208 and KQ228 from QCANESelect™ variety selection webpage. **Fig. S1.** Schematic of sugarcane transcript gene family homologue assignment and quantification. **Table S2.** Numerical Identifier for Tukey *t*-test results and one-way ANOVA significance values. **Fig. S2.** One-way ANOVA and Tukey *t*-test results from SuSy gene family expression comparisons. **Fig. S3.** One-way ANOVA and Tukey *t*-test results from SPS gene family expression comparisons. **Fig. S4.** One-way ANOVA and Tukey *t*-test results from SPP gene family expression comparisons. **Fig. S5.** One-way ANOVA and Tukey *t*-test results from CSLA gene family expression comparisons. **Fig. S6.** One-way ANOVA and Tukey *t*-test results from CesA gene family expression comparisons. **Fig. S7.** One-way ANOVA and Tukey *t*-test results from CSLC gene family expression comparisons. **Fig. S8.** One-way ANOVA and Tukey *t*-test results from CSLD gene family expression comparisons. **Fig. S9.** One-way ANOVA and Tukey *t*-test results from CSLE gene family expression comparisons. **Fig. S10.** One-way ANOVA and Tukey *t*-test results from CSLF gene family expression comparisons. **Fig. S11.** One-way ANOVA and Tukey *t*-test results from CSLG gene family expression comparisons. **Fig. S12.** One-way ANOVA and Tukey *t*-test results from CSLH gene family expression comparisons. **Fig. S13.** One-way ANOVA and Tukey *t*-test results from UGD gene family expression comparisons. **Fig. S14.** One-way ANOVA and Tukey *t*-test results from UGE gene family expression comparisons. **Fig. S15.** One-way ANOVA and Tukey *t*-test results from UXE gene family expression comparisons. **Fig. S16.** One-way ANOVA and Tukey *t*-test results from RHM gene family expression comparisons. **Fig. S17.** One-way ANOVA and Tukey *t*-test results from GALE gene family expression comparisons. **Fig. S18.** One-way ANOVA and Tukey *t*-test results from UXS gene family expression comparisons. **Fig. S19.** One-way ANOVA and Tukey *t*-test results from UAXS gene family expression comparisons. **Fig. S20.** One-way ANOVA and Tukey *t*-test results from MIPS gene family expression comparisons. **Fig. S21.** One-way ANOVA and Tukey *t*-test results from GluK gene family expression comparisons. **Fig. S22.** One-way ANOVA and Tukey *t*-test results from MIP gene family expression comparisons. **Fig. S23.** One-way ANOVA and Tukey *t*-test results from MIOX gene family expression comparisons. **Fig. S24.** One-way ANOVA and Tukey *t*-test results from Invertase gene family ANINV. **Fig. S25.** One-way ANOVA and Tukey *t*-test results from Invertase gene family CINV. **Fig. S26.** One-way ANOVA and Tukey *t*-test results from Invertase gene family CWI. **Fig. S27.** One-way ANOVA and Tukey *t*-test results from Invertase gene family VINV. **Fig. S28.** One-way ANOVA and Tukey *t*-test results from UGPase gene family expression comparisons. **Fig. S29.** One-way ANOVA and Tukey *t*-test results from AGP gene family expression comparisons. **Fig. S30.** One-way ANOVA and Tukey *t*-test results from PGM gene family expression comparisons. **Fig. S31.** One-way ANOVA and Tukey *t*-test results from G6PI gene family expression comparisons. **Fig. S32.** One-way ANOVA and Tukey *t*-test results from HXK gene family expression comparisons. **Fig. S33.** One-way ANOVA and Tukey *t*-test results from FK gene family expression comparisons.

## Data Availability

The version of the SUGIT database used in this study can be accessed in Figshare under the 10.6084/m9.figshare.4981655. The RNA-seq data used for expression profiling has been deposited in the NCBI Sequence Read Archive (SRA) under the BioProject ID PRJNA479814, Study Accession Number SRP152893.

## Abbreviations

C: Carbon
NGS: Next Generation Sequencing
mRNA: Messenger RNA
cDNA: Complementary DNA
BI: Bottom Internode
MI: Middle Internode
TI: Top Internode
L1: 1st visible dewlap leaf
L5: 5th visible dewlap leaf
R: Root
UDP-G: Uridine Diphosphate Glucose
CWI: Cell Wall Invertase
VINV: Vacuolar Invertase
CINV: Cytosolic Invertase
ANINV: Alkaline/Neutral Invertase
FK: Fructokinase
MIPS: *myo-*Inositol phosphate synthase
MIP: *myo-*Inositol phosphatase
MIOX: *myo-*Inositol oxygenase
GluK: Glucuronokinase
PGM: Phosphoglucomutase
UGPase: Uridine Diphosphate Glucose pyrophosphorylase
UGD: UDP-Glucose Dehydrogenase
AGP: Adenosine Diphosphate Glucose pyrophosphorylase
CesA: Cellulose Synthase
CSL: Cellulose Synthase-Like
G6PI: Glucose-6-Phosphate Isomerase
SPP: Sucrose Phosphate Phosphatase
SPS: Sucrose Phosphate Synthase
SuSy: Sucrose Synthase
RHM: Uridine Diphosphate Glucose 4,6-dehydratase
UAXS: Uridine Diphosphate Glucose Apiose/Xylose Synthase
GALE: Uridine Diphosphate Glucose 4-Epimerase
UGE: Uridine Diphosphate Glucuronic Acid Epimerase
UXS: Uridine Diphosphate Xylose Synthase
UXE: Uridine Diphosphate Xylose Epimerase
HXK: Hexokinase
