# Supplementary material for: Organ-specific expression of genes associated with the UDP-glucose metabolism in sugarcane (Saccharum spp. hybrids)
Source: BMC Genomics. 2023 Jan 13;24:18. doi: 10.1186/s12864-023-09124-8 (PMC9840354; doi:10.1186/s12864-023-09124-8)
Supplement: Supplementary file 1 — Additional file 1: Table S1. Variety factsheet for Q208 and KQ228 from QCANESelect™ variety selection webpage. Fig. S1. Schematic of sugarcane transcript gene family homologue assignment and quantification. Table S2. Numerical Identifier for Tukey t-test results and one-way ANOVA significance values. Fig. S2. One-way ANOVA and Tukey t-test results from SuSy gene family expression comparisons. Fig. S3. One-way ANOVA and Tukey t-test results from SPS gene family expression comparisons. Fig. S4. One-way ANOVA and Tukey t-test results from SPP gene family expression comparisons. Fig. S5. One-way ANOVA and Tukey t-test results from CSLA gene family expression comparisons. Fig. S6. One-way ANOVA and Tukey t-test results from CesA gene family expression comparisons. Fig. S7. One-way ANOVA and Tukey t-test results from CSLC gene family expression comparisons. Fig. S8. One-way ANOVA and Tukey t-test results from CSLD gene family expression comparisons. Fig. S9. One-way ANOVA and Tukey t-test results from CSLE gene family expression comparisons. Fig. S10. One-way ANOVA and Tukey t-test results from CSLF gene family expression comparisons. Fig. S11. One-way ANOVA and Tukey t-test results from CSLG gene family expression comparisons. Fig. S12. One-way ANOVA and Tukey t-test results from CSLH gene family expression comparisons. Fig. S13. One-way ANOVA and Tukey t-test results from UGD gene family expression comparisons. Fig. S14. One-way ANOVA and Tukey t-test results from UGE gene family expression comparisons. Fig. S15. One-way ANOVA and Tukey t-test results from UXE gene family expression comparisons. Fig. S16. One-way ANOVA and Tukey t-test results from RHM gene family expression comparisons. Fig. S17. One-way ANOVA and Tukey t-test results from GALE gene family expression comparisons. Fig. S18. One-way ANOVA and Tukey t-test results from UXS gene family expression comparisons. Fig. S19. One-way ANOVA and Tukey t-test results from UAXS gene family expression comparisons. Fig [file 12864_2023_9124_MOESM1_ESM.docx]

**SUPPLEMENTARY INFORMATION**

**Organ-specific expression of genes associated with the
UDP-glucose metabolism in sugarcane (*Saccharum* spp. hybrids)**

Patrick J Mason, Nam V Hoang, Frederik C Botha, Agnelo Furtado,
Annelie Marquardt, Robert J Henry.

**Figures S1-S33, and Table S1-S2**

- **Table S1.** Variety factsheet for Q208 and KQ228 from QCANESelect™ variety selection webpage.
- **Figure S1.** Schematic of sugarcane transcript gene family homologue assignment and quantification.
- **Table S2.** Numerical Identifier for Tukey *t*-test results and one-way ANOVA significance values.
- **Figure S2.** One-way ANOVA and Tukey *t*-test results from SuSy gene family expression comparisons.
- **Figure S3.** One-way ANOVA and Tukey *t*-test results from SPS gene family expression comparisons.
- **Figure S4.** One-way ANOVA and Tukey *t*-test results from SPP gene family expression comparisons.
- **Figure S5.** One-way ANOVA and Tukey *t*-test results from CSLA gene family expression comparisons.
- **Figure S6.** One-way ANOVA and Tukey *t*-test results from CesA gene family expression comparisons.
- **Figure S7.** One-way ANOVA and Tukey *t*-test results from CSLC gene family expression comparisons.
- **Figure S8.** One-way ANOVA and Tukey *t*-test results from CSLD gene family expression comparisons.
- **Figure S9.** One-way ANOVA and Tukey *t*-test results from CSLE gene family expression comparisons.
- **Figure S10.** One-way ANOVA and Tukey *t*-test results from CSLF gene family expression comparisons.
- **Figure S11.** One-way ANOVA and Tukey *t*-test results from CSLG gene family expression comparisons.
- **Figure S12.** One-way ANOVA and Tukey *t*-test results from CSLH gene family expression comparisons.
- **Figure S13.** One-way ANOVA and Tukey *t*-test results from UGD gene family expression comparisons.
- **Figure S14.** One-way ANOVA and Tukey *t*-test results from UGE gene family expression comparisons.
- **Figure S15.** One-way ANOVA and Tukey *t*-test results from UXE gene family expression comparisons.
- **Figure S16.** One-way ANOVA and Tukey *t*-test results from RHM gene family expression comparisons.
- **Figure S17.** One-way ANOVA and Tukey *t*-test results from GALE gene family expression comparisons.
- **Figure S18.** One-way ANOVA and Tukey *t*-test results from UXS gene family expression comparisons.
- **Figure S19.** One-way ANOVA and Tukey *t*-test results from UAXS gene family expression comparisons.
- **Figure S20.** One-way ANOVA and Tukey *t*-test results from MIPS gene family expression comparisons.
- **Figure S21.** One-way ANOVA and Tukey *t*-test results from GluK gene family expression comparisons.
- **Figure S22.** One-way ANOVA and Tukey *t*-test results from MIP gene family expression comparisons.
- **Figure S23.** One-way ANOVA and Tukey *t*-test results from MIOX gene family expression comparisons.
- **Figure S24.** One-way ANOVA and Tukey *t*-test results from Invertase gene family ANINV.
- **Figure S25.** One-way ANOVA and Tukey *t*-test results from Invertase gene family CINV.
- **Figure S26.** One-way ANOVA and Tukey *t*-test results from Invertase gene family CWI.
- **Figure S27.** One-way ANOVA and Tukey *t*-test results from Invertase gene family VINV.
- **Figure S28.** One-way ANOVA and Tukey *t*-test results from UGPase gene family expression comparisons.
- **Figure S29.** One-way ANOVA and Tukey *t*-test results from AGP gene family expression comparisons.
- **Figure S30.** One-way ANOVA and Tukey *t*-test results from PGM gene family expression comparisons.
- **Figure S31.** One-way ANOVA and Tukey *t*-test results from G6PI gene family expression comparisons.
- **Figure S32.** One-way ANOVA and Tukey *t*-test results from HXK gene family expression comparisons.
- **Figure S33.** One-way ANOVA and Tukey *t*-test results from FK gene family expression comparisons.

**Table S1.** Variety factsheet for Q208 and KQ228 from QCANESelect™ variety selection webpage.

| **VARIETY** | **Q208** | **KQ228** |
| --- | --- | --- |
| **PARENTAGE** | Q135 x QN61-1232 | QN80-3425 x CP74-2005 |
| **SEEDLING CODE** | QA87-1413 | KQ98-673 |
| **SMUT INFORMATION** | Intermediate-Resistant | Resistant |
| **APPEARANCE** | Green, average to thin stalks, yellow when highly exposed, Light wax covering. Growth cracks present. Open stool. Dark green erect canopy. Hairless to some hairs present | Yellow-green stalk with noticeable white wax. Leaves blue-green with sharp edges |
| **FEATURES/COMMENTS** | Information presented based on Burdekin and Central QLD variety guides. Results indicate above average yields and average sugar content. Free trashing with average amounts of trash | Good early sugar. Fast Reliable germination. Heavy suckering late in season |
| **HARVESTING TIME** | Early, Mid, Late | Early, Mid |
| **SUITED SOILS** | High Quality Soils, Medium Quality, Poor Soils | Cracking Clay, Non-Sodic Duplex, Silty Loam, Sodic Duplex, Sodic/ Sand/Saline |
| **SEASONAL SUGAR:** |  | |
| **EARLY** | Average | Good |
| **MID** | Good | Average |
| **LATE** | Good | Poor |
| **PRODUCTIVITY:** |  | |
| **YIELD** | High | High |
| **CCS** | Moderate - High | Moderate |
| **FAST AND RELIABLE GERMINATION:** | Average | Good |
| **AFTER EARLY HARVEST** | Good | N/A |
| **UNDER WET CONDITIONS** | Good | N/A |
| **LODGING TOLERANCE** | Average | Average |
| **CROP AGE** | 1 or 2 years | N/A |
| **FLOWERING** | Sparse | N/A |
| **TRASH YIELD** | N/A | N/A |
| **REACTION TO STRESS:** |  | |
| **WATERLOGGING** | Good | Average |
| **DROUGHT** | Good | N/A |
| **FROST** | Average | Average |
| **FLOOD** | Average | N/A |
| **DISEASE REACTION:** |  | |
| **RESISTANT** | Brown Rust, Chlorotic Streak, Leaf Scald, Orange Rust, Red Rot, RSD | Brown Rust, Leaf Scald, Red Rot, Smut |
| **INTERMEDIATE-RESISTANT** | Smut | N/A |
| **INTERMEDIATE** | Pachymetra | N/A |
| **INTERMEDIATE-SUSCEPTABLE** | Fiji Leaf Gall | N/A |
| **SUSCEPTABLE** | Lesion Nematode, Root Knot Nematode | Lesion Nematode, Root Knot Nematode |
| **CANE GRUB TOLERANCE** | Unknown | Average |
| **HERBICIDE REACTION** | Shows tolerance to herbicides | Growth severely affected with ametryn applications. Use directed sprays to minimize contact. |
| **CROP MANAGEMENT PRACTICES** | N/A | Very high early sugar-harvest when possible |

**Figure S1.** Schematic of sugarcane transcript gene family homologue assignment and quantification.

**Table S2.** Numerical Identifier for Tukey *t*-test results and one-way ANOVA significance values.

| **Tissue/Organ Type** | **Numerical Identifier** |
| --- | --- |
| Bottom Internode | 1 |
| Middle Internode | 2 |
| Top Internode | 3 |
| Root | 4 |
| 1st Visible Dewlap Leaf | 5 |
| 5th Visible Dewlap Leaf | 6 |

**
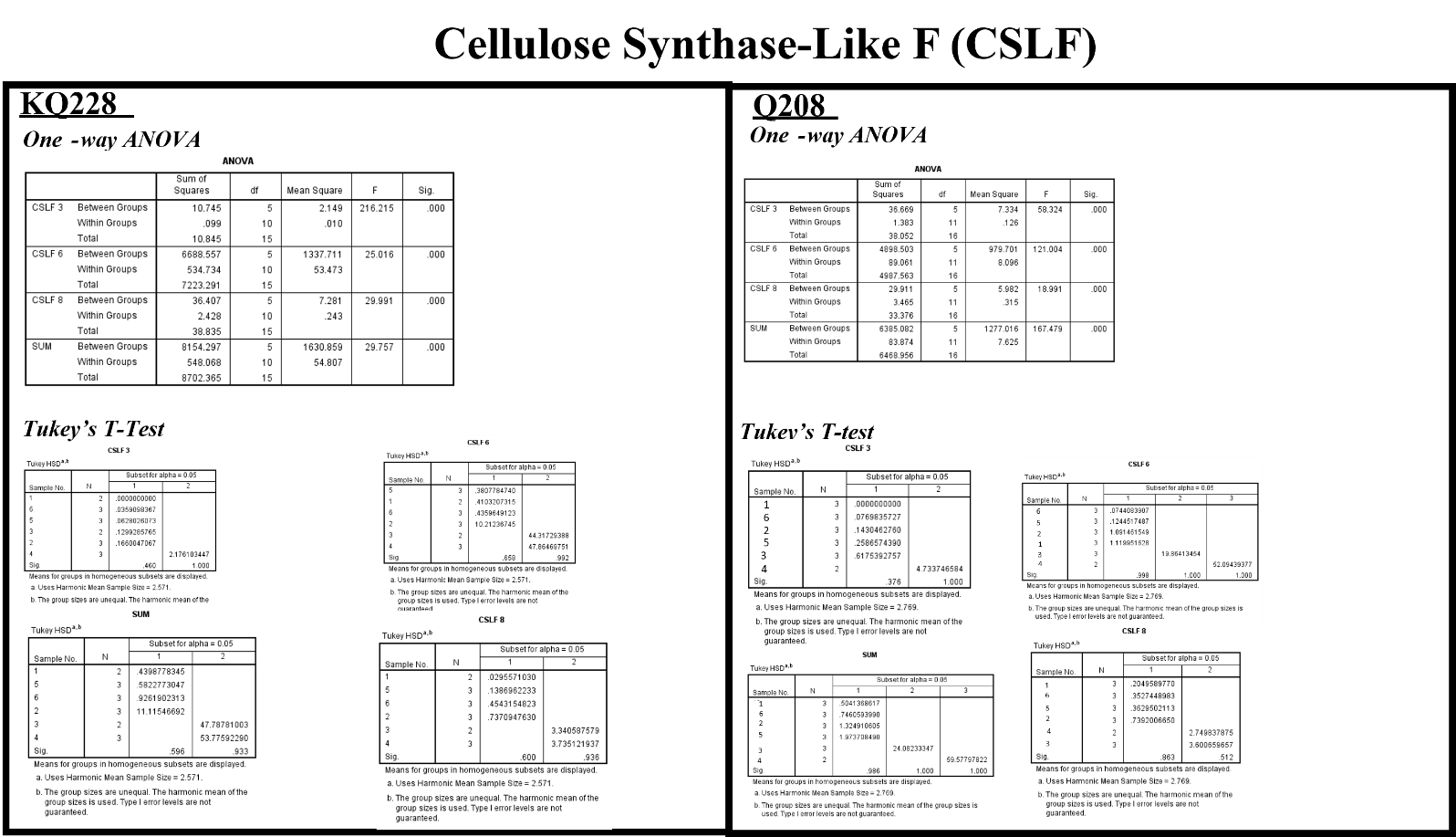

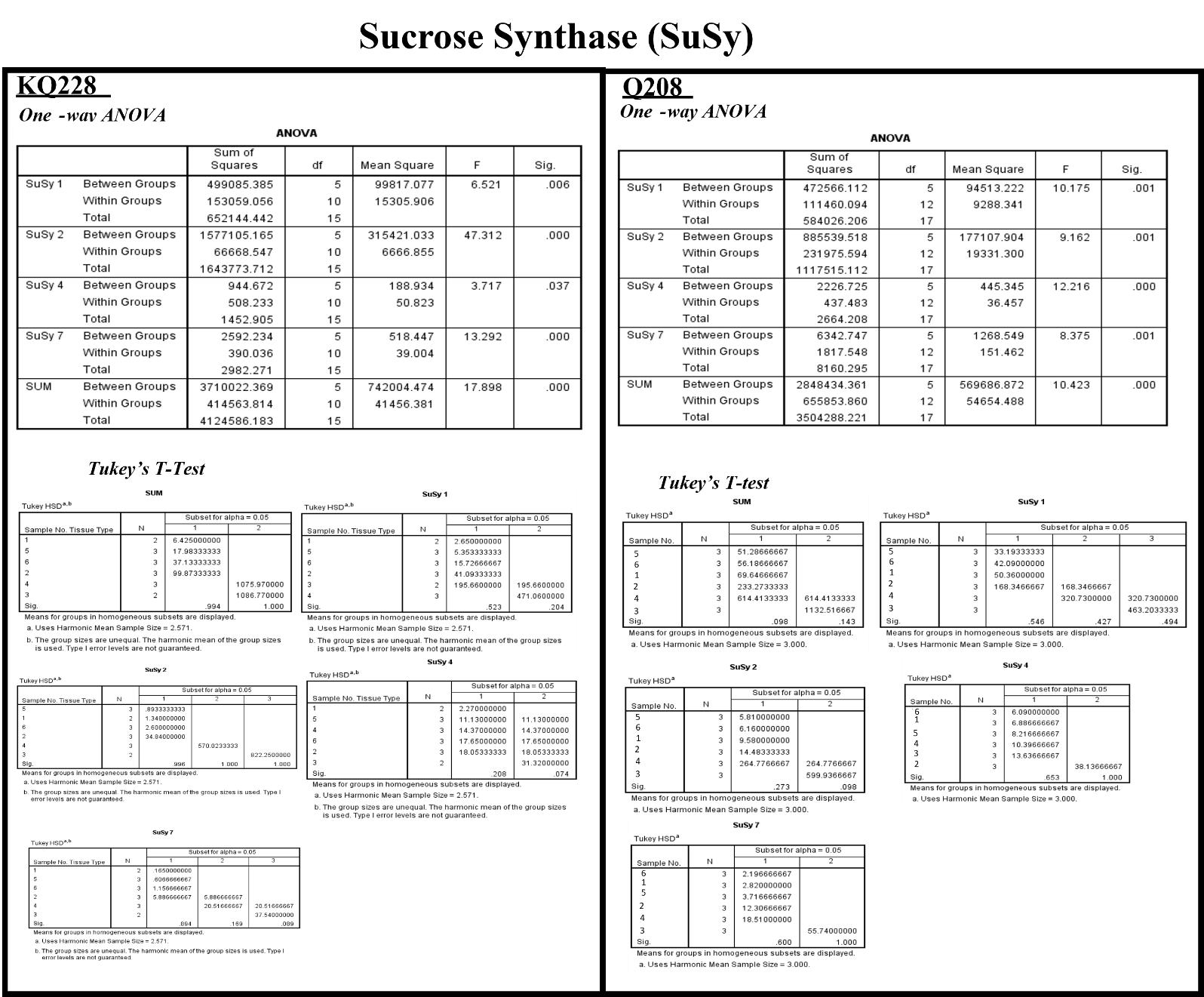
**

**Figure S2.** One-way ANOVA and Tukey *t*-test results from SuSy gene family expression comparisons.


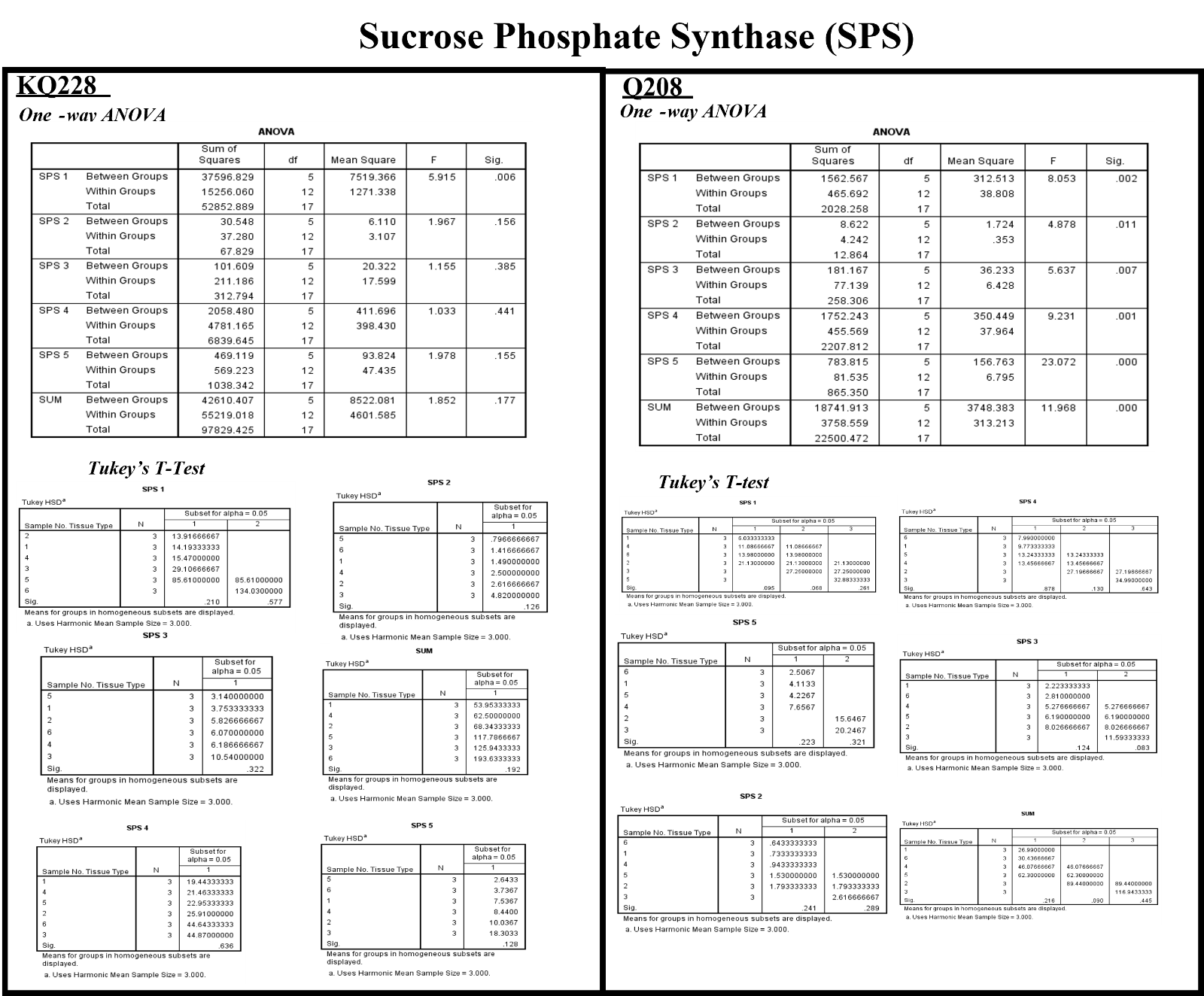


**Figure S3.** One-way ANOVA and Tukey *t*-test results from SPS gene family expression comparisons.


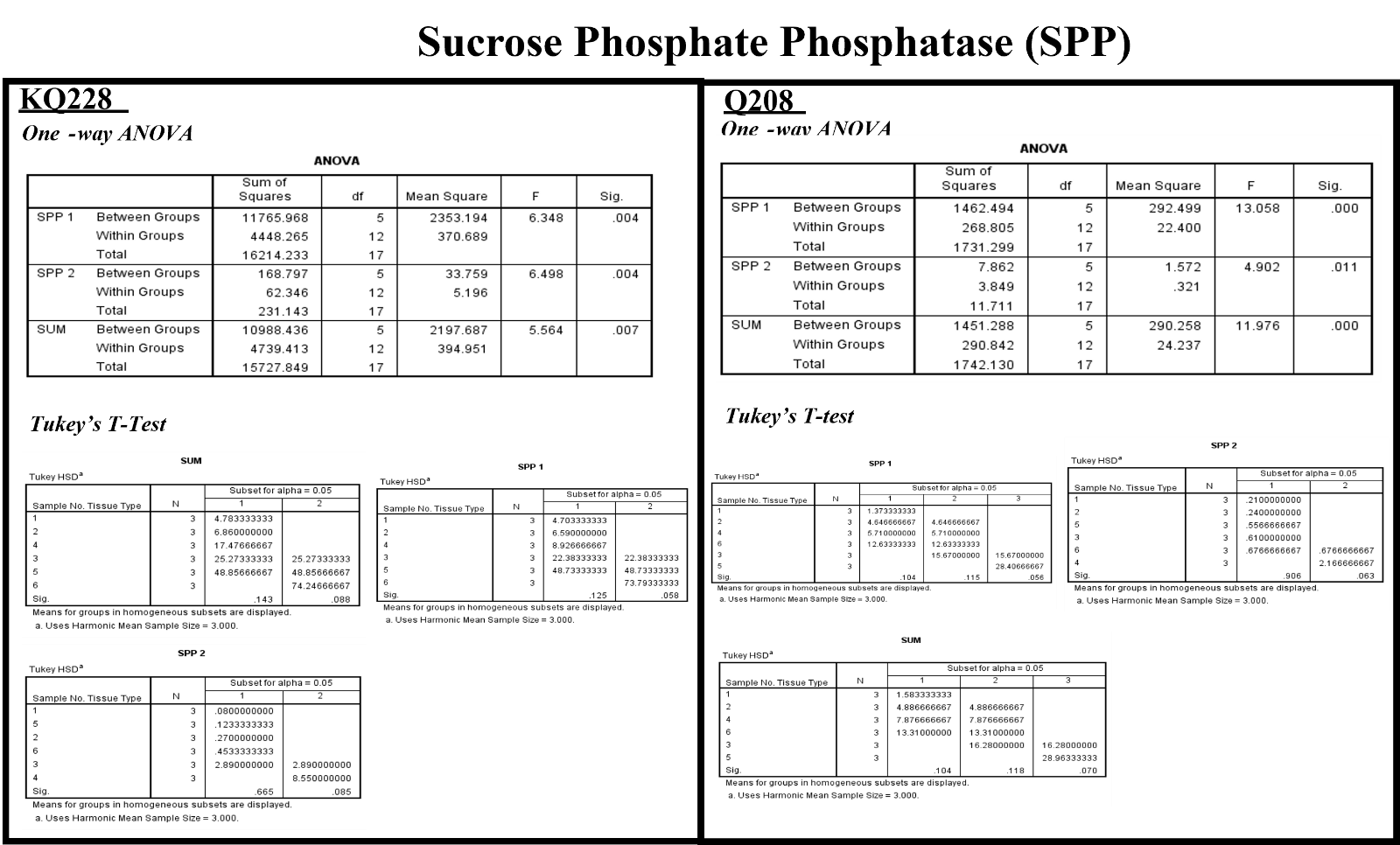


**Figure S4.** One-way ANOVA and Tukey *t*-test results from SPP gene family expression comparisons.


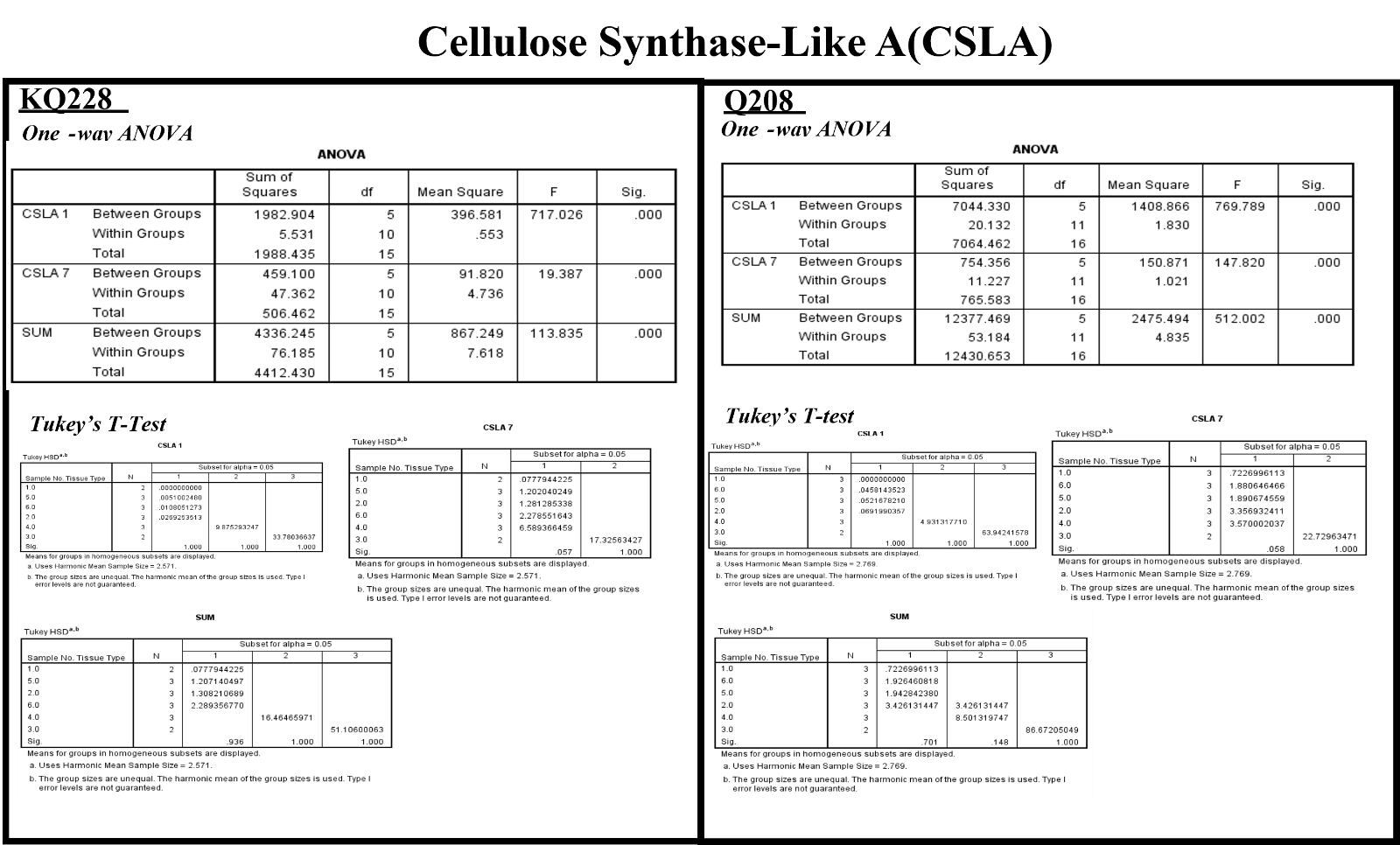


**Figure S5.** One-way ANOVA and Tukey *t*-test results from CSLA gene family expression comparisons.


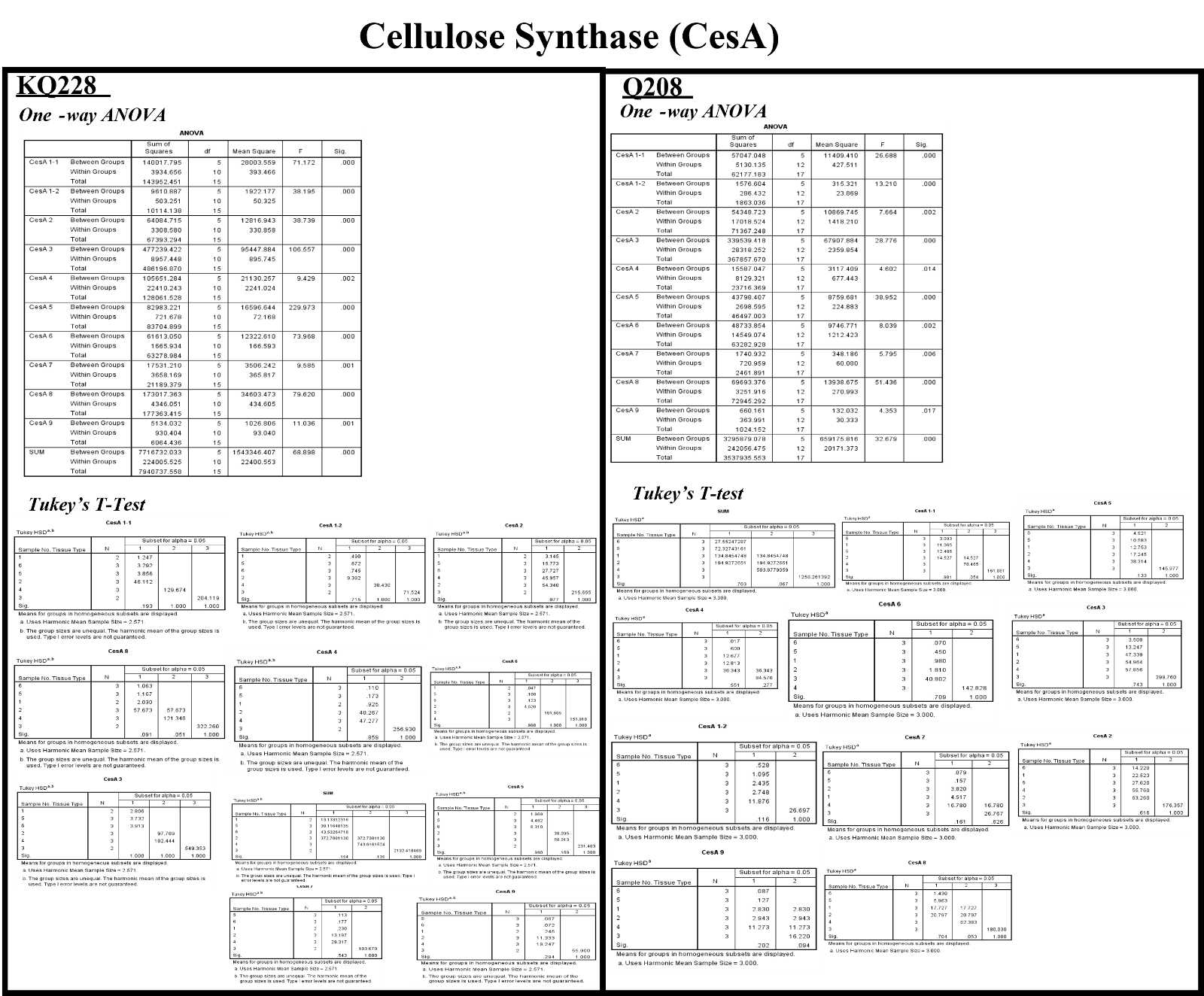


**Figure S6.** One-way ANOVA and Tukey *t*-test results from CesA gene family expression comparisons.


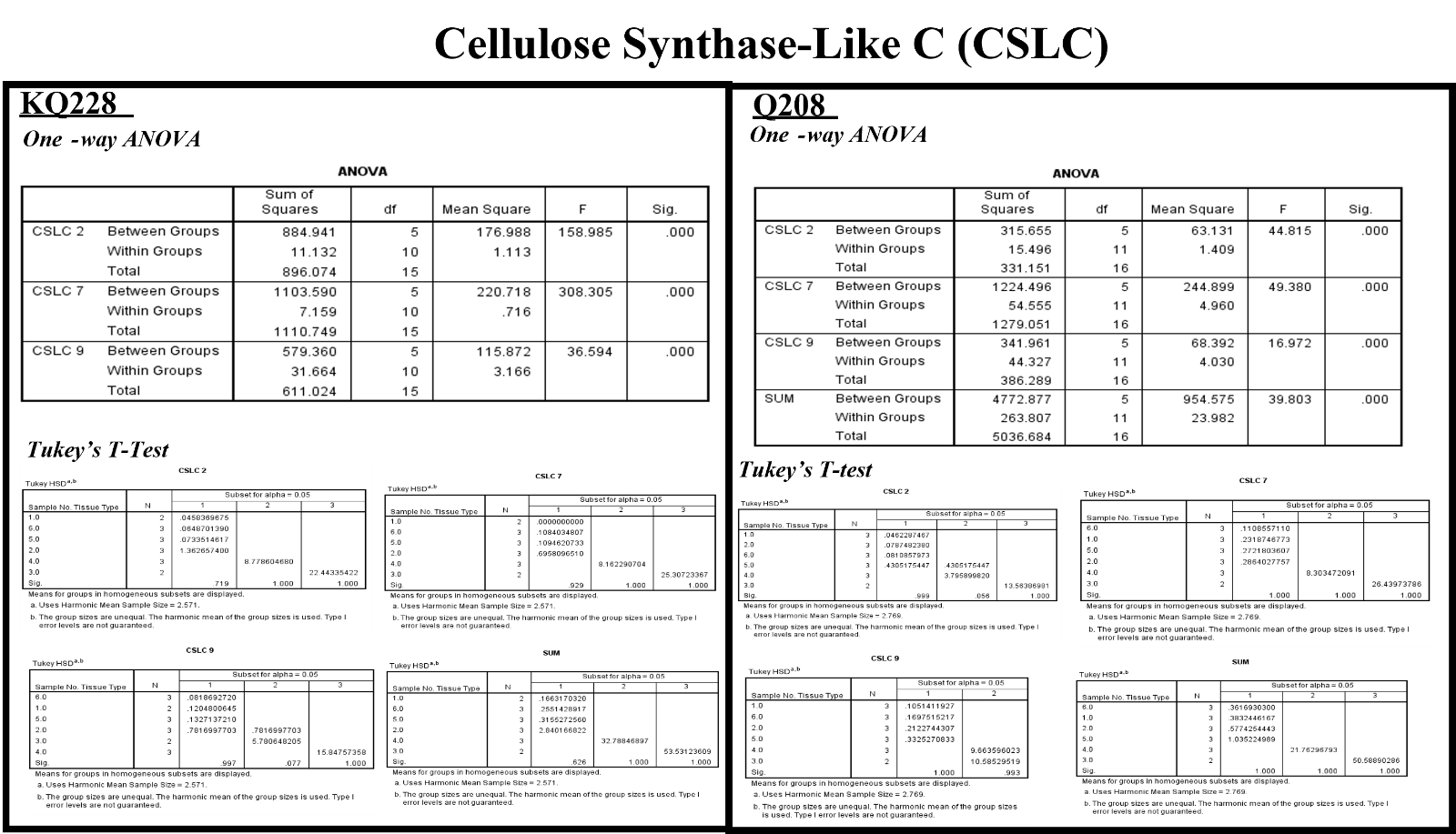


**Figure S7.** One-way ANOVA and Tukey *t*-test results from CSLC gene family expression comparisons.


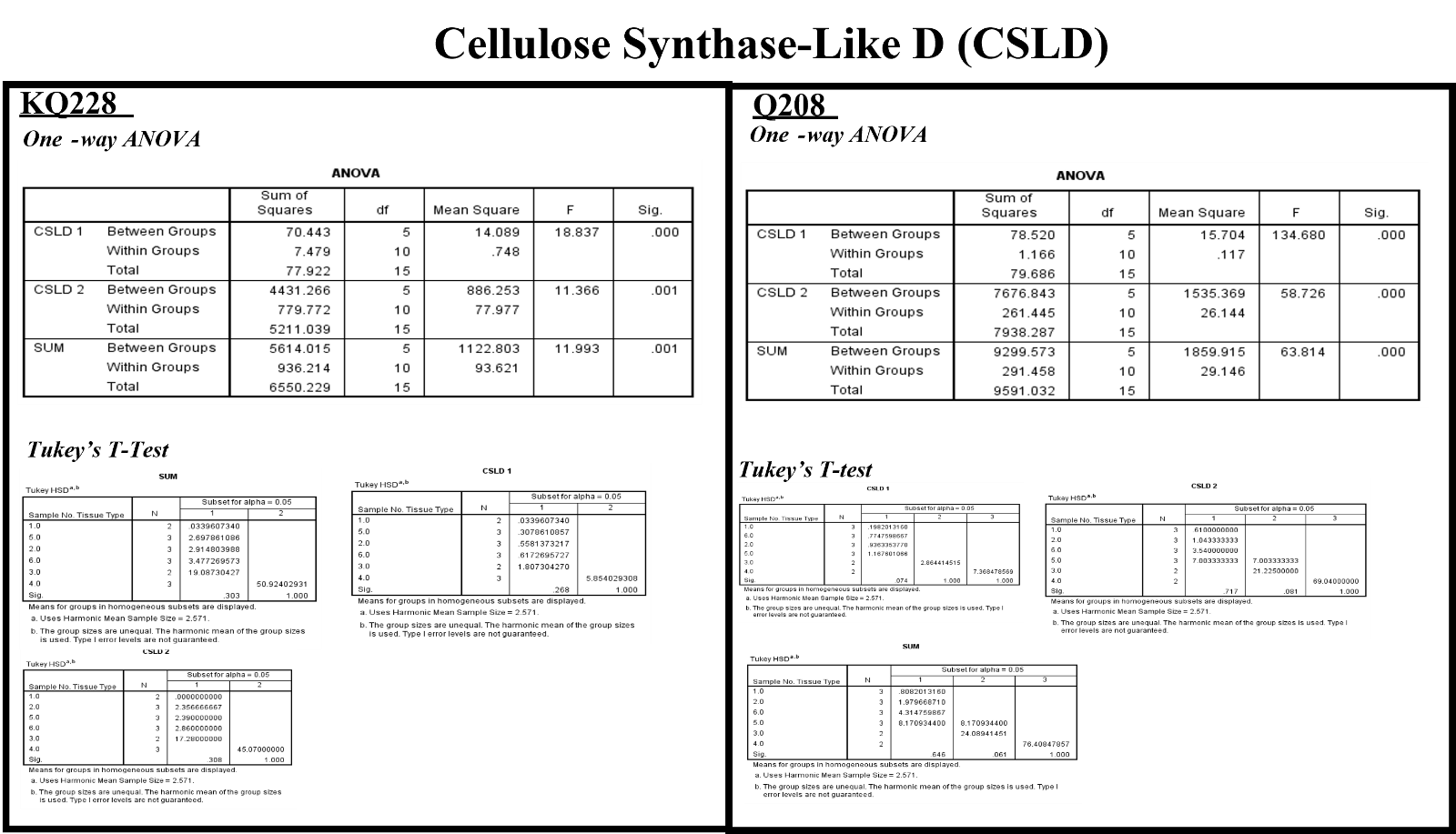


**Figure S8.** One-way ANOVA and Tukey *t*-test results from CSLD gene family expression comparisons.


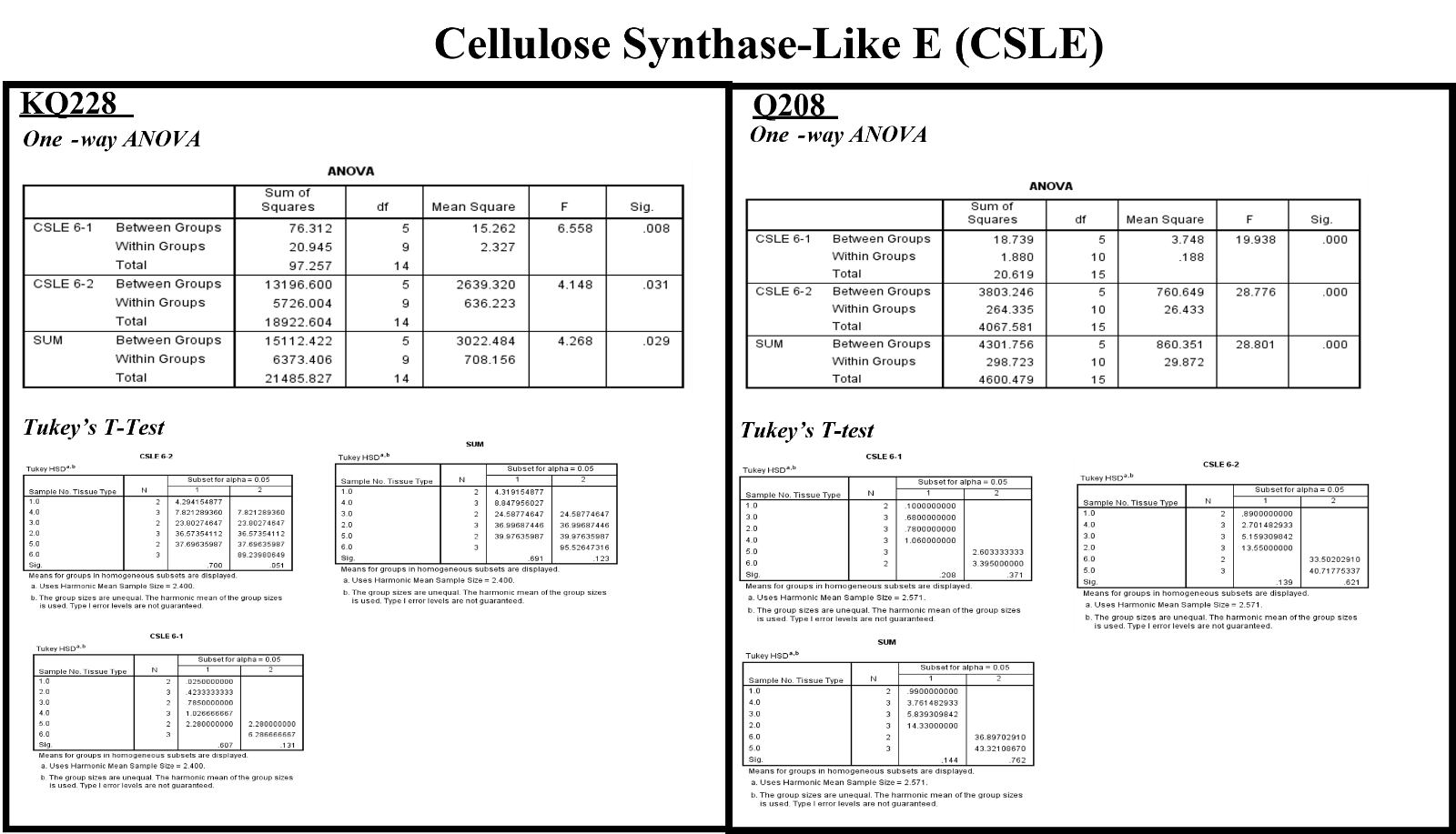


**Figure S9.** One-way ANOVA and Tukey *t*-test results from CSLE gene family expression comparisons.


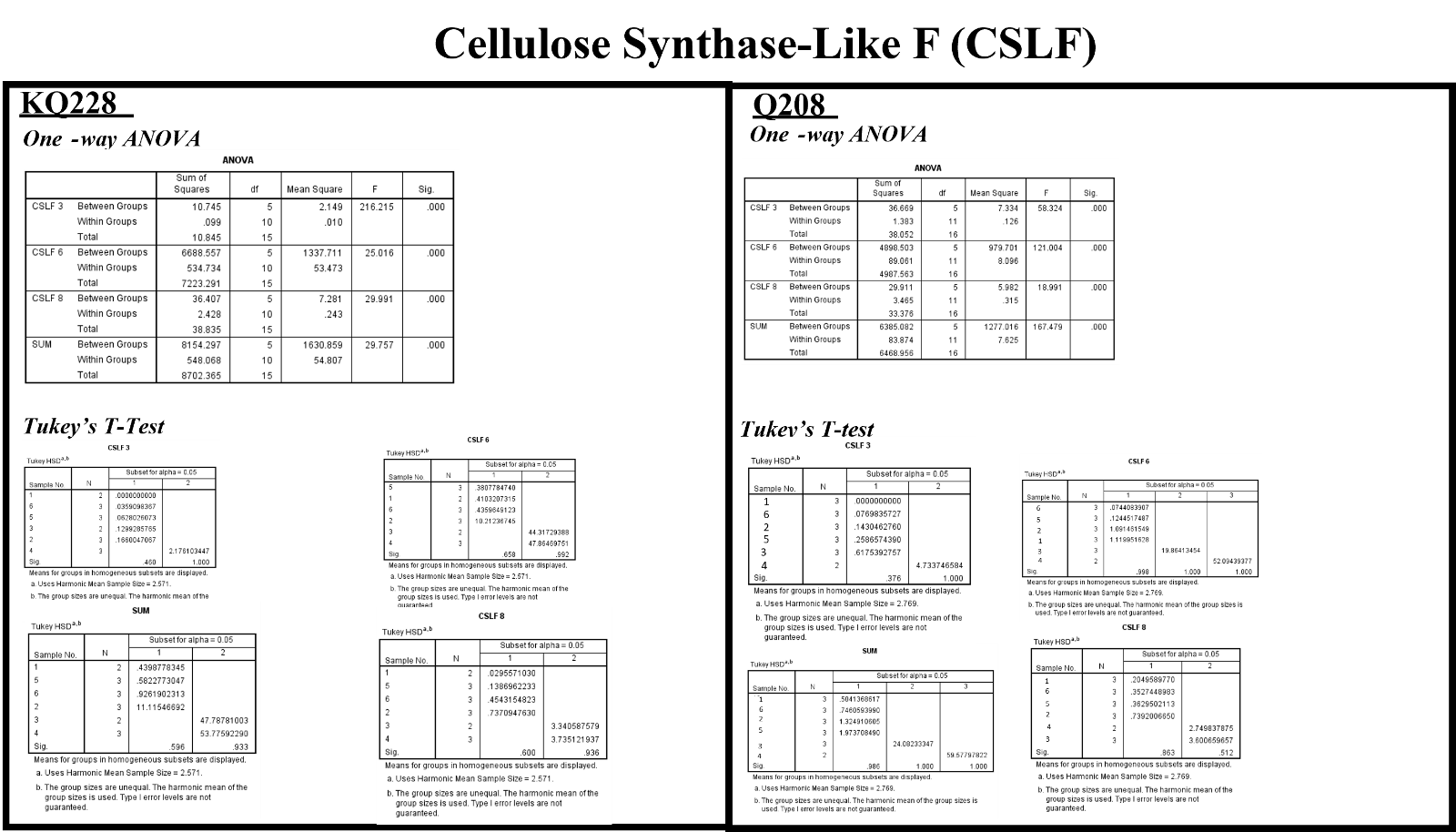


**Figure S10.** One-way ANOVA and Tukey *t*-test results from CSLF gene family expression comparisons.


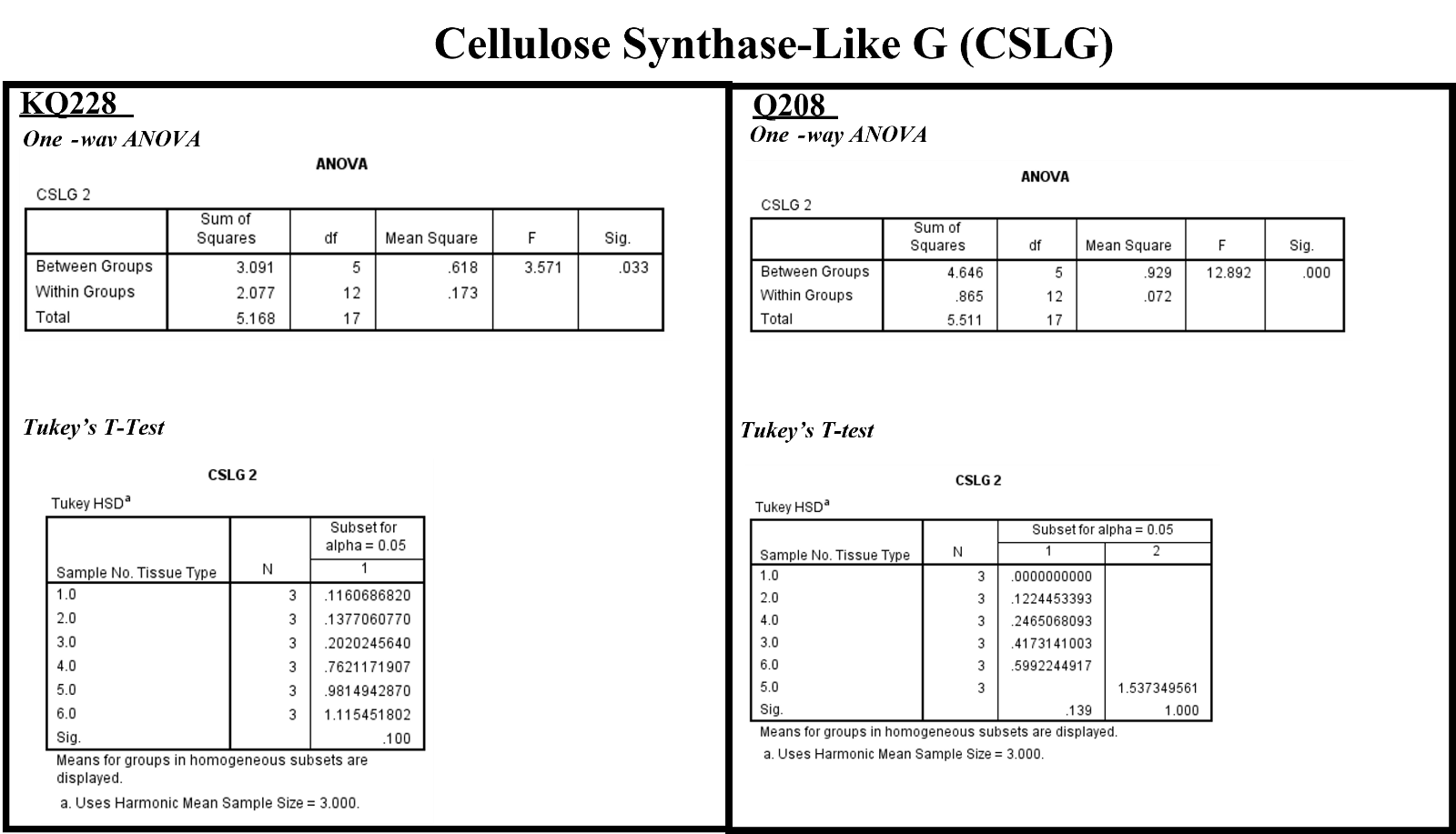


**Figure S11.** One-way ANOVA and Tukey *t*-test results from CSLG gene family expression comparisons.


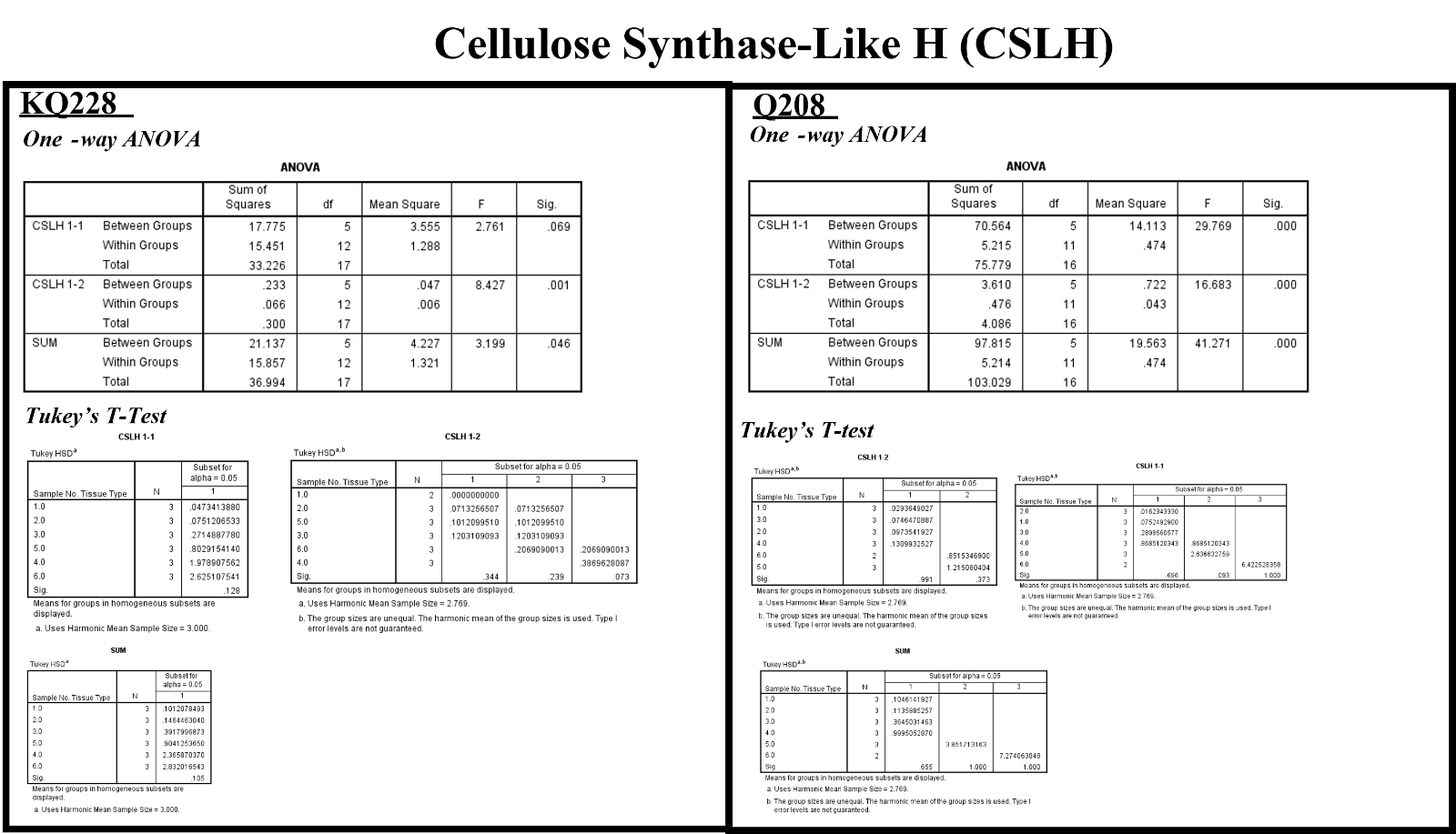


**Figure S12.** One-way ANOVA and Tukey *t*-test results from CSLH gene family expression comparisons.


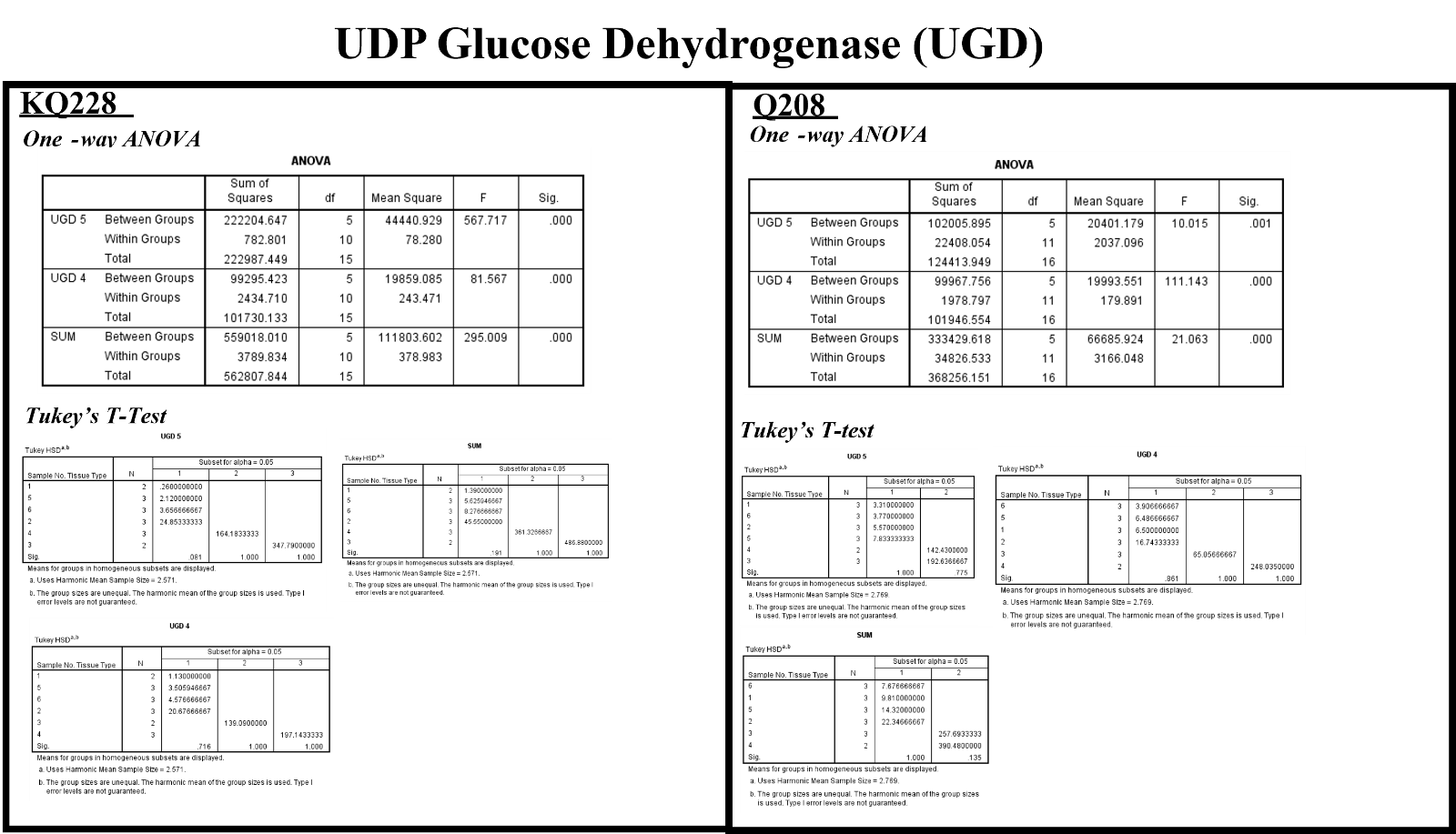


**Figure S13.** One-way ANOVA and Tukey *t*-test results from UGD gene family expression comparisons.


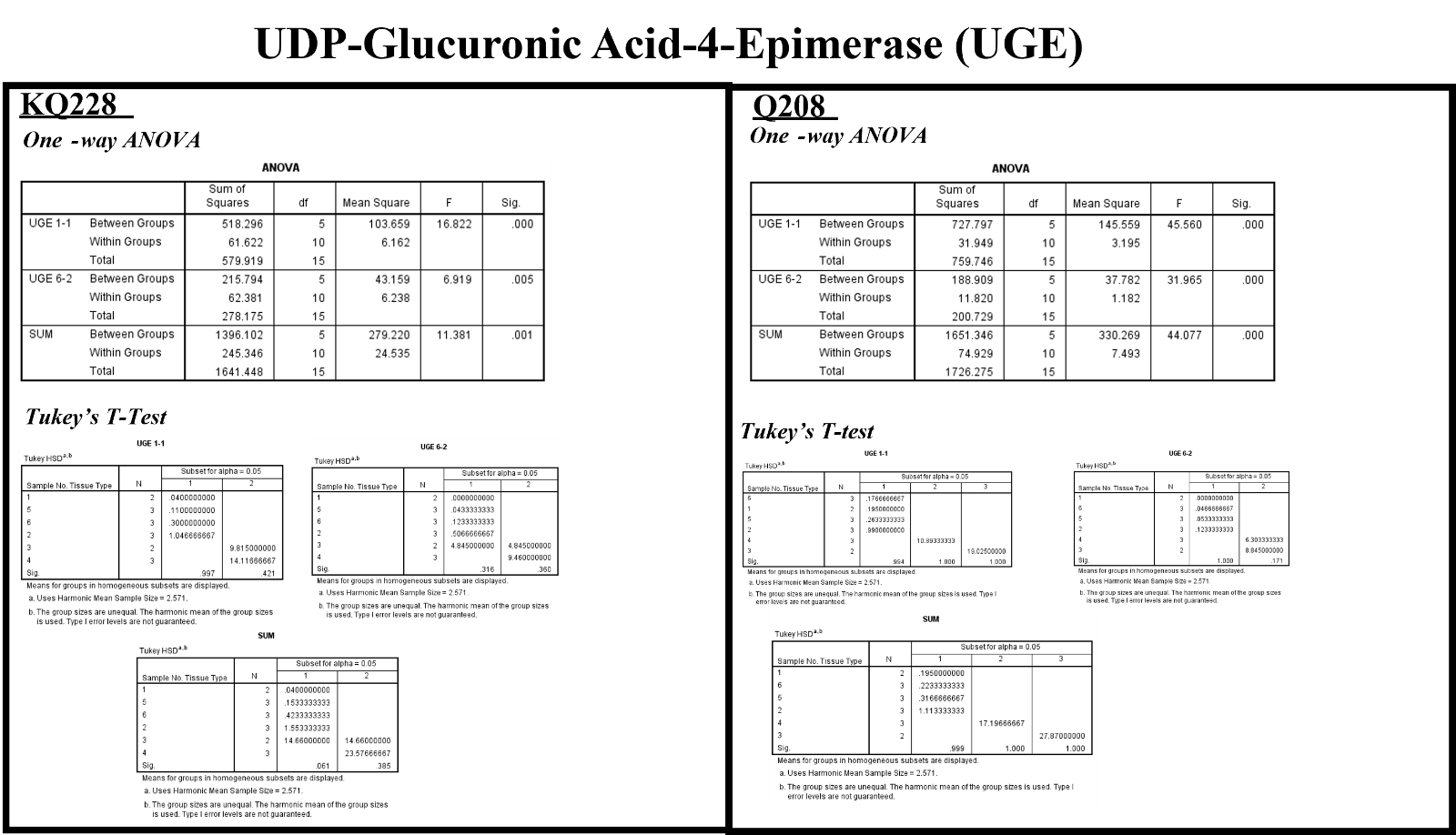


**Figure S14.** One-way ANOVA and Tukey *t*-test results from UGE gene family expression comparisons.


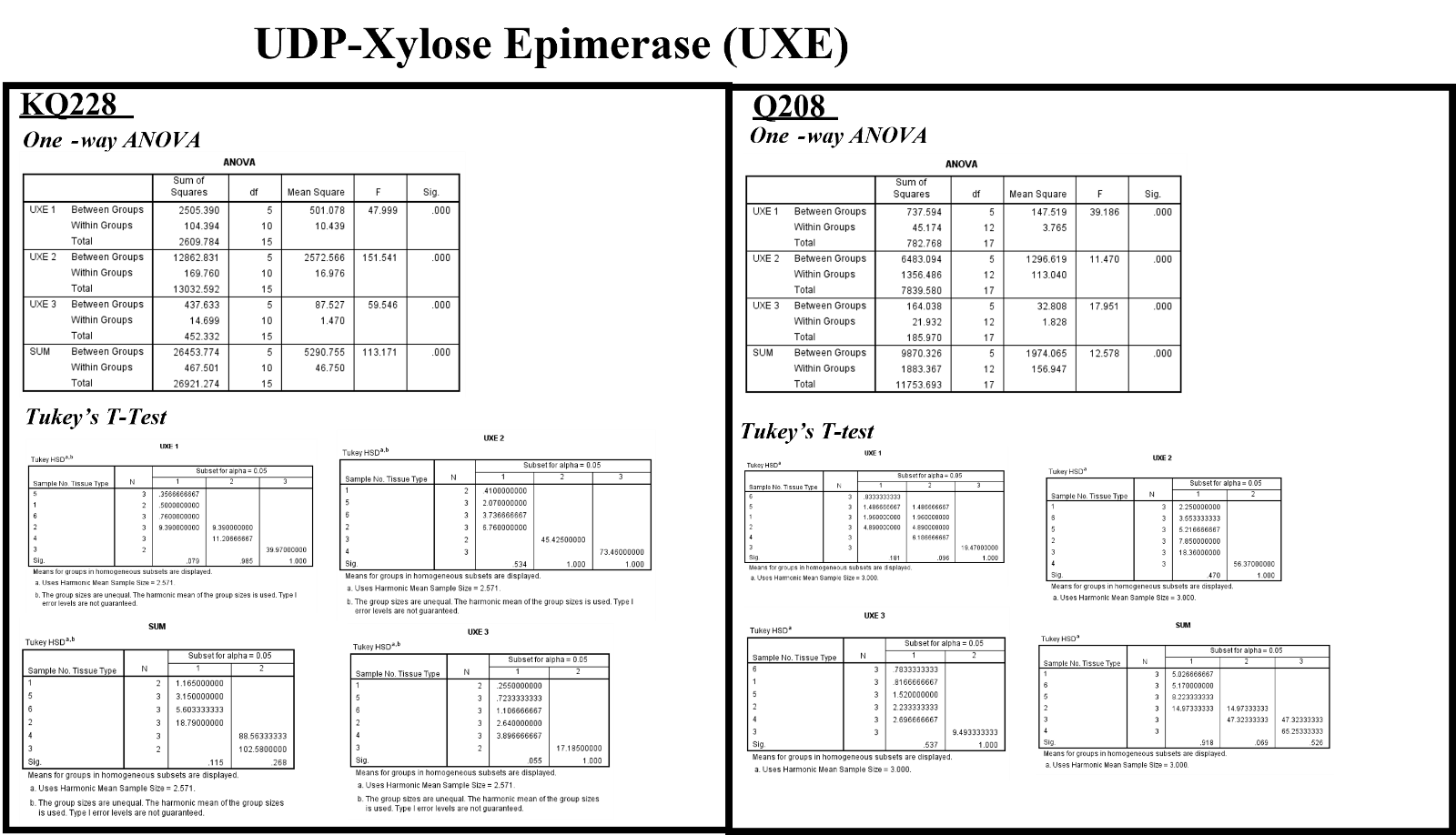


**Figure S15.** One-way ANOVA and Tukey *t*-test results from UXE gene family expression comparisons.


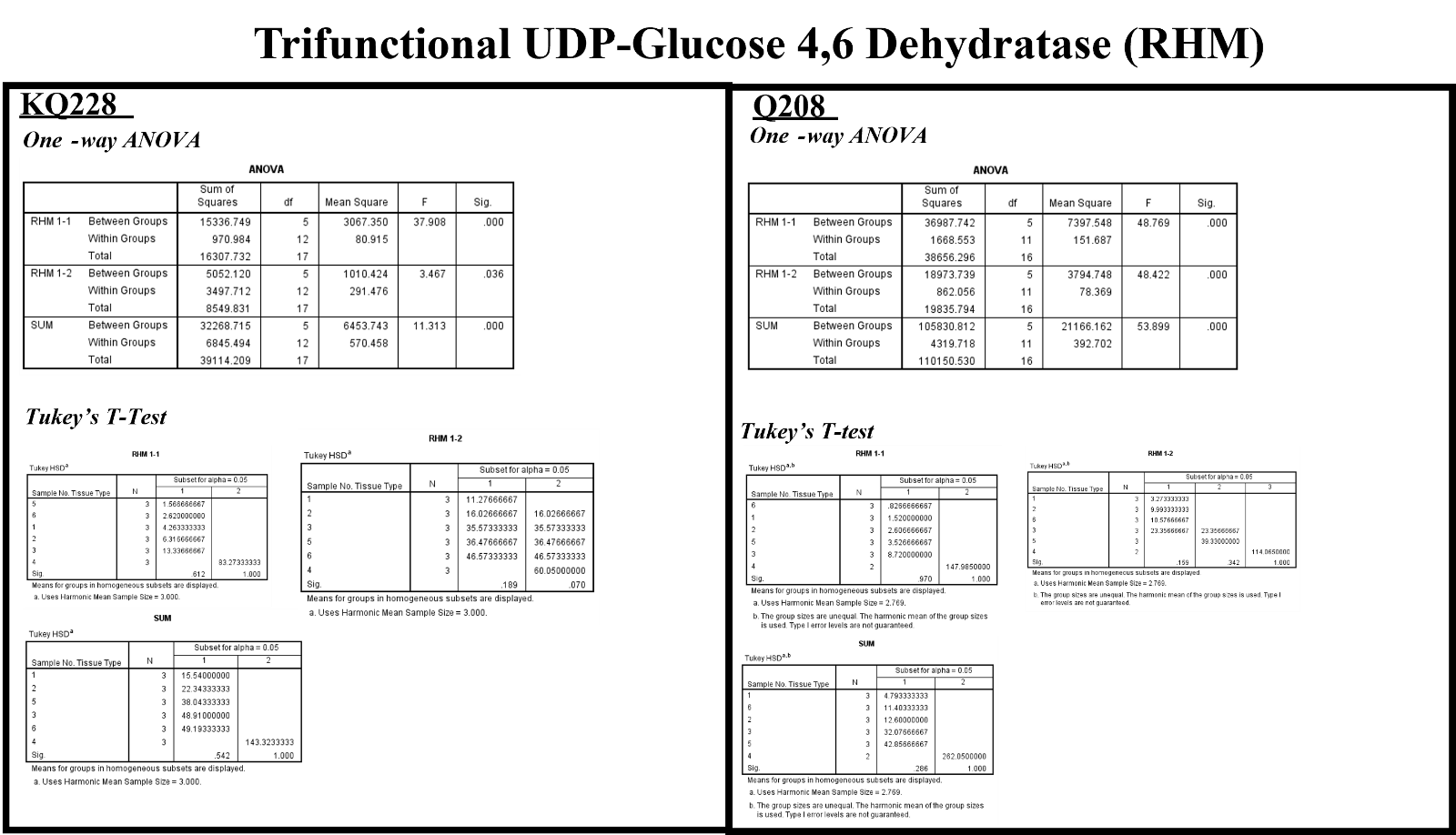


**Figure S16.** One-way ANOVA and Tukey *t*-test results from RHM gene family expression comparisons.


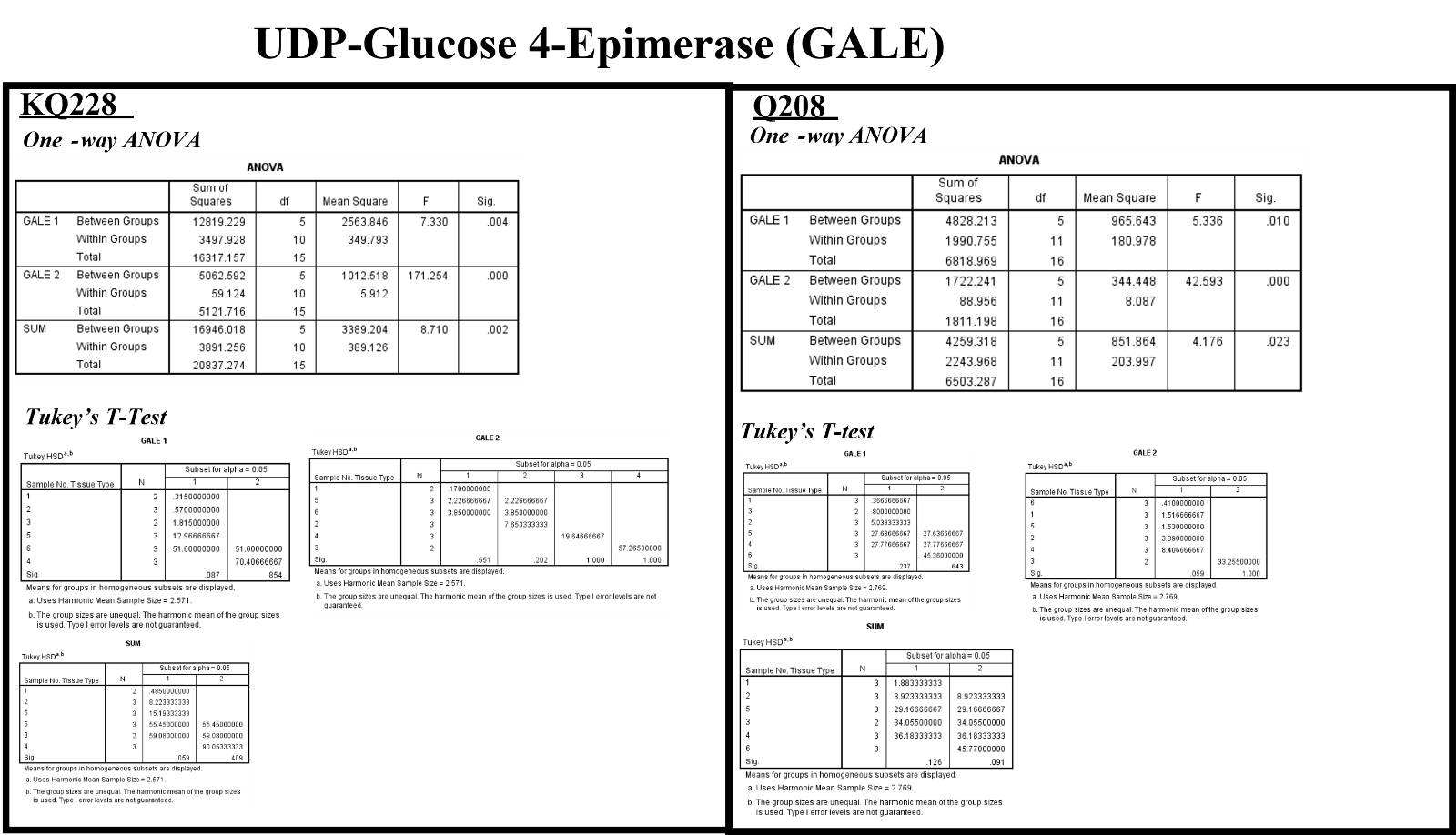


**Figure S17.** One-way ANOVA and Tukey *t*-test results from GALE gene family expression comparisons.


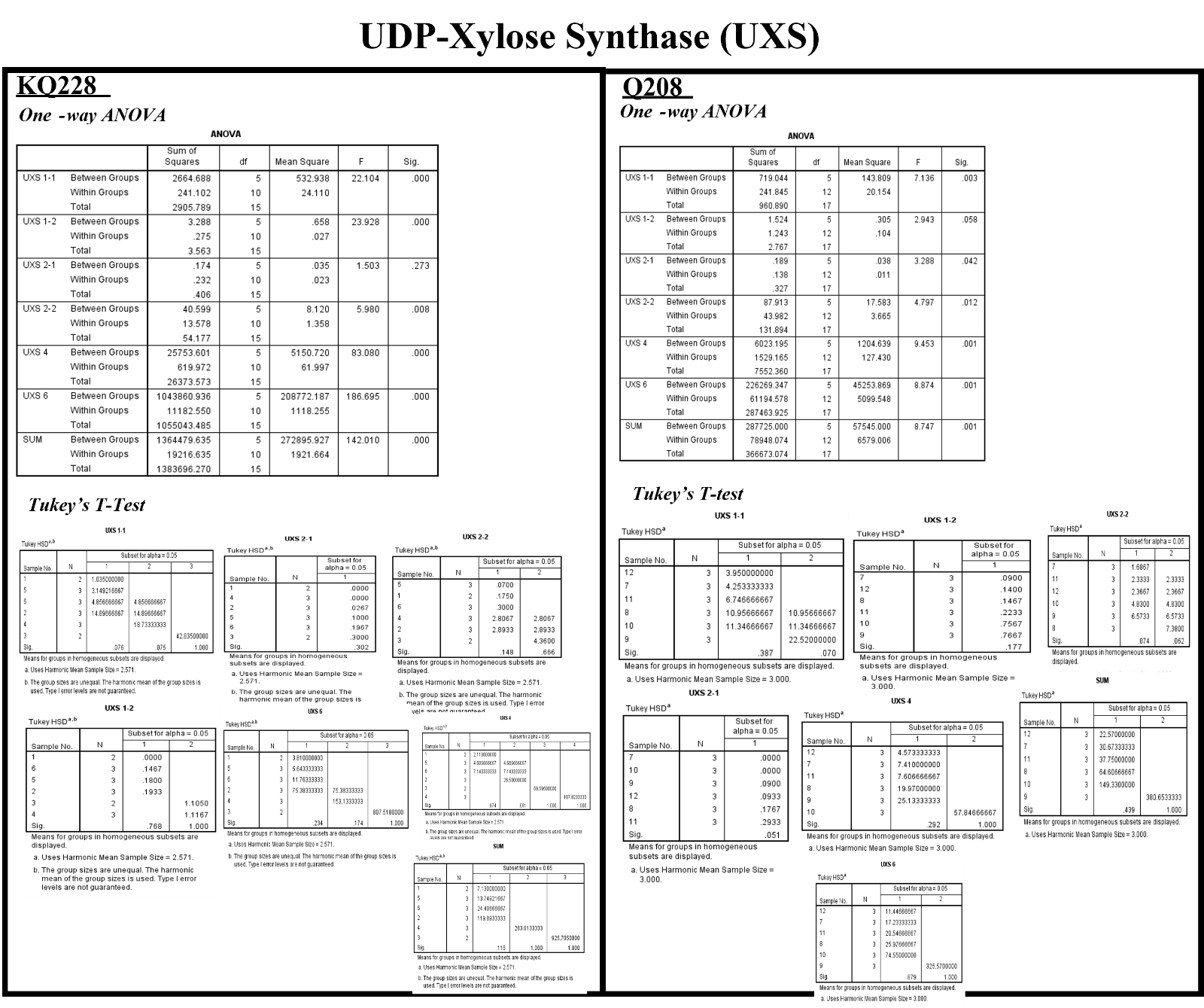


**Figure S18.** One-way ANOVA and Tukey *t*-test results from UXS gene family expression comparisons.


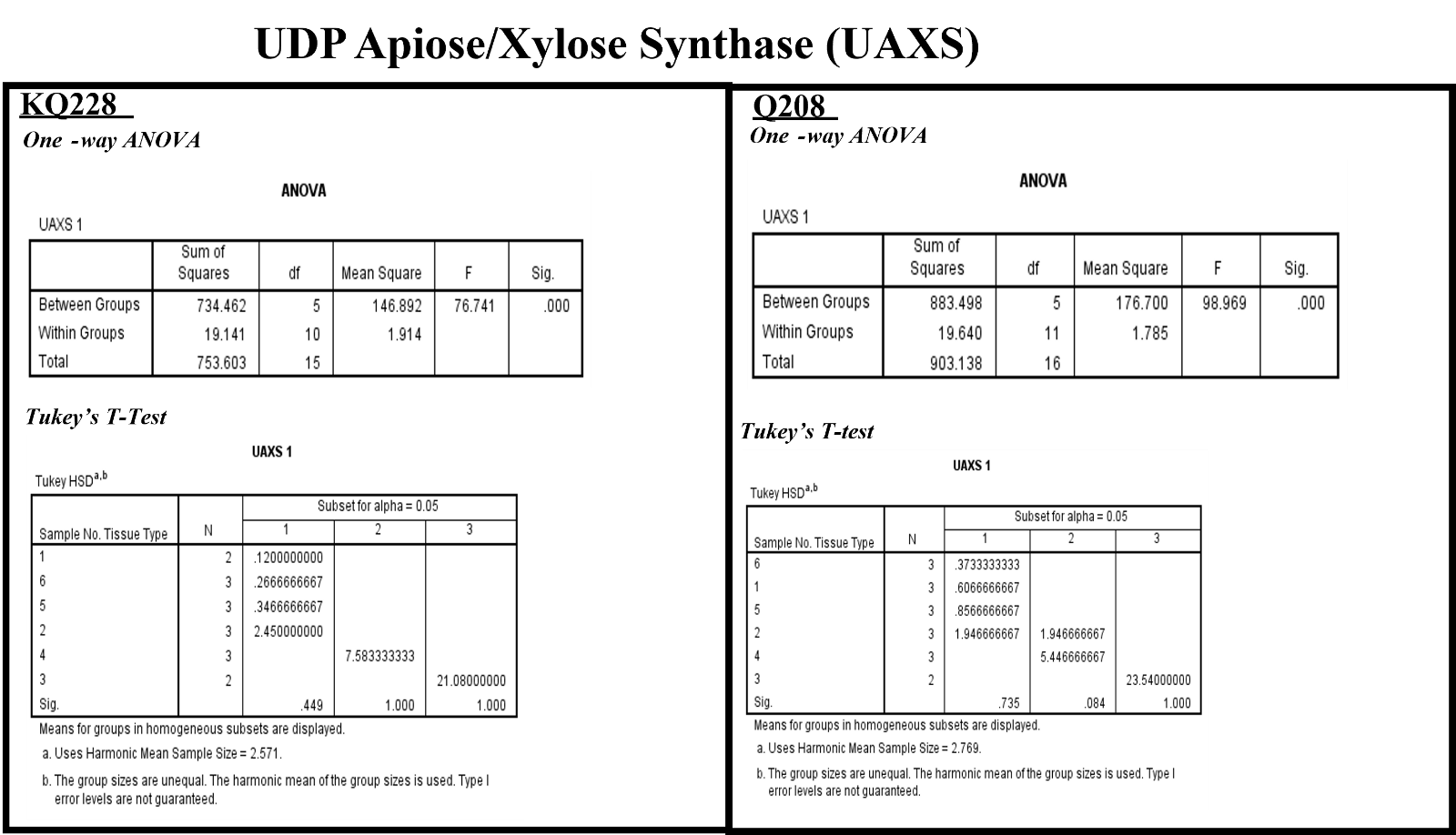


**Figure S19.** One-way ANOVA and Tukey *t*-test results from UAXS gene family expression comparisons.


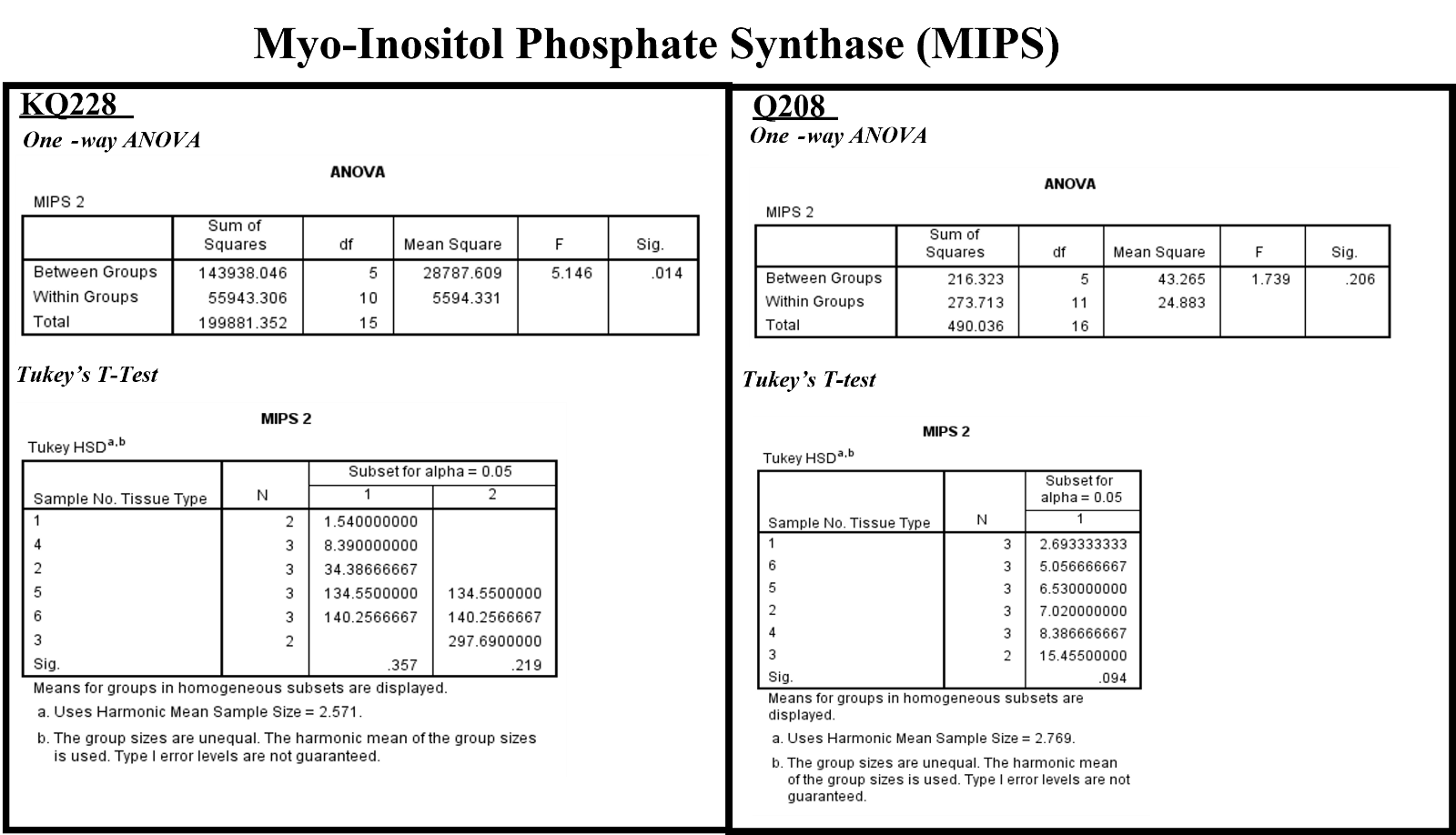


**Figure S20.** One-way ANOVA and Tukey *t*-test results from MIPS gene family expression comparisons.


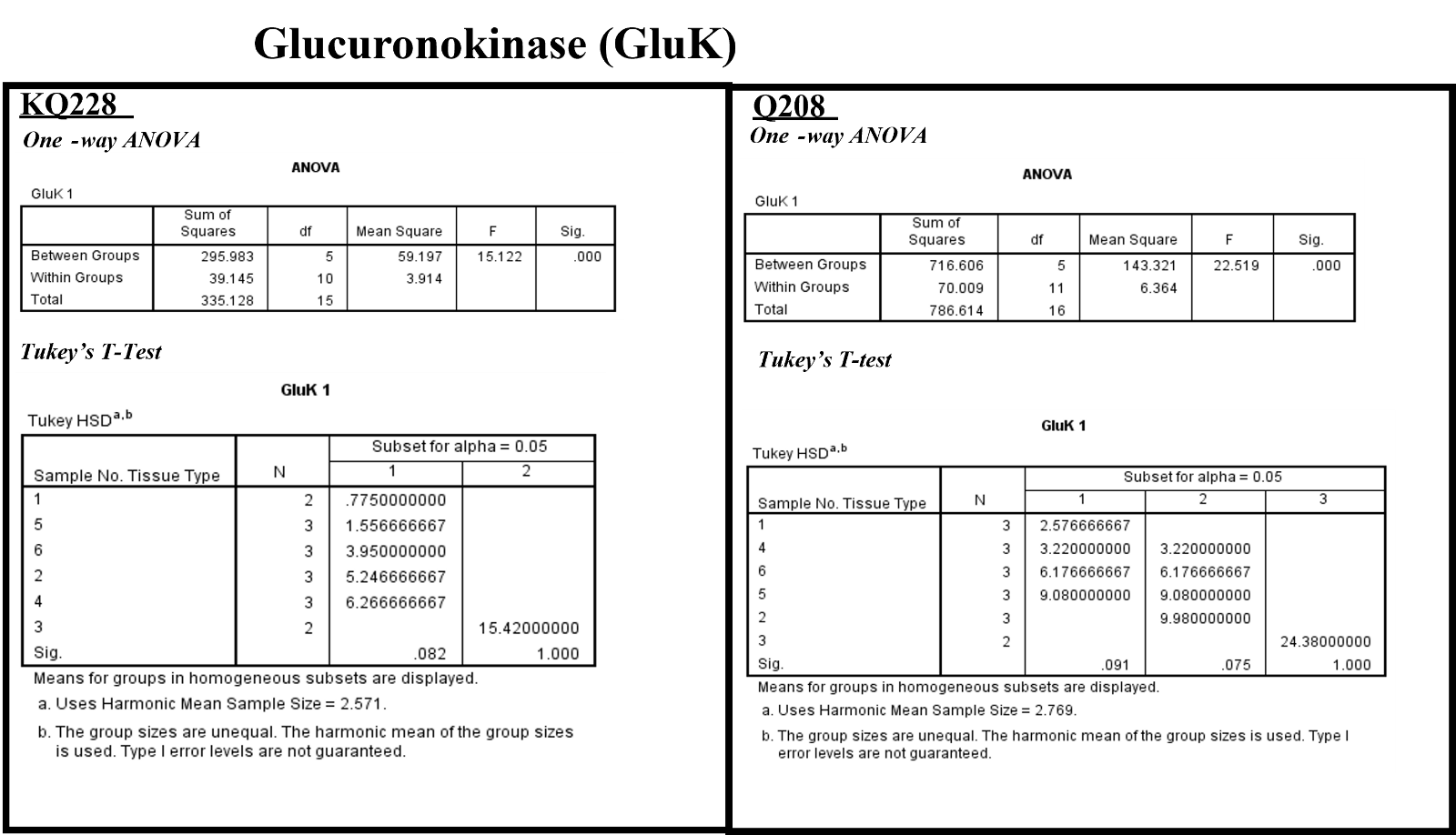


**Figure S21.** One-way ANOVA and Tukey *t*-test results from GluK gene family expression comparisons.


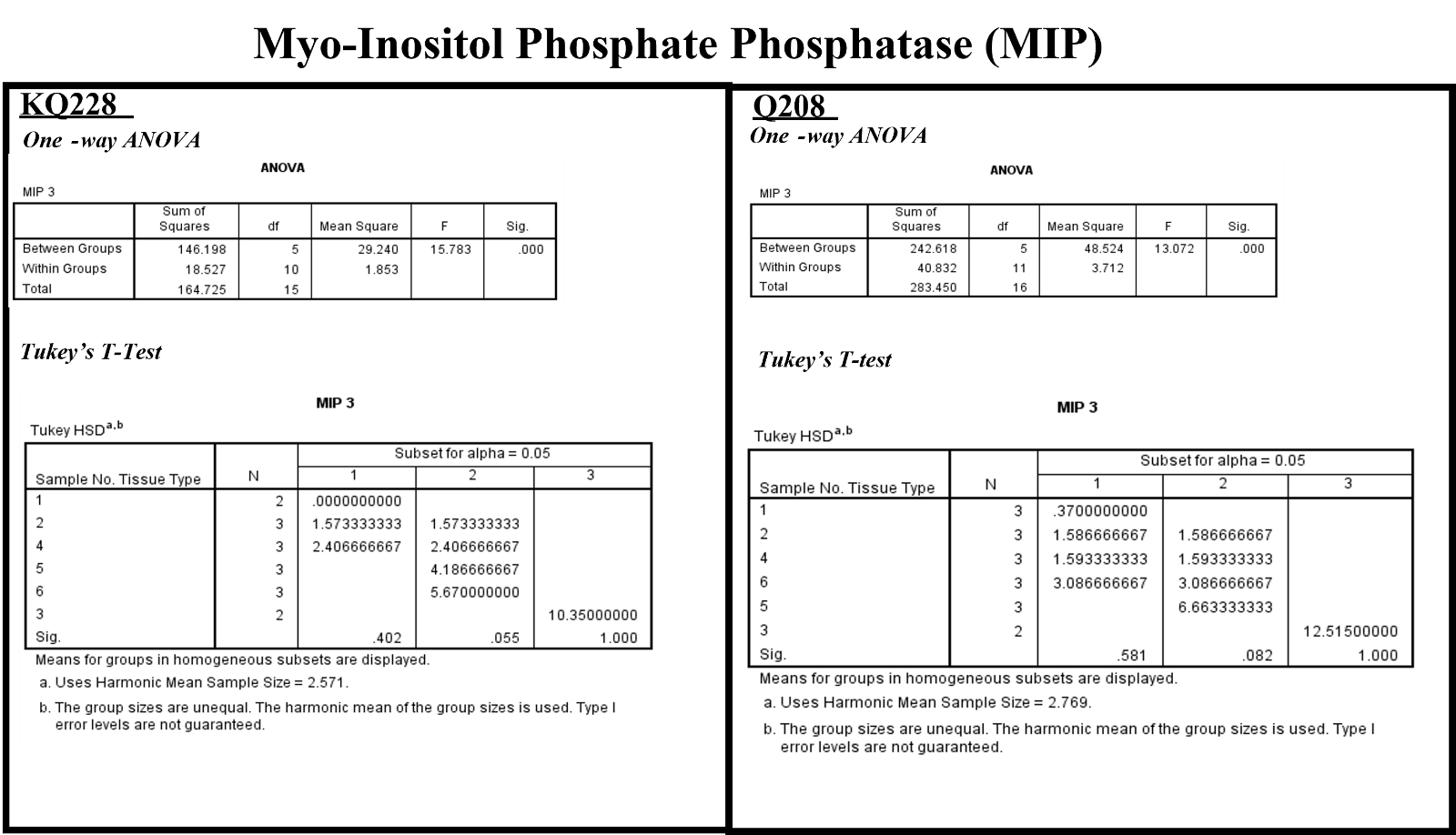


**Figure S22.** One-way ANOVA and Tukey *t*-test results from MIP gene family expression comparisons.


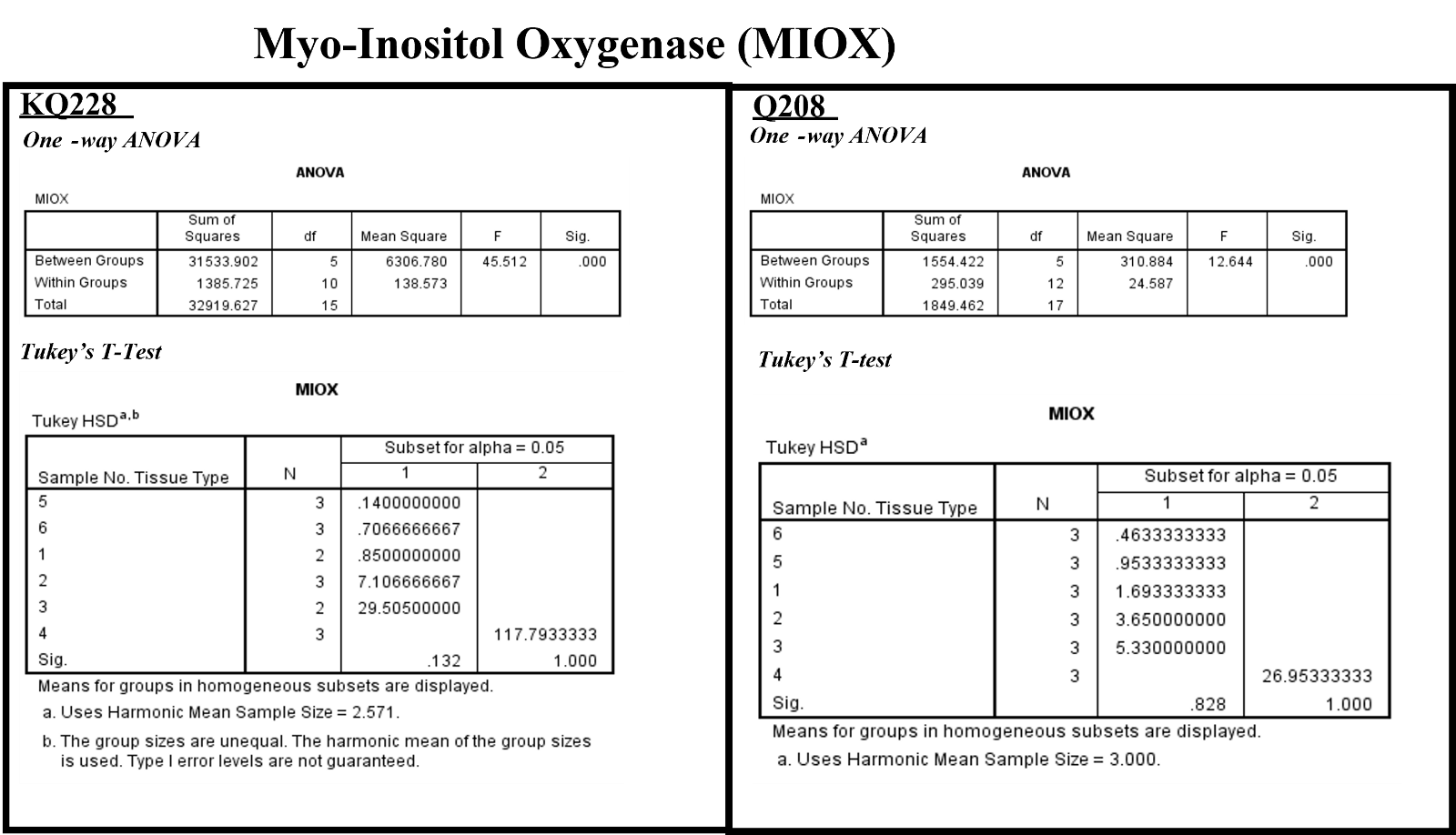


**Figure S23.** One-way ANOVA and Tukey *t*-test results from MIOX gene family expression comparisons.


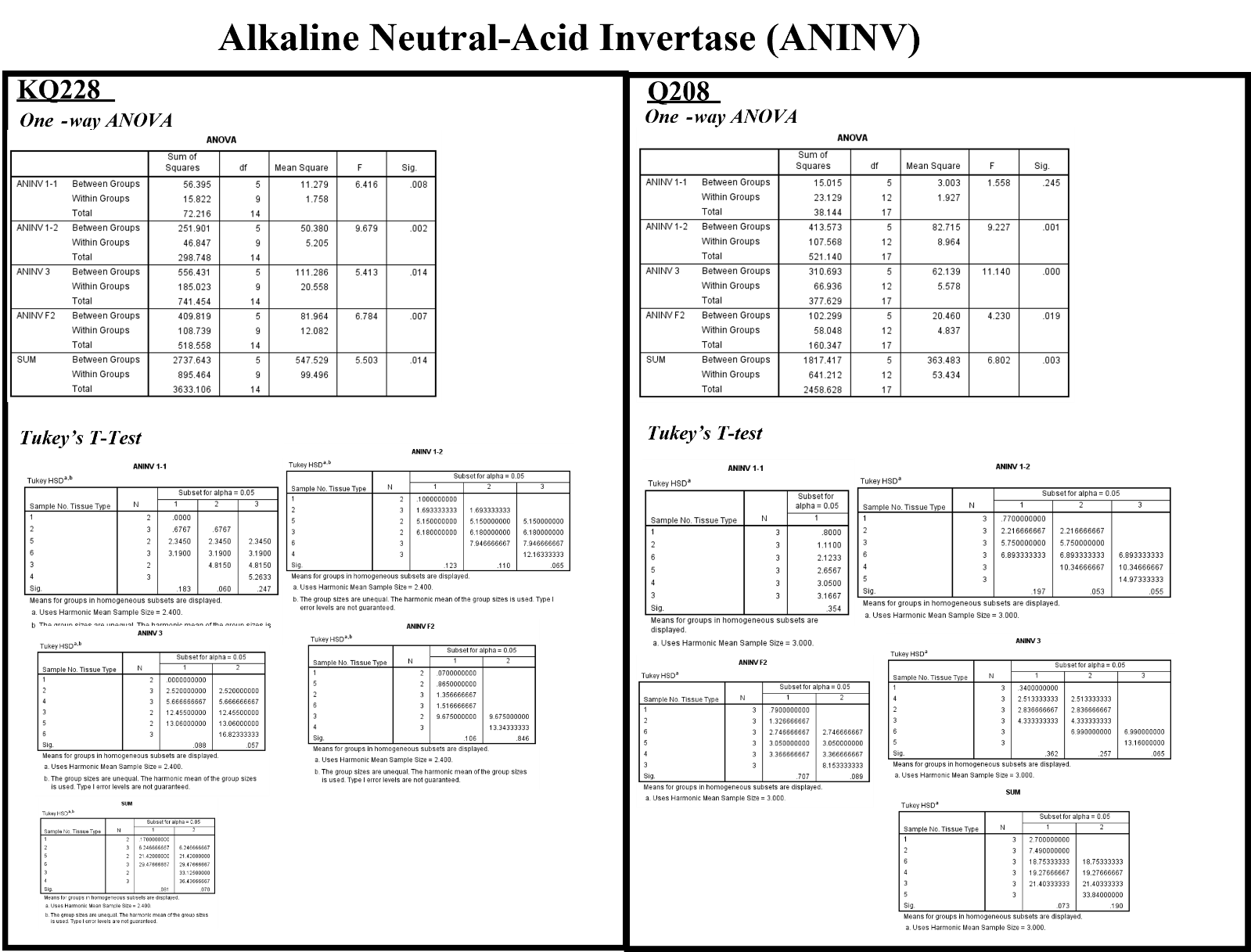


**Figure S24.** One-way ANOVA and Tukey *t*-test results from Invertase gene family ANINV.


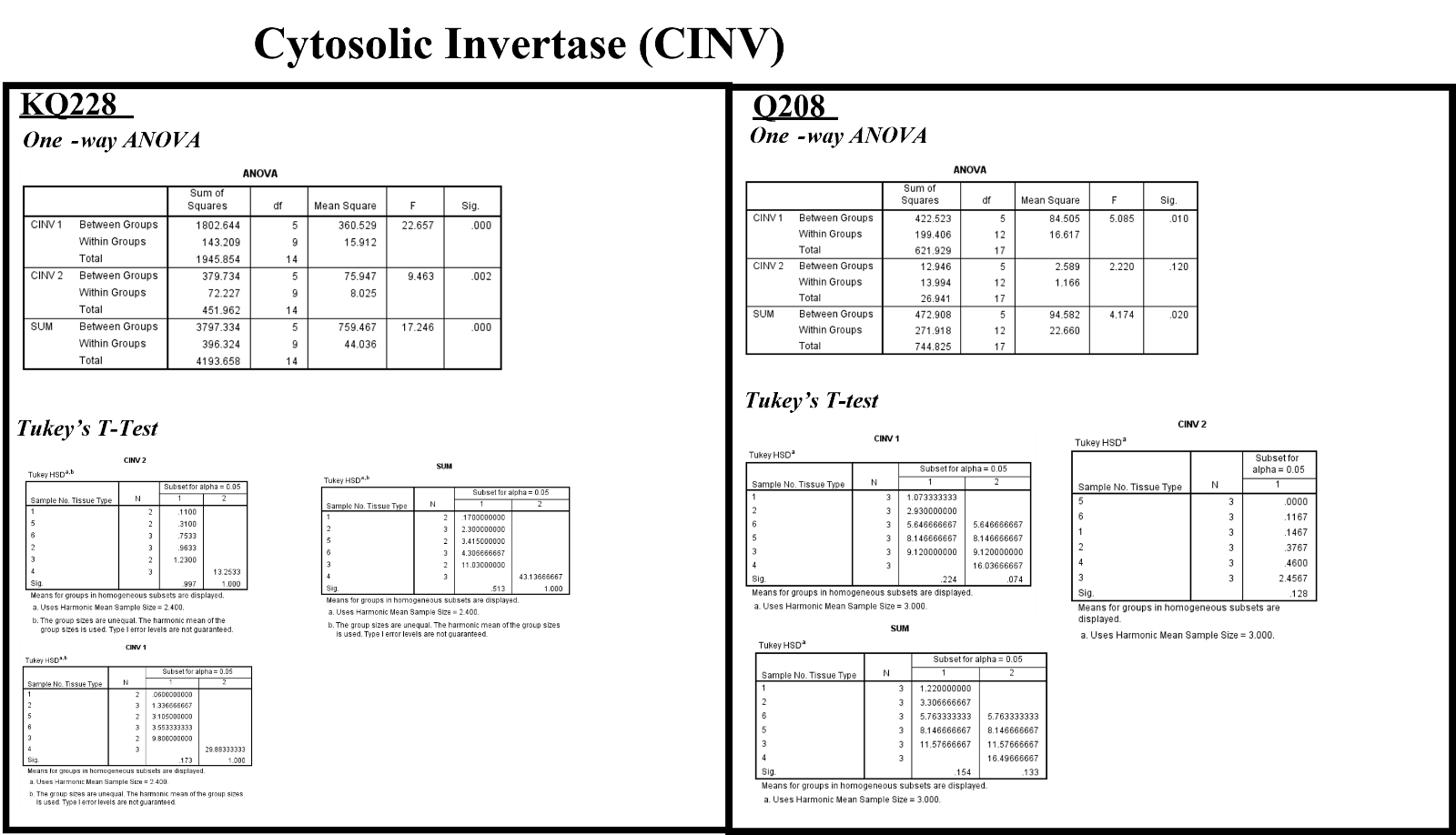
**Figure S25.** One-way ANOVA and Tukey *t*-test results from Invertase gene family CINV.


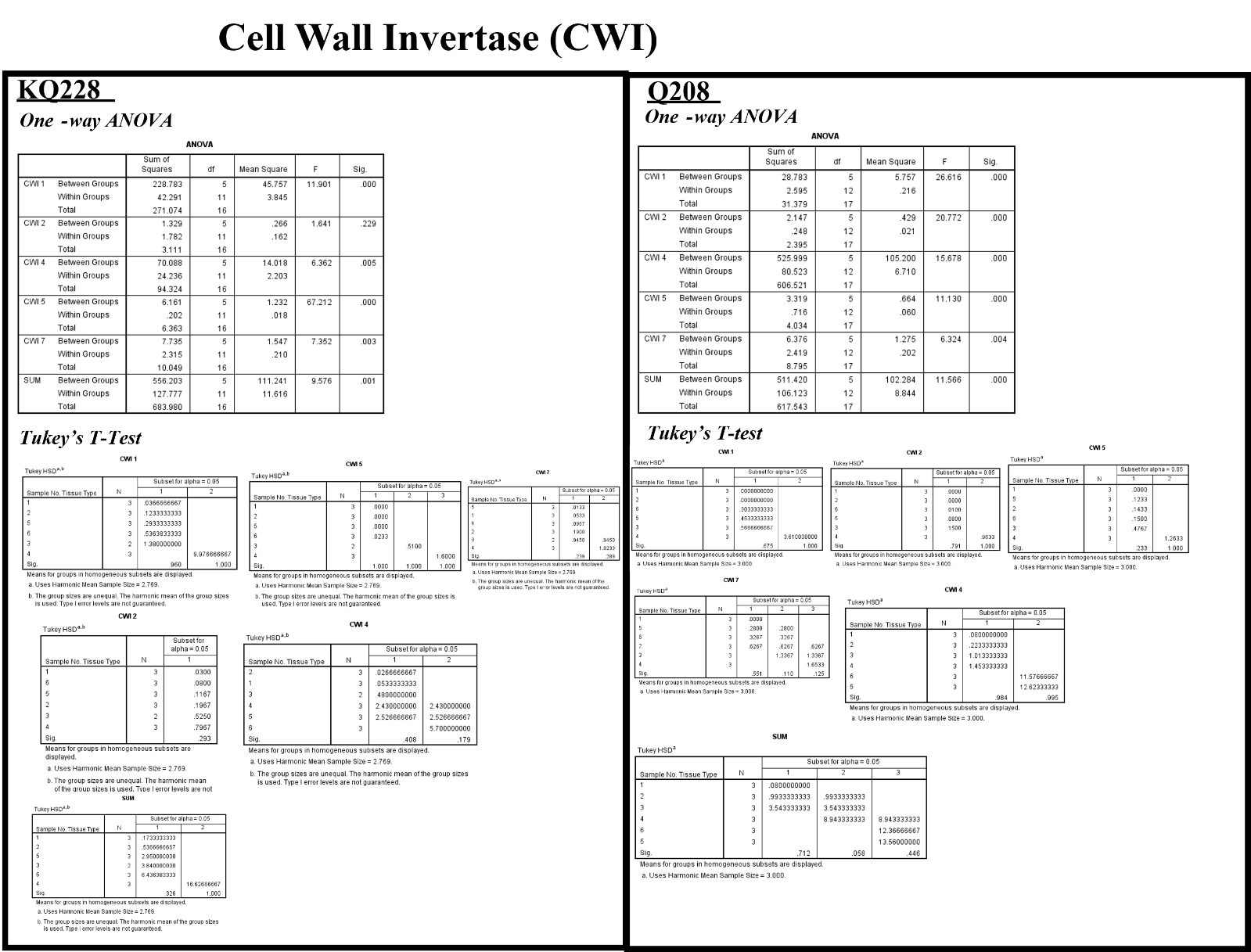


**Figure S26.** One-way ANOVA and Tukey *t*-test results from Invertase gene family CWI.


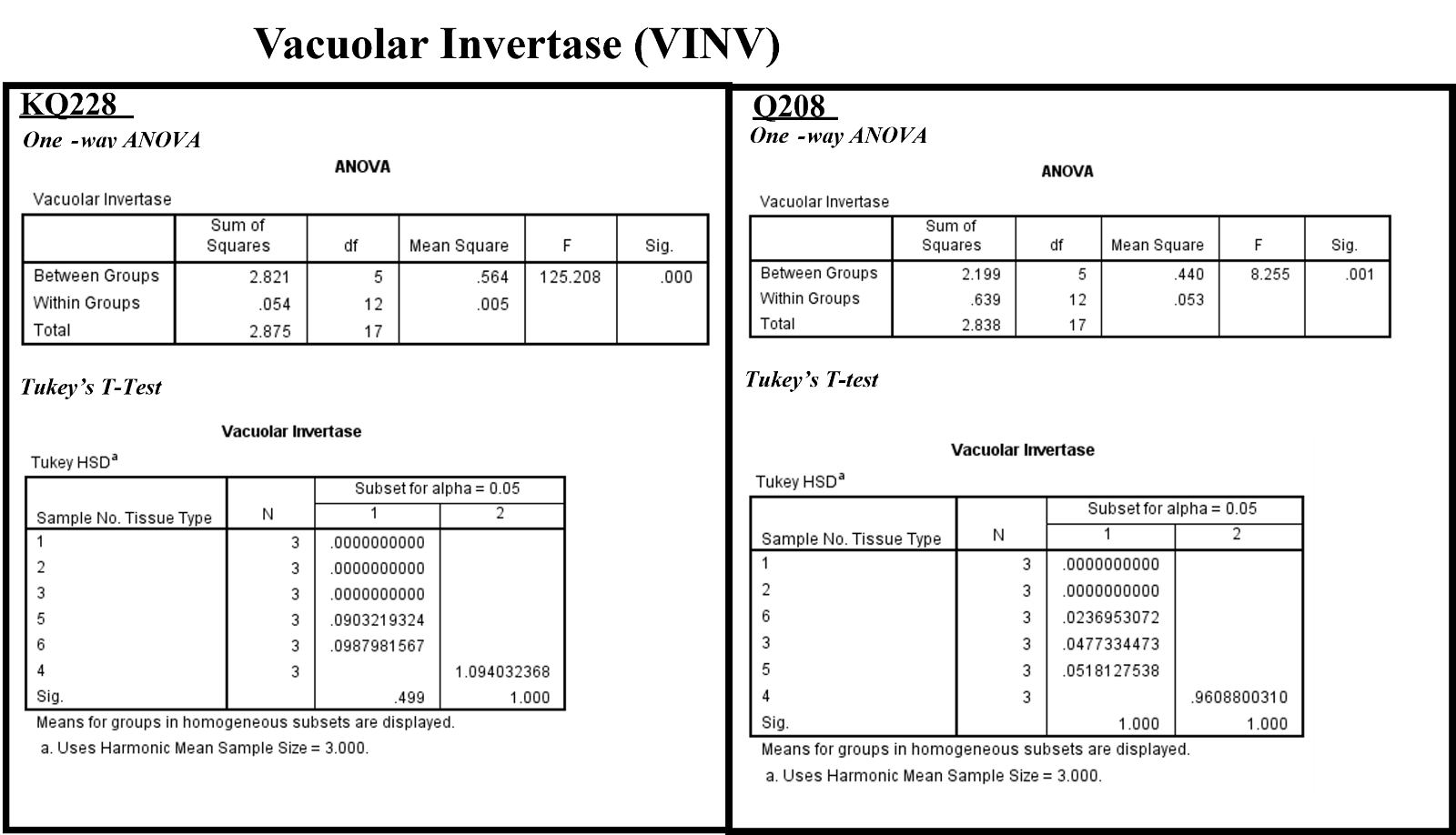


**Figure S27.** One-way ANOVA and Tukey *t*-test results from Invertase gene family VINV.


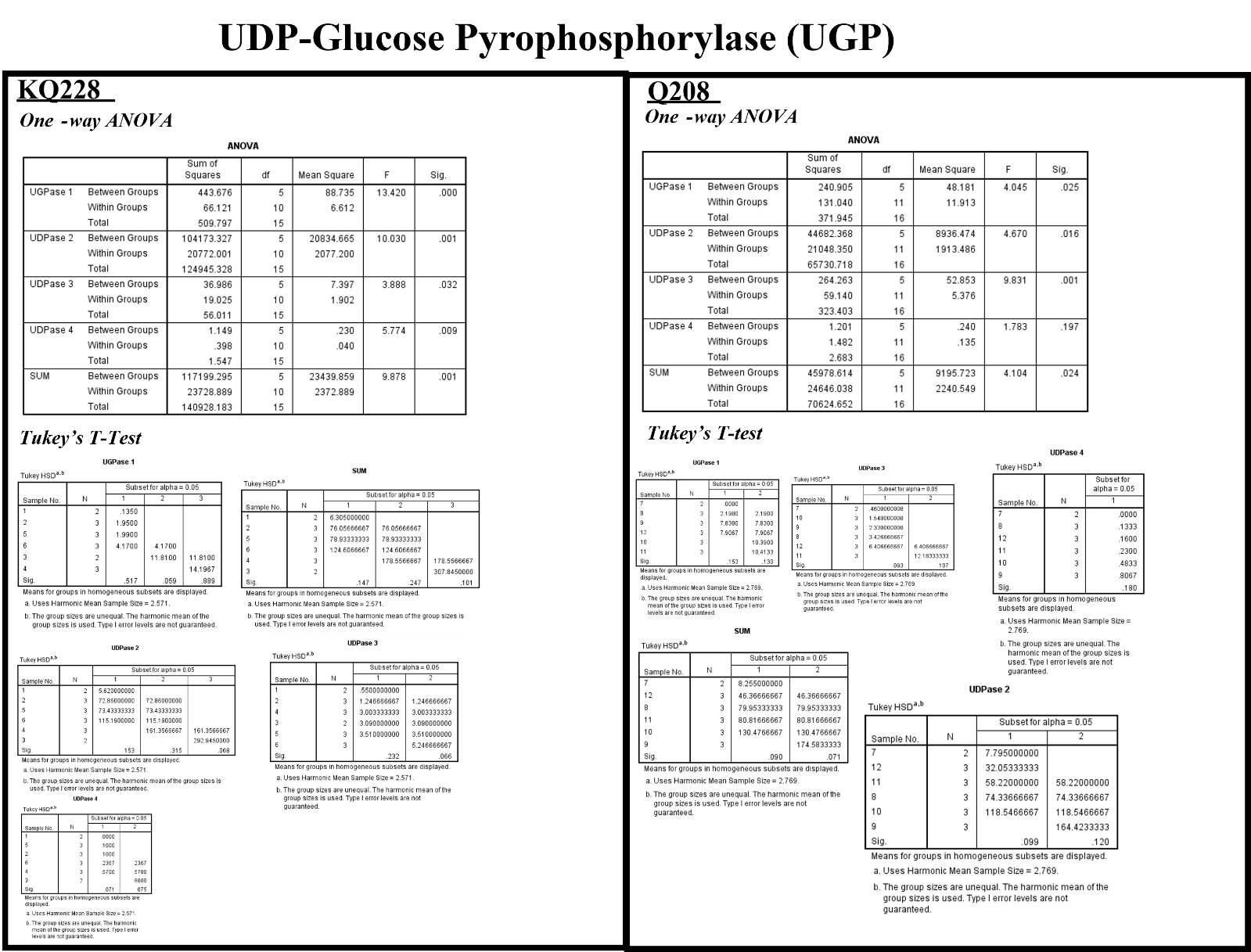


**Figure S28.** One-way ANOVA and Tukey *t*-test results from UGPase gene family expression comparisons.


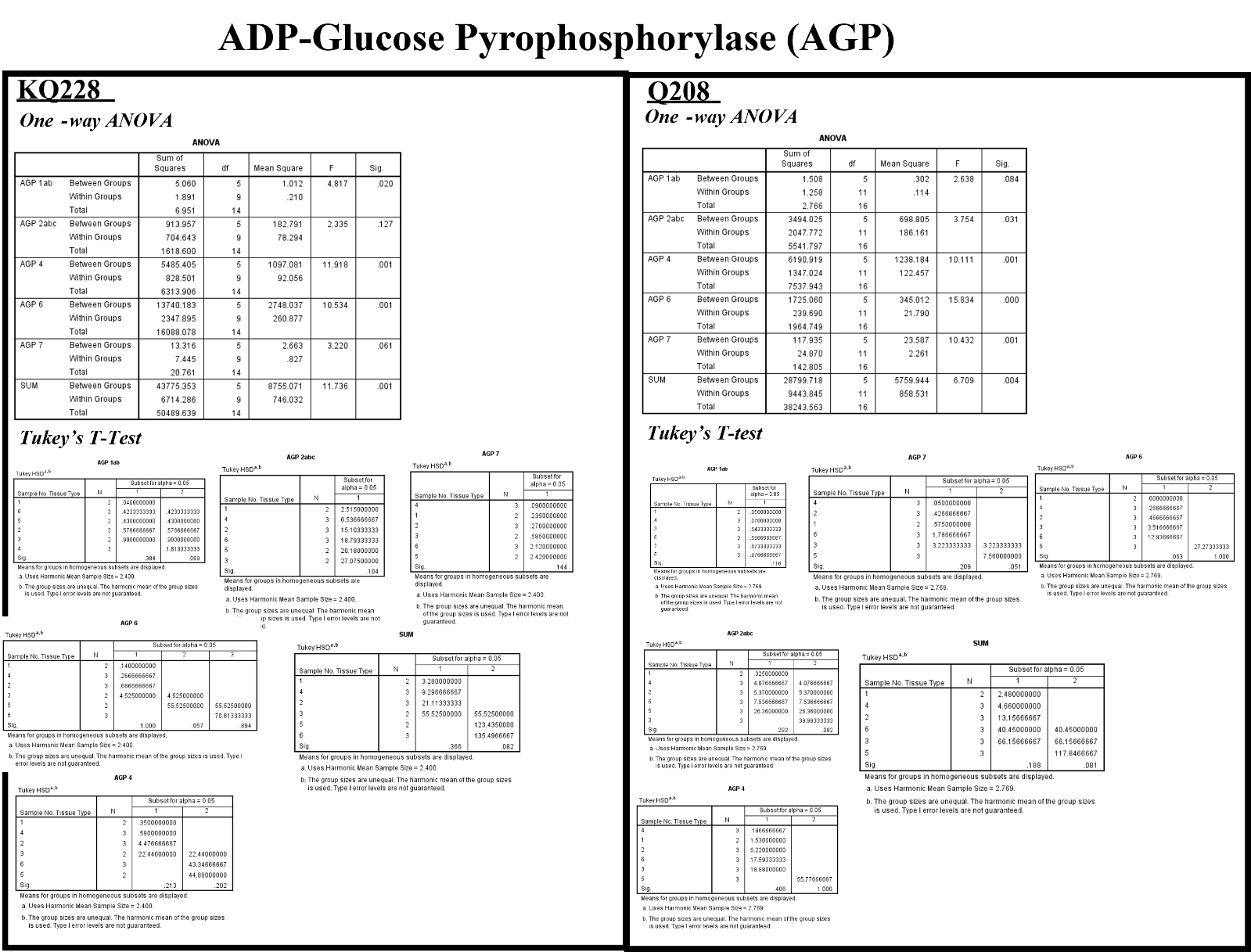


**Figure S29.** One-way ANOVA and Tukey *t*-test results from AGP gene family expression comparisons.


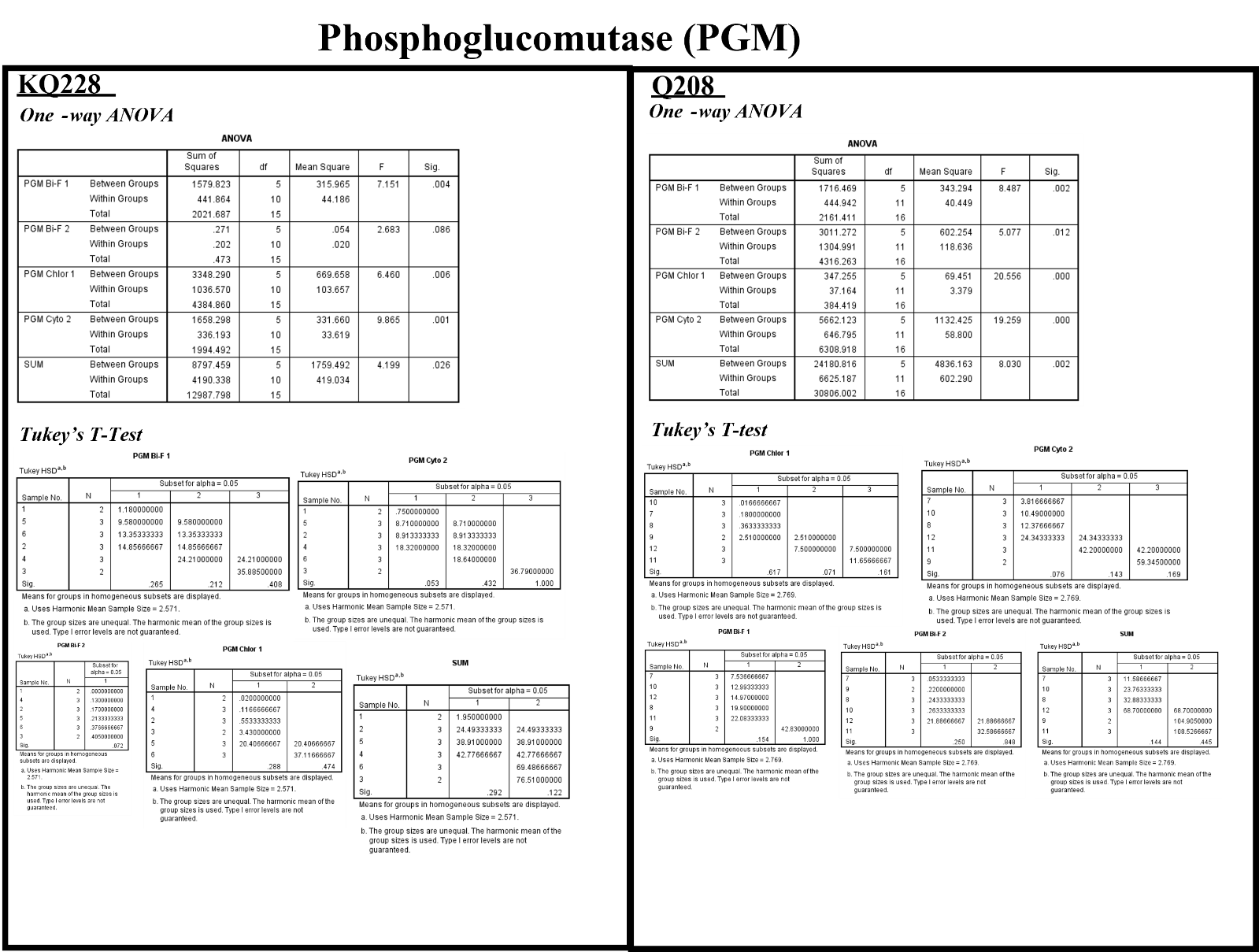


**Figure S30.** One-way ANOVA and Tukey *t*-test results from PGM gene family expression comparisons.


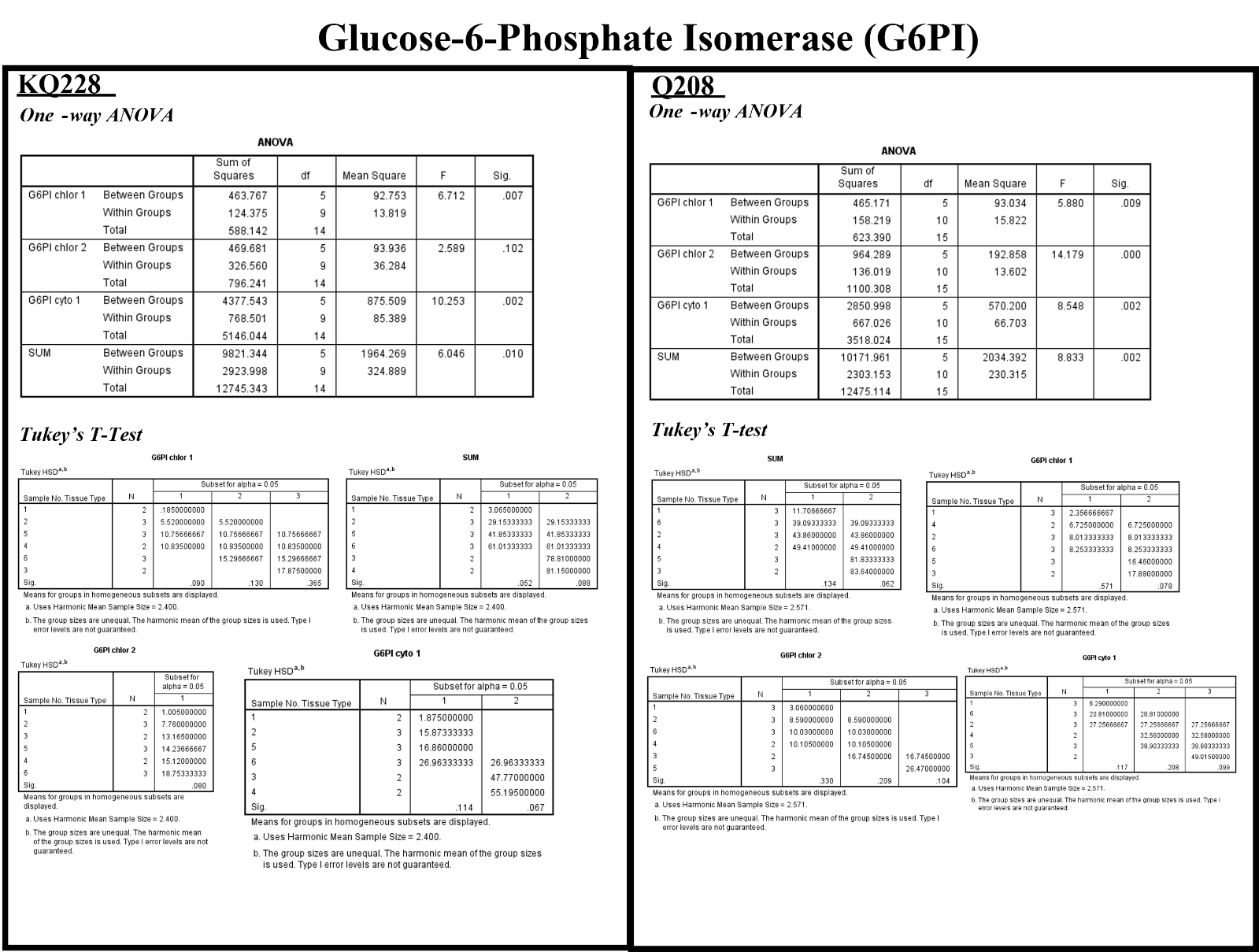


**Figure S31.** One-way ANOVA and Tukey *t*-test results from G6PI gene family expression comparisons.


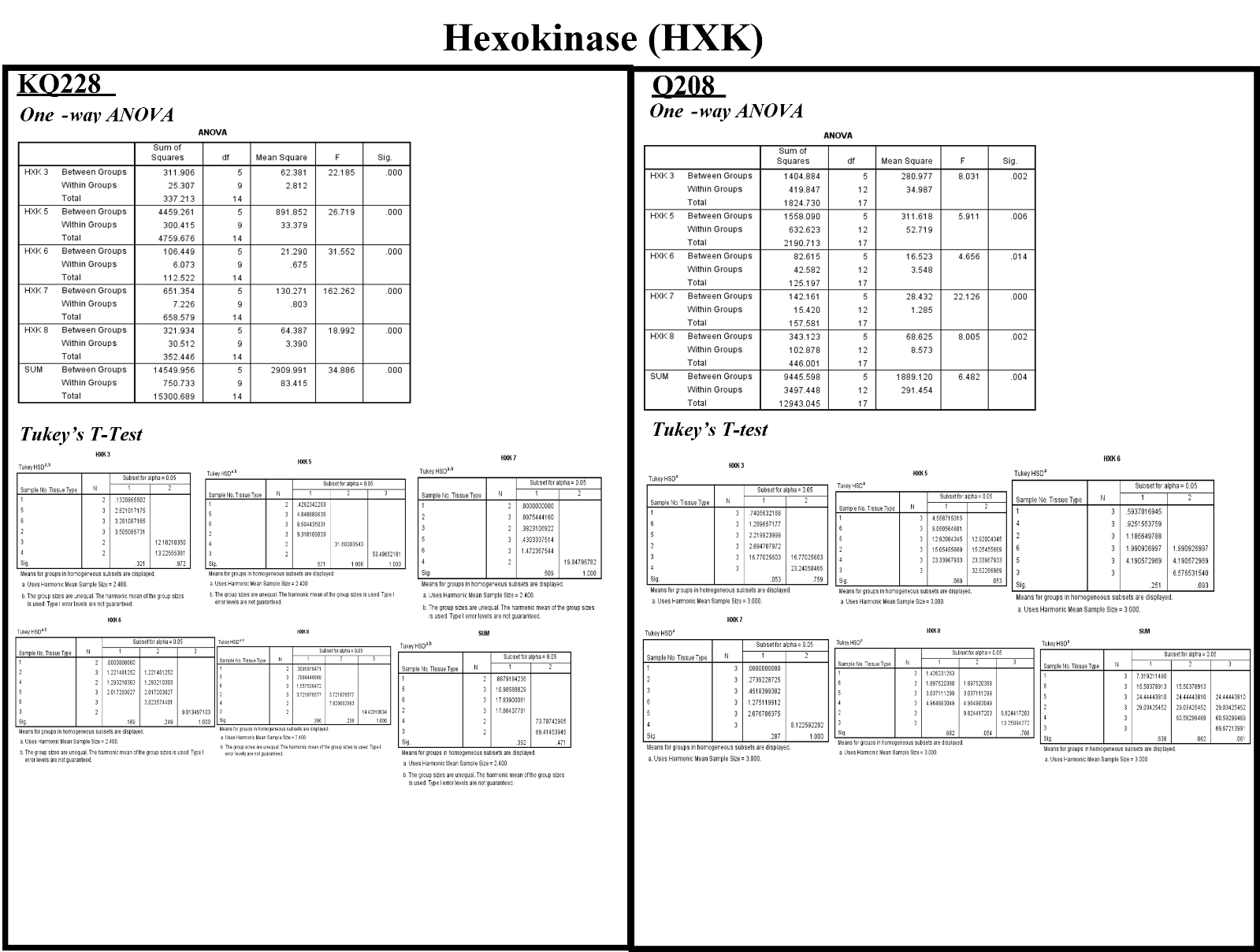


**Figure S32.** One-way ANOVA and Tukey *t*-test results from HXK gene family expression comparisons.


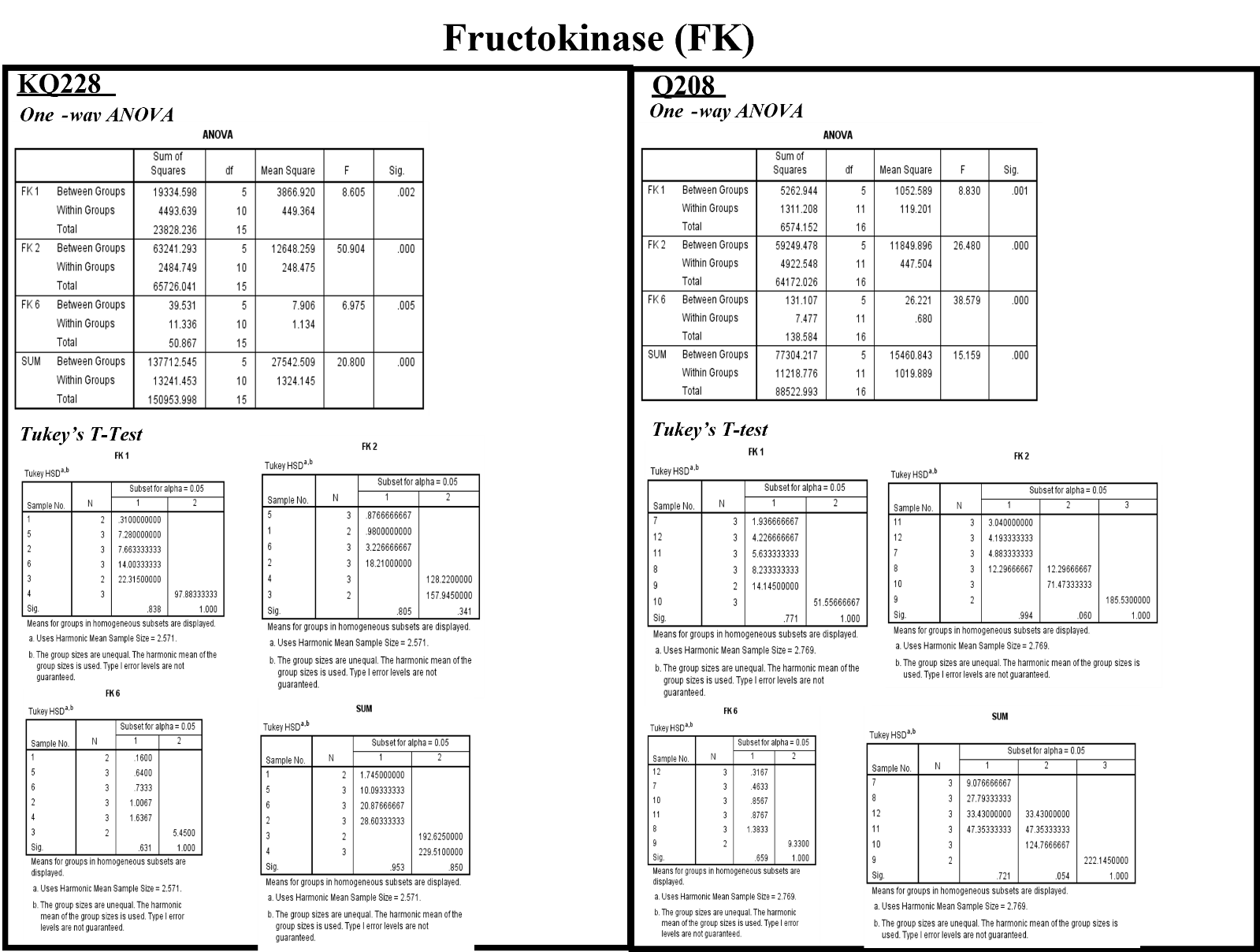


**Figure S33.** One-way ANOVA and Tukey *t*-test results from FK gene family expression comparisons.
